# Metal-ligand interactions in a redox active ligand system. Electrochemistry and spectroscopy of [M(dipyvd)_2_]^n+^ (M=Zn, Ni, *n*=0, 1, 2)

**DOI:** 10.3389/fchem.2023.1295289

**Published:** 2023-11-15

**Authors:** Connor Fleming, Son Vu, David J. R. Brook, Stefano Agrestini, Eric Pellegrin, Jeffrey DaRos

**Affiliations:** ^1^ Department of Chemistry, San Jose State University, San Jose, CA, United States; ^2^ ALBA Synchrotron Light Source, E-08290 Cerdanyola del Vallès, Barcelona, Spain

**Keywords:** radical ligands, verdazyl, redox-active ligands, mixed-valence, x-ray spectroscopy, nickel, zinc

## Abstract

Reaction of nickel and zinc triflates with the tridentate leucoverdazyl 1-isopropyl-3,5-di (2′-pyridyl)-6-oxo-2H-tetrazine (dipyvdH) and triethylamine resulted in the neutral coordination compounds M(dipyvd)_2_ (M = Ni,Zn). In acetonitrile, both compounds undergo two one electron oxidation processes, Zn (dipyvd)_2_ at −0.28 V and −0.12 V and Ni(dipyvd)_2_ at −0.32 V and −0.15 V vs ferrocene/ferricenium. Oxidations are ligand based resulting in an intermediate mixed valence species and a cationic bis(verdazyl) compound respectively. Oxidation of the ligand changes a localized, antiaromatic, non-planar 8π electron anion to a planar, delocalized 7π electron radical. The change in ligand structure results in an increase in the octahedral ligand field splitting from 10,500 cm^–1^ to ∼13,000 cm^–1^, suggesting an increase in the pi acceptor character of the ligand. In the mixed valence species, spectroscopic data suggests minimal interaction between ligands mediated by the metal center; i.e., these are class I-II systems in the Robin-Day classification.

## 1 Introduction

Transition metal compounds with open shell ligands have become increasingly important for a number of reasons. Open shell ligands are typically redox active which provides opportunities to enhance and tune the reactivity of the metal center, in particular this can result in modified catalytic activity (Singh et al., 2022). Interaction of the electron spins on metal and ligand can result in unusual magnetic properties, including the possibility of long range order (Caneschi et al., 1989). The combination of redox active ligands and redox active metal centers can also result in unusual electronic structures and equilibria exemplified by the observation of valence tautomerism in the metal complexes of oxolene ligands (catecholates, semiquinones and quinones) (Pierpont, 2001). The interaction between metal ion and ligand plays a key role in understanding when and how these phenomena can occur. When considering the interaction between transition metals and coordinated ligands, the focus is typically on the change in electronic structure of the metal ion. Though obviously there are changes in the ligand (such as changes in pKa) as a result of metal coordination, the ligand orbitals are typically far removed in energy from the metal *d* orbitals and they are only slightly perturbed, retaining their mostly ligand character. When the ligands themselves can be open shell species things are more complicated. The ligand SOMO must be of a similar energy to the metal *d* orbitals otherwise the ligand would likely be oxidized or reduced by the metal center; however, the ligand frontier orbitals may retain their essential localized character or may be delocalized onto the metal ion giving so-called non-innocent ligands*.* For example, while many nitronyl-nitroxide coordination compounds show significant magnetic exchange interactions, the ligand orbitals are only weakly perturbed by the metal ion and they can be described largely in terms of distinct metal and ligand based orbitals (Caneschi et al., 1991). Conversely with coordination compounds of thiolenes, though the overall species may be open shell, the orbitals are so delocalized that it is challenging to describe the ligands specifically as radicals or assign a clear oxidation state to the metal ion (Eisenberg and Gray, 2011).

Verdazyl ligands provide a mixture of examples where the radical ligand varies from being weakly to strongly perturbed by the metal center (Lipunova et al., 2022). In many cases these compounds feature strong metal-ligand exchange interactions, while the ligands clearly retain their radical character. Computational studies have implicated ligand → metal pi donor interactions playing an important role in magnetic exchange (Rota et al., 2008); experimentally, verdazyls coordinated to copper(I) have shown both pi acceptor and pi donor characteristics depending on the ancillary ligands on the metal ion (Brook and Abeyta, 2002). In certain systems the metal-ligand interactions are more complex with the ligand playing a less innocent role. Hicks and co-workers reported several verdazyl ruthenium systems in which the ligand ‘non-innocence’ is dependent upon the ancillary ligands on ruthenium (McKinnon et al., 2010; McKinnon et al., 2013). More recently we reported that the iron and cobalt complexes of the tridentate verdazyl ligand **dipyvd** exhibit electronic lability and non-innocent behavior (Brook et al., 2018; Fleming et al., 2020). In particular [Co(dipyvd)_2_]^2+^ shows valence tautomerism between an *S* = 3/2 Co(II)-diradical state and an *S* = 1/2 Co(III)-radical-anion state. The high spin state itself shows a complex electronic structure resulting from the mixing of states with differing local spins but the same overall value of *S*, referred to as ‘spinmerism’ (Roseiro et al., 2022). [Fe (dipyvd)_2_]^2+^ does not show thermal spin crossover, but does show spin crossover upon one electron reduction to give a high spin species in which the additional electron appears to be delocalized over the whole molecule. Computational studies also suggest that [Fe (dipyvd)_2_]^2+^ shows a version of spinmerism in its excited state. (Roseiro et al., 2023).

In order to better understand the metal-ligand interactions of verdazyl ligands in general, and the **dipyvd** ligand in particular we report here a study of the compounds of the same ligand with zinc, and a more detailed investigation of the nickel species we reported in 2010 (Richardson et al., 2010). While the nickel species shows strong magnetic exchange, neither of these species are expected to show electronic lability. Nevertheless they provide reference points for the influence of the metal ion on the redox chemistry of the ligand, and the effects of the radical and anionic ligand on the *d*-orbital splitting of the metal ion, both of which are relevant for understanding electronic lability. Similar studies have been useful for the understanding of spin crossover compounds (Klaeui et al., 1987), and the spectroscopy of *d* ^6^ transition metal ions (Yarranton and McCusker, 2022). By developing our understanding of the **dipyvd** ^**•**^/**dipyvd** ^**–**^ligand system, and verdazyl radical ligands more generally, we hope to create a more comprehensive model to describe the behavior of other metal complexes of this ligand and develop strategies to fine tune their electronic structure.

## 2 Experimental

### 2.1 General

Cyclic voltammetry employed a platinum disk working electrode, platinum wire counter electrode, and silver/silver chloride pseudo-reference electrode. Potentials were referenced to internal ferrocene. The compound [Ni(dipyvd)_2_](PF_6_)_2,_ has been reported previously (Richardson et al., 2010). X-ray absorption spectra and X-ray magnetic circular dichroism were recorded at the BOREAS beamline, ALBA synchrotron, Barcelona, Spain. Simulations of X-ray spectra were performed with the program *CRISPY* (Retegan, 2019). Simulations of ESR spectra and modeling magnetic data used the MATLAB package *Easyspin* (Stoll and Schweiger, 2006). DFT geometry optimizations were performed with the package ORCA 5.0.1 (Neese, 2012). Calculations used the PBEh-3c (Grimme et al., 2015; Janetzki et al., 2021) functional and the def2-TZVP basis set. Output files and final atomic coordinates are provided as Supporting Material.

### 2.2 1-Isopropyl-3,5-di (2′-pyridyl)-6-oxo-2H-tetrazine (dipyvdH)

The synthesis of this compound has been reported previously (Brook et al., 2018). Single crystals were obtained by slow evaporation of a solution in dichloromethane/heptane and examined by X-ray crystallography. Unit cell dimensions and select parameters and statistics are reported in Table 1. Full details of data collection and refinement are available from the Cambridge Crystallographic Data Centre in. cif format.

**TABLE 1:** Crystallographic data.

Species	dipyvdH	Ni(dipyvd)_2_	[Ni(dipyvd)_2_](PF_6_)_2_ ^a^	Zn (dipyvd)_2_	[Zn (dipyvd)_2_](PF_6_)_2_
CCDC deposition number	2,267,079	2,267,261	753,408	2,267,243	2,267,027
Measurement Temperature/K	298	150	100	150	299
Crystal System	monoclinic	trigonal	tetragonal	trigonal	tetragonal
Spacegroup	*P*2_1_/*c*	*P*3_1_21	$P\overset{—}{4}2_{1}c$	*P*3_1_21	$P\overset{—}{4}2_{1}c$
*a*	12.5752(7)	14.0746 (13)	15.800(6)	14.0975(8)	15.8591(6)
*b*	14.9127(9)	—	—	—	—
*c*	8.1051(5)	26.085(2)	15.227(6)	26.2314(17)	15.6583(8)
*α*	90°	90°	90°	90°	90°
β	91.075°(2)	90°	90°	90°	90°
γ	90°	120°	90°	120°	90°
*Z*	4	6	4	6	4
*R*_*1*_	7.24	12.85	4.60	6.32	5.65
N1-C2	1.373(3)	1.39(3)^d^	*c*	1.374 (12)^d^	*c*
C2-N4	1.282(3)	1.27(2)^d^	*c*	1.297 (11)^d^	*c*
N4-N3	1.410(3)	1.44(2)^d^	*c*	1.445 (10)^d^	*c*
N1-N2	1.434(3)	1.45(2)^d^	*c*	1.433 (10)^d^	*c*
M-N1	n/a	1.959 (14)^d^	1.978(5)	2.006(7)^d^	2.080(3)
M-N5	n/a	2.111 (19)^d^	[2.113(5)]^b^	2.193 (10)^d^	[2.184(3)]^b^
M-N6	n/a	2.114 (16)^d^	[2.104(5)]^b^	2.176(8)^d^	[2.176(4)]^b^

^a^ Data from ref. 11.

^b^ These bond lengths are weighted averages of the two metal-pyridyl bond lengths as a result of disorder in the crystal structure.

^c^ Meaningful bond lengths cannot be provided because of orientational disorder in the crystal structure.

^d^ Bond lengths refer to the non-disordered molecule in the structure.

### 2.3 Zn (dipyvd)_2_

DipyvdH (159.0 mg, 0.533 mmol) was placed in a 25 mL beaker along with zinc trifloromethanesulfonate (74.1 mg, 0.273 mmol). Enough acetonitrile was added in order to completely dissolve the solids (approx 6 mL) to give an orange-red solution. The solvent was then evaporated to leave an orange-red solid. The solid was redissolved in methanol and triethylamine was added dropwise (10 drops) to the clear red solution. A precipitate formed and was removed by vacuum filtration to give the product as a red powder; 106.8 mg (0.161 mmol, 64.4%). ^1^H NMR (300 MHz, Chloroform-*d*) δ 8.48 (d, *J* = 8.9 Hz, 2H), 8.20–8.09 (m, 4H), 7.84 (ddd, *J* = 5.3, 1.9, 0.9 Hz, 2H), 7.71 (td, *J* = 7.7, 1.6 Hz, 2H), 7.47 (ddd, *J* = 9.0, 7.0, 1.9 Hz, 2H), 7.21–7.10 (m, 2H), 6.63–6.52 (m, 2H), 4.85 (hept, *J* = 6.6 Hz, 2H), 1.44 (d, *J* = 6.6, 6H), 1.43 (d, *J* = 6.6, 6H); ^13^C NMR (75 MHz, CDCl_3_) δ175.5, 150.2, 149.5, 149.2, 145.9, 144.4, 137.7, 137.6, 124.6, 120.8, 115.2, 114.3, 47.2, 20.1; IR (ATR) 1,660 cm^–1^ (C=O); UV-vis (CH_2_Cl_2_) λ/nm (ε/L.mol^–1^cm^–1^) 282 (14,000), 340 (10,000). Single crystals were grown by layering a dichloromethane solution with heptane and allowing layers to diffuse together. X-ray diffraction data were collected at beamline 11.3.1 at the Advanced Light Source (ALS) under the SCrALS program. Unit cell dimensions and select parameters and statistics are reported in Table 1. Full details of data collection and refinement are available from the Cambridge Crystallographic Data Centre in. cif format.

### 2.4 Ni(dipyvd)_2_

DipyvdH (29 mg, 0.1 mmol) was dissolved in 1 mL methanol and added to a solution of nickel trifluoromethanesulfonate (21 mg, 0.05 mmol) in 1 mL methanol. To the resulting orange solution was added 3 drops of triethylamine followed by 2 mL distilled water. The resulting orange-red precipitate of Ni(dipyvd)_2_ was removed by filtration and air dried to give 25 mg (0.04 mmol, 77%). IR (ATR) 1,658 cm^–1^ (C=O); UV-vis (CH_2_Cl_2_): λ/nm (ε Lmol^–1^cm^–1^) 934 (90), 348 (13,400), 284 (19,400), 252 (21,000). Single crystals were grown by layering a dichloromethane solution with heptane and allowing the layers to diffuse together. Though the overall crystal quality was low, unit cell determination indicated that the material was isostructural with the corresponding zinc complex. Unit cell dimensions and select parameters and statistics are reported in Table 1. Full details of data collection and refinement are available from the Cambridge Crystallographic Data Centre in. cif format.

### 2.5 [Zn (dipyvd)_2_](PF_6_)_2_

Zn (dipyvd)_2_ (50 mg, 0.076 mmol) was placed in a 25 mL beaker and dissolved in dichloromethane. Silver hexafluorophosphate (37 mg, 0.192 mmol) dissolved in 10 mL dichloromethane was added. The resulting red/brown precipitate was collected using vacuum filtration, redissolved in acetonitrile and filtered to remove metallic silver. Subsequently the product was precipitated by diffusion of ether vapor into the solution. After 2 days, magenta crystals were observed at the bottom of the vial (29.5 mg, 40.8%). IR (ATR), 1725 cm^-1^ (C=O), 1,600 cm^-1^. UV-vis (CH_3_CN) λ/nm (ε/L.mol^–1^cm^–1^) 450 (2,700), 546 (2,800), 582 (3,900). Calcd for C_30_H_30_F12N_12_O_2_P_2_Zn: C 38.09, H 3.20, N, 17.77; found: C 37.27, H 2.86, N 17.62. A single crystal was selected for X-ray analysis. Unit cell dimensions and select parameters and statistics are reported in Table 1. Full details of data collection and refinement are available from the Cambridge Crystallographic Data Centre in. cif format.

## 3 Results

### 3.1 Zinc

**SCHEME 1:**
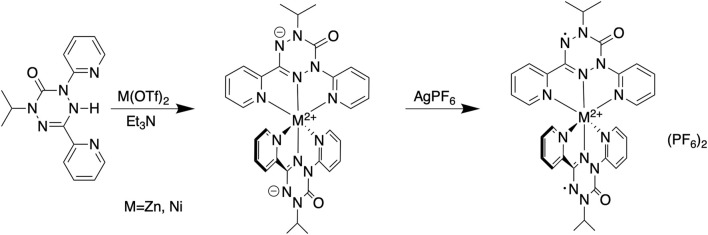
Synthesis of M(dipyvd)_2_ and [M(dipyvd)_2_](PF_6_)_2_.

**FIGURE 1:**
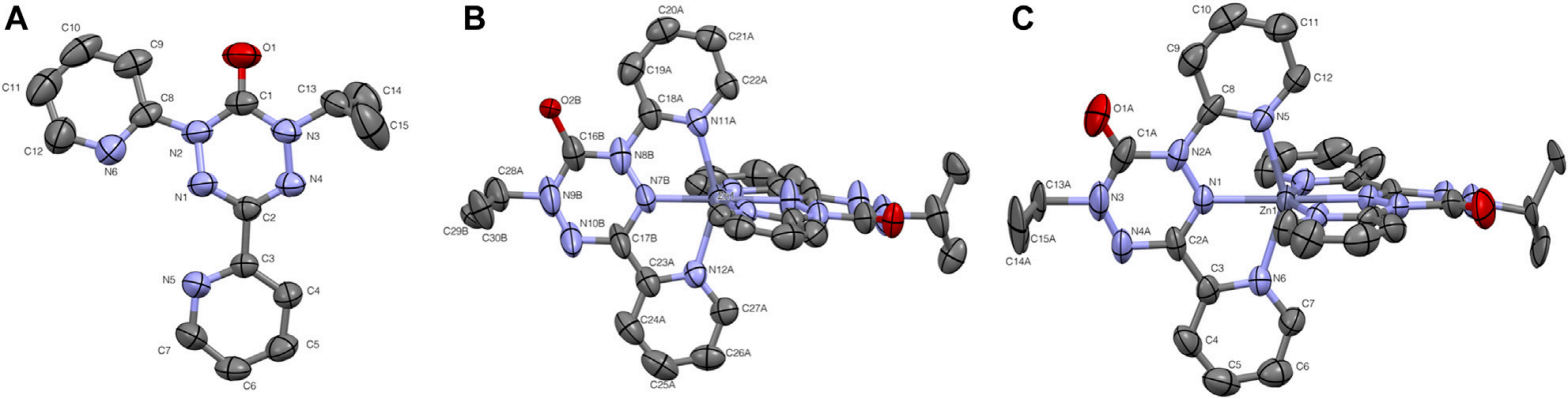
Thermal ellipsoid plots for **(A)** pyvdH, **(B)**. Zn (dipyvd)_2_ and **(C)** [Zn (dipyvd)_2_](PF_6_)_2_. Ellipsoids are drawn at 50% probability. For B only one of the two independent molecules in the unit cell is shown. For B and C the two ligands are related by symmetry; atomic labels for only one ligand are shown. Hydrogen atoms and PF_6_ counterions are not shown for clarity.

The syntheses of the two zinc compounds (and their nickel analogs) are shown in Scheme 1. Combination of dipyvdH and zinc triflate in acetonitrile, followed by addition of excess triethylamine gave the orange-red, neutral, diamagnetic zinc complex Zn (dipyvd)_2_. Orange hexagonal crystals were grown by layering a dichloromethane solution with heptane and examined by X-ray diffraction. The structure was solved in the space group *P*3_1_21 with two independent molecules in the unit cell, each lying on a twofold symmetry axis. Though one of the molecules shows additional disorder about a non-crystallographic two fold axis, the other has only one orientation. Extensive orientational disorder in the structure prevents detailed examination of the ligand geometry; nevertheless the data confirms the proposed molecular structure with each zinc ion coordinated by two anionic ligands in a distorted octahedral coordination environment. Oxidation with silver hexafluorophosphate in dichloromethane gave the diradical hexafluorophosphate as a deep red solid. Single crystals of [Zn (dipyvd)_2_]^2+^(PF_6_)_2_ were grown by vapor diffusion or slow evaporation. The crystals were isomorphic with the previously reported nickel complex, (Richardson et al., 2010), crystallizing in the tetragonal space group $P\bar{4}2_{1}c$ . As with the nickel system, the cation is disordered around a non-crystallographic twofold axis, resulting in some ambiguity in the bond lengths, especially around the verdazyl ring. Crystal structure details and key bond lengths are presented in Table 1. Thermal ellipsoid plots are shown in Figures 1B,C.

**FIGURE 2:**
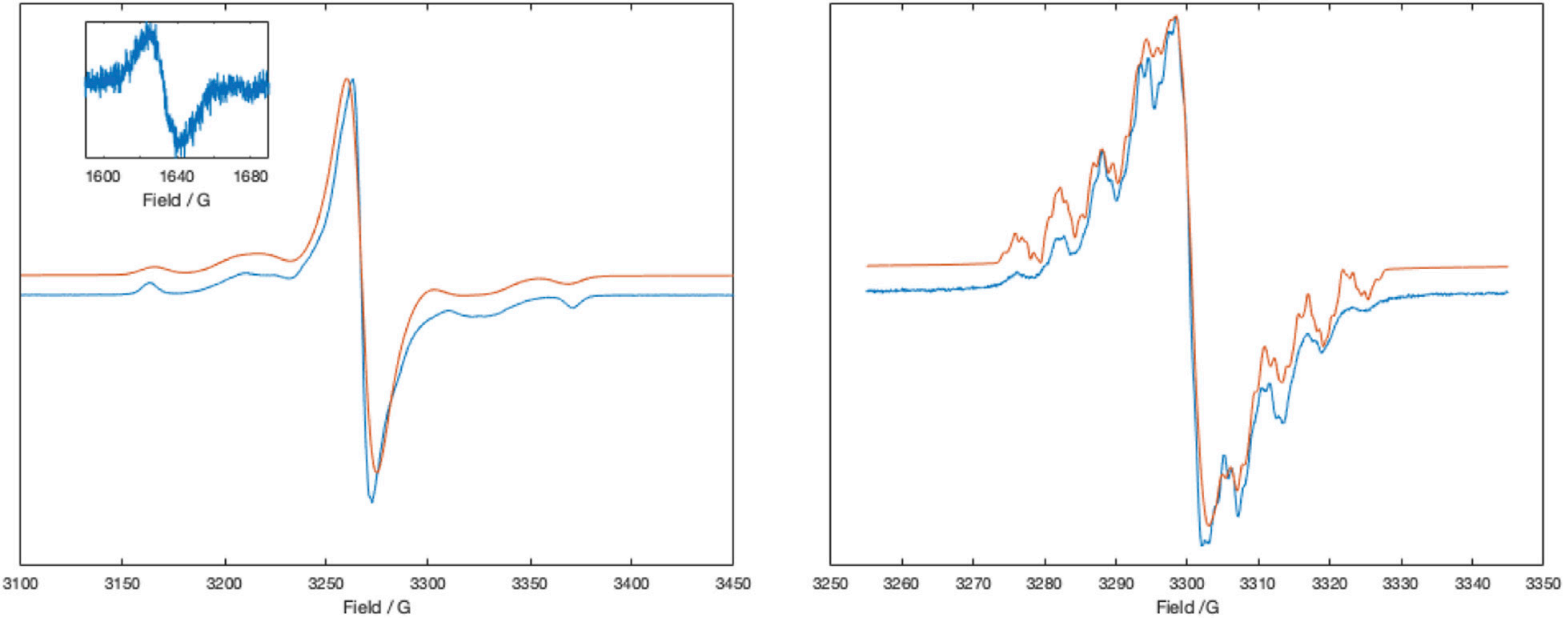
X-band ESR spectra of [Zn (dipyvd)_2_]^2+^ in frozen acetonitrile at 77 K (left) and the same solution at room temperature, partly reduced with cobaltocene (right). Inset shows the half field signal of the triplet. Experimental data is in blue, simulations are in red. Simulation parameters (77 K), Signal 1: S = 1, D = 0.0095 cm–1, E = 0.0007 cm–1, gx = 2.0032, gy = 2.0038, gz = 2.0043, linewidth = 1.5G, HStrain = [30,48,1], weight = 0.1; Signal 2: S = 1/2, g = 2.0041, linewidth (Voigtian 0.576, 2.26 G), weight = 0.8. Simulation parameters (RT) Signal 1. G = 2.0051, aH = 4.63,3.98, 4.7, 2.33 MHz, aN = 19.0, 17.9, 13.3, 12.7, 2.1 MHz, linewidth = 0.1G, weight = 0.5; Signal 2: g = 2.0052 linewidth (Voigtian 0.5, 0.8G), weight = 0.5.

Initial magnetic susceptibility measurements on [Zn (dipyvd)_2_]^2+^(PF_6_)_2_ gave a constant value of *χT* = 0.75 between 300K and 50 K corresponding to two weakly interacting S = 1/2 spins (Supporting Material). Comparison with the Bleany-Bowers equation suggests that any exchange interaction *J* is at most ±3 cm^–1^. The UV-vis of the complex in acetonitrile is almost the same as that previously reported for [Ni(dipyvd)_2_]^2+^(PF_6_)_2_. (Richardson et al., 2010). Similarly the IR spectrum of [Zn (dipyvd)_2_]^2+^ is almost indistinguishable from the nickel analog with a C=O stretch at 1725 cm^–1^, consistent with coordinated verdazyl ligand. Solutions of [Zn (dipyvd)_2_]^2+^ in acetonitrile gave a weak 9 line ESR spectrum at ambient temperatures which we attribute to a trace of dissociated ligand (Supporting Material). When frozen (77 K) a far stronger signal is observed consistent with the presence of a triplet species with parameters |D| = 0.0097 cm^–1^, E = 0.0007 cm^–1^ and a weak half field signal at 1630 G. The same spectrum also contains a strong signal at *g* = 2. Because the latter signal is not apparent in fluid solution, we attribute it to aggregates of the dication in which resolution of the zero field splitting is lost as a result of intermolecular exchange interactions (Figure 2A). The axial component *D*) of the zero field splitting parameters was reproduced by DFT calculations of the spin-spin dipolar interaction using the PBEh-3c functional. Calculated values were *D* = −0.0088 cm^–1^ and *E* = 0.00002 cm^–1^.

**FIGURE 3:**
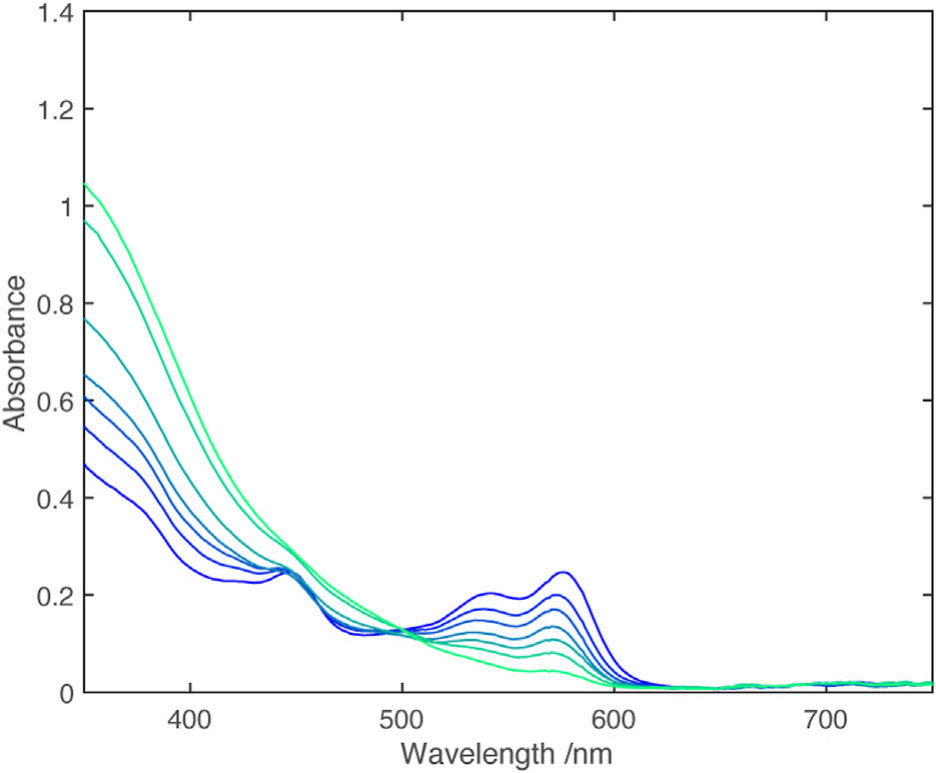
UV-vis spectra from electrochemical reduction of [Zn (dipyvd)_2_]^2+^ in acetonitrile. The working electrode potential ranges from −0.08 V vs. Fc/Fc+ (dark blue) to −0.32 V vs. Fc/Fc+ (light blue) in 0.04 V steps.

Cyclic voltammetry shows two closely spaced one electron reductions (Table 2); Monitoring the UV-vis spectrum during reduction revealed a near isosbestic point, (Figure 3), indicating that the spectrum of the singly reduced species is close to an average of the unreduced and fully reduced spectra.

**TABLE 2:** Electrochemical potentials for [M(dipyvd)_2_]^n+^ species (V vs. Ferrocene/Ferricenium).

	X^+^/X	X^2+^/X^+^
**[Ni**(**dipyvd)**_**2**_]^**n+**^	−0.32	−0.15
**[Zn**(**dipyvd)**_**2**_]^**n+**^	−0.28	−0.12

**FIGURE 4:**
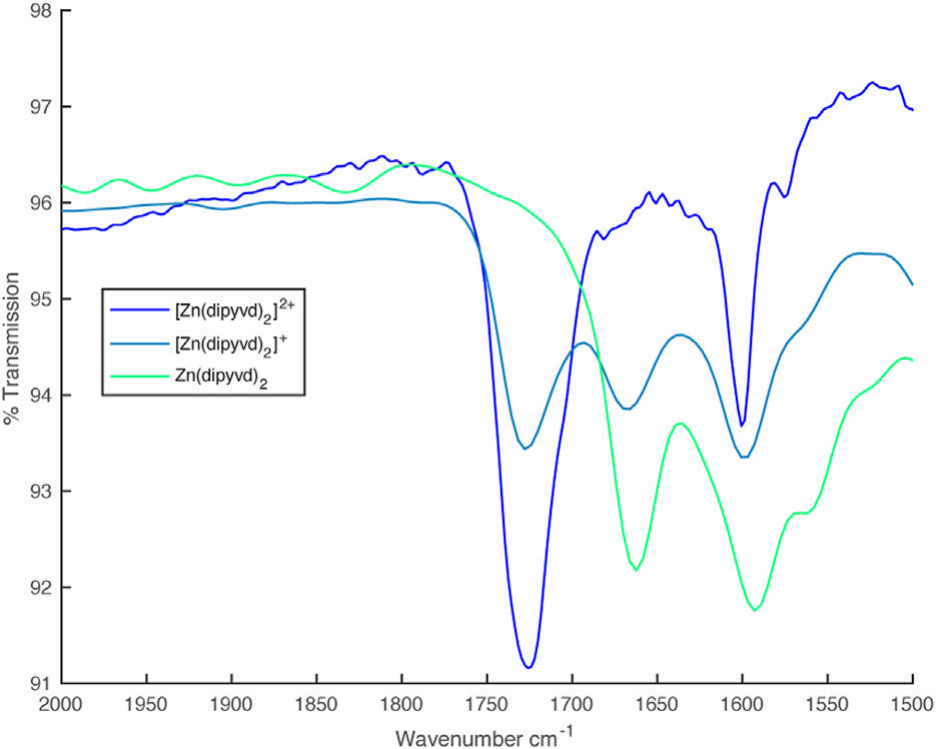
IR spectra from partial reduction of [Zn (dipyvd)_2_]^2+^ with cobaltocene.

In the IR spectrum, upon reduction with one equivalent of cobaltocene, an additional C=O stretch at 1,660 cm^–1^ appeared, which eventually replaced the 1725 cm^–1^ peak upon complete reduction with a second equivalent of CoCp_2_ (Figure 4).

An EPR signal with partially resolved hyperfine structure was observed in half reduced solutions (Figure 2B). The spectrum was simulated as the sum of two signals, one with resolved hyperfine coupling and the second a broad unresolved line that we attribute to partial precipitation of the monocation. Though simulation of the spectrum was not perfect, the hyperfine parameters are consistent with localization of the unpaired electron on a single ligand. The major hyperfine coupling constants are for the four nitrogen atoms in the verdazyl ring, with additional coupling to four protons which we identify as those in the pyridine rings (based on DFT calculations). These latter couplings are observed here but not in the spectrum of the ligand itself, probably because of the restricted conformation of the coordinated ligand. With a second equivalent of cobaltocene, a well resolved ^1^H NMR spectrum identical to that recorded for Zn (dipyvd)_2_ was observed.

Partly reduced solutions containing the [Zn (dipyvd)_2_]^+^ cation, spectroscopically identical to those generated with cobaltocene, were also obtained by combining equimolar amounts of [Zn (dipyvd)_2_]^2+^(PF_6_) and Zn (dipyvd)_2_ in a 50/50 mixture of dichloromethane and acetonitrile.

### 3.2 Nickel

**FIGURE 5:**
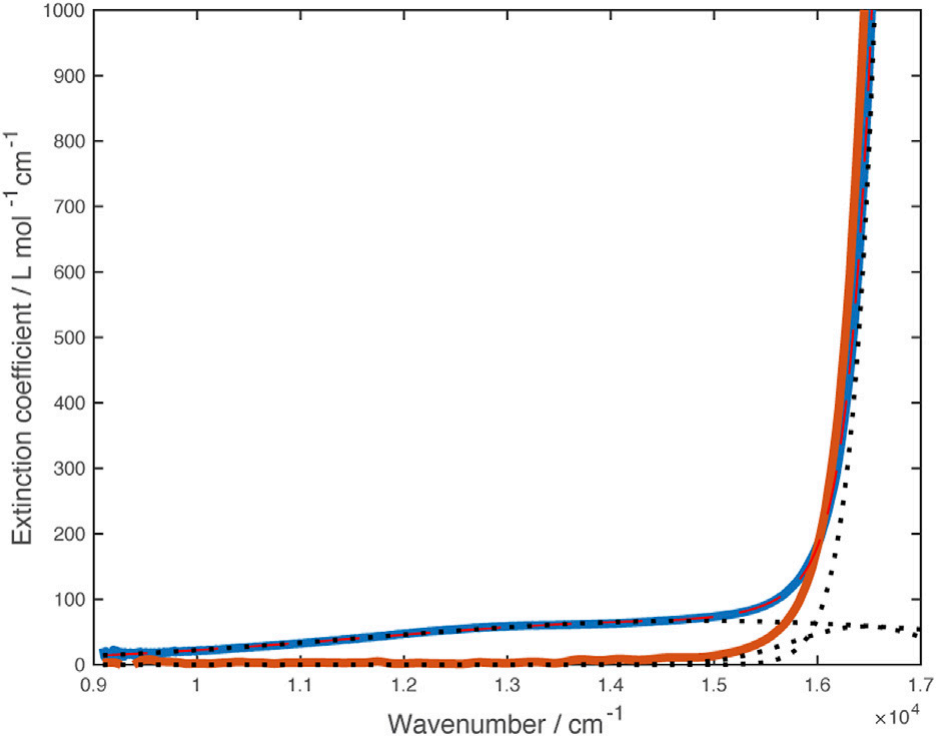
Vis-NIR spectra of [Ni(dipyvd)_2_]^2+^(PF_6_)_2_ (blue) and [Zn (dipyvd)_2_]^2+^(PF_6_)_2_ (orange) in acetonitrile. Dotted lines illustrate component gaussian curves used to model this region of the Ni spectrum.

Some features of [Ni(dipyvd)_2_]^2+^(PF_6_^−^)_2_were reported previously (Richardson et al., 2010); the molecule shows strong ferromagnetic coupling between spins, resulting in an S = 2 ground state. Though exchange parameters were strongly correlated, fitting of magnetic data to a simple Heisenberg model suggested metal-radical exchange parameters between +220 cm^–1^ and +350 cm^–1^, and radical-radical exchange between +510 cm^–1^ and +100 cm^–1^ ( $\hat{H}=-JS_{1}\cdot S_{2}$ ). The electronic spectrum shows a prominent band with a maximum at 585 nm almost identical to that observed for [Zn (dipyvd)_2_]^2+^(PF_6_^−^)_2_ and thus is almost certainly an intraligand transition. Examination of more concentrated solutions showed a weak shoulder on the low energy side of the band at 585 nm that is not present in the zinc analog (Figure 5). Fitting this region of the spectrum to gaussian curves gave an absorption centered at 14,850 cm^–1^ and a weaker band mostly obscured by the ligand transition at 16,450 cm^–1^.

**FIGURE 6:**
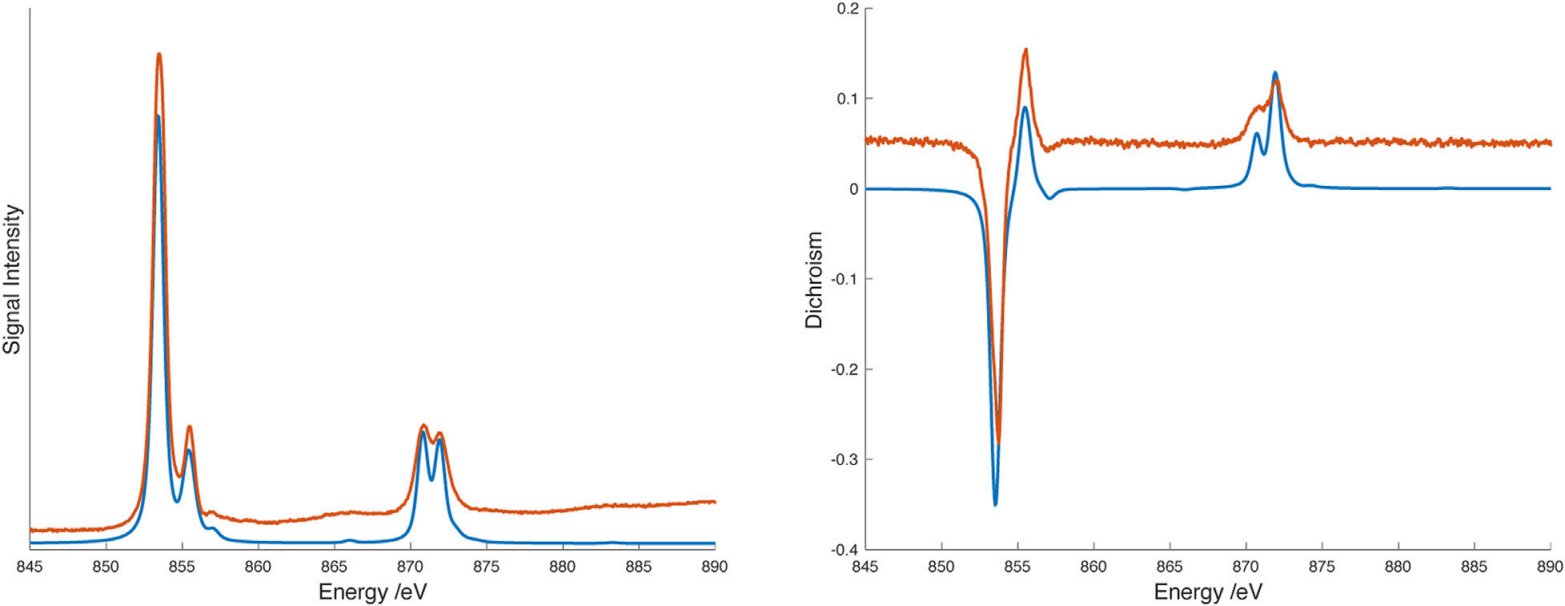
*L*_*2,3*_ edge X-ray absorption (XAS) spectrum (left) and X-ray magnetic circular dichroism (XMCD) spectrum (right) spectra of [Ni(dipyvd)_2_]^2+^(PF_6_)_2_ at 2.3K/6T. Experimental data are shown in orange, spectral simulations are shown in blue. Experimental data are offset along the vertical axis for clarity.

Though the lower energy band may correspond to the transition to the ^*3*^ *T*_*2*_(F) state (corresponding to 10*Dq*) expected for Ni^2+^, we are not confident of this assignment because of the breadth of the line and the overlapping ligand band(s). Consequently we turned to X-ray spectroscopy to further probe the transition metal electronic structure. X-ray absorption spectra (XAS) and X-ray magnetic circular dichroism (XMCD) were recorded on the nickel *L*_2,3_ edge at 2K and 25 K in a 6T field. The data were analyzed using semiempirical (crystal field multiplet) simulations and application of the relevant sum rules (Thole et al., 1992; Carra et al., 1993). The sum rule calculations gave *L*_*z*_ = 0.12µ_B_ *S*_*eff*_ = 0.37µ_B_. The discrepancy between expected magnetic moment and the values extracted from XMCD data has been previously noted; nevertheless, the residual orbital momentum is close to what is expected for Ni^2+^ ions (Arrio et al., 1996). Simulation of XAS/XMCD spectra is challenging because of the number of parameters that can be changed. As noted by Kowalska et al. for iron spectra (Kowalska et al., 2017), some parameters have only marginal impact on the shape of the spectrum, others may be strongly correlated. We began with parameters previously reported for octahedral nickel amine systems (van Elp et al., 1994) using an 80% reduction of the Slater-Condon parameters (corresponding to a Racah B parameter of about 800 cm^–1^), spin orbit coupling values of ζ(3d) = 0.083 for the initial Hamiltonian, and ζ(3d) = 0.10, ζ(2p) = 11.51 for the final Hamiltonian and a value of 5 eV for the Hubbard potential, *U*. With these parameters, the value of 10*Dq* suggested by the fit to the visible spectrum (14,850 cm^–1^ or 1.84 eV) matched the overall shape of the experimental X-ray spectra though there were small discrepancies in the locations of the spectral features. Improvements to the fit were obtained by including hybridization between the ligands and metal *d* orbitals (corresponding to some amount of sigma bonding) while lowering the value of 10*Dq*. The best match was obtained with 10*Dq* = 1.6 eV (12,900 cm^–1^), Δ(3*d*, L) = 5 eV, *V* (*e*_*.g.,*_) = 2.4 eV, *V* (*t*_*2g*_) = 1.2 eV. Spectra and simulations are shown in Figure 6. Simulation strategy and full simulation parameters are given in the Supporting Material.

**FIGURE 7:**
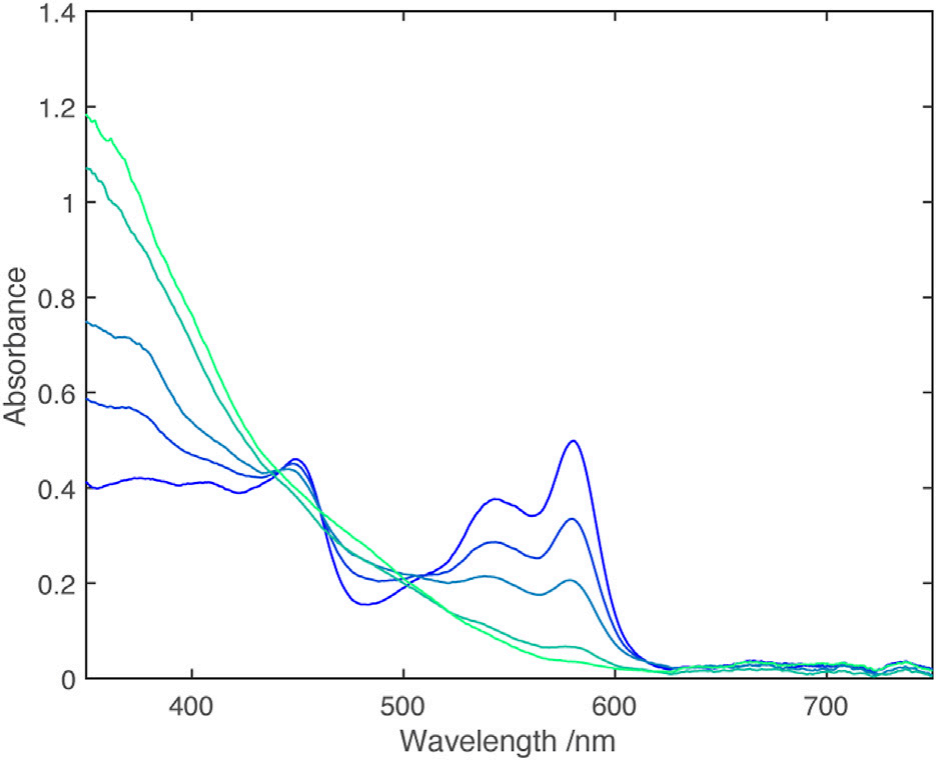
UV-vis spectra from electrochemical reduction of [Ni(dipyvd)_2_]^2+^ in acetonitrile. The working electrode potential ranges from 0.0 V vs. Fc/Fc^+^ (dark blue) to −0.4 V vs. Fc/Fc^+^ (light blue) in 0.1 V steps.

Cyclic voltammetry reveals two closely spaced one electron reductions (Table 2) and spectra obtained through spectroelectrochemistry are shown in Figure 7.

**FIGURE 8:**
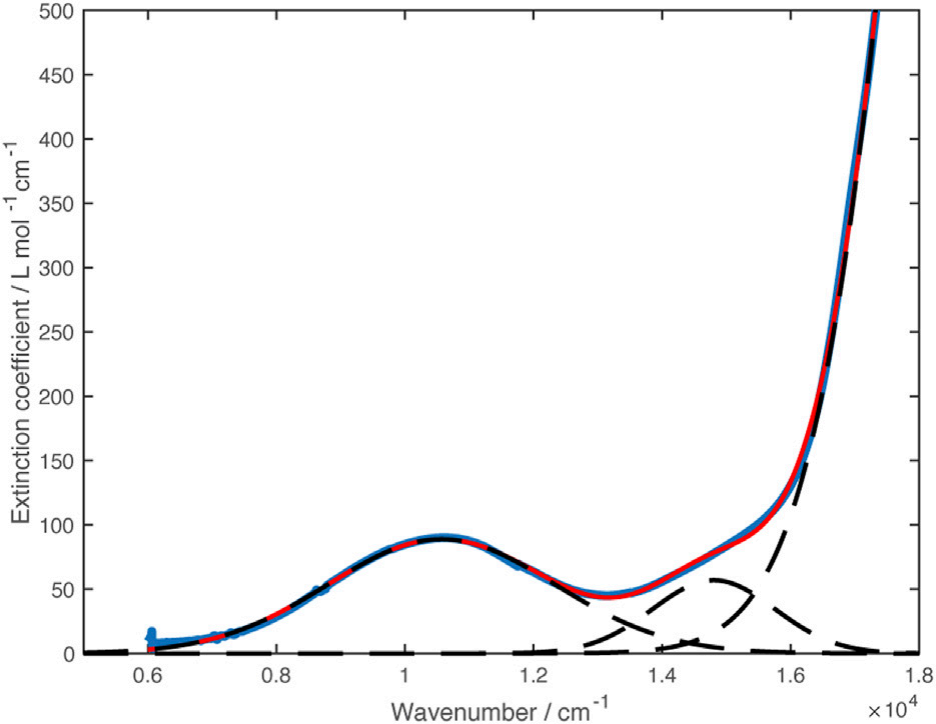
VIS-NIR spectrum of Ni(dipyvd)_2_ (blue curve). Dashed lines show the Gaussian curves used to model the spectrum. The sum of the gaussian curves is plotted in red.

Analogously to Zn, the fully reduced Ni complex could also be isolated as an orange-red solid by combination of the leucoverdazyl and nickel triflate in methanol, followed by deprotonation of the resulting species with triethylamine and precipitation with water. Hexagonal single crystals of Ni(dipyvd)_2_ were grown from dichloromethane/heptane. X-ray diffraction measurements indicated that the material was isostructural with the Zn analog; though the data quality is relatively low, the molecule, and in particular the ligand, shows the same structural features (non-planarity, bond length alternation noted for the zinc analog) observed for Zn. Unit cell dimensions are given in Table 1. Magnetic susceptibility measurements on Ni(dipyvd)_2_ gave *χT* = 1.18 over the range 50–300 K consistent with an S = 1 species with *g* = 2.2, (Supporting Material) typical for an octahedral *d* ^*8*^ Ni^2+^ ion (Titiš and Boča, 2010). UV-vis-NIR spectroscopy of Ni(dipyvd)_2_ in acetonitrile at high concentrations revealed two weak *d-d* transitions in the vis-NIR range. One of these was clearly resolved, the second had significant overlap with the intraligand transition. The spectrum was modeled using gaussian curves to give transition frequencies of 10,600 cm^–1^ and 14,800 cm^–1^ (Figure 8). The lower energy of these bands is almost certainly the expected ^*3*^ *A*_*2*_ to ^*3*^ *T*_*2*_ transition for a *d* ^*8*^ octahedral ion. The identity of the latter is more ambiguous and will be addressed in the discussion section.

**FIGURE 9:**
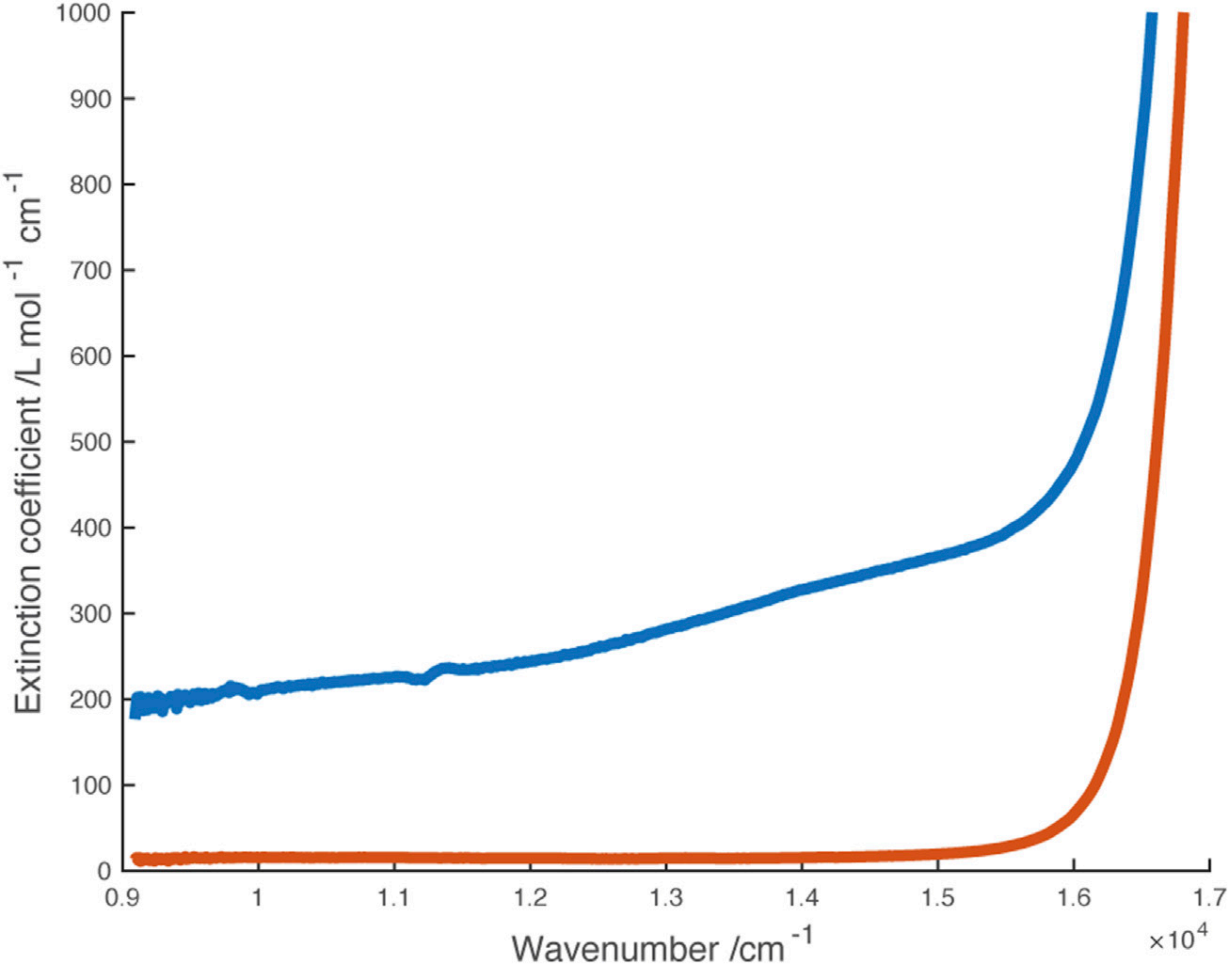
Vis-NIR spectra of monocations [Ni(dipyvd)_2_]^+^ (blue) and [Zn (dipyvd)_2_]^+^ (orange) generated in solution in 50/50 acetonitrile/dichloromethane.

Solutions containing the monocation were obtained through partial oxidation of Ni(dipyvd)_2_ with AgPF_6_, partial reduction of [Ni(dipyvd)_2_](PF_6_)_2_ with cobaltocene or combination of equimolar amounts of Ni(dipyvd)2 and [Ni(dipyvd)_2_](PF_6_)_2_. Electronic spectra of these solutions showed absorption tailing into the near IR that may be due to a combination of *d-d* transitions and intervalence charge transfer; while weak compared to the intraligand bands, this absorption was significantly stronger than the *d-d* transitions observed for the dication and neutral species. With no clear maxima however, no attempt was made to model the spectrum. It is notable that no corresponding absorption was observed in solutions of the zinc monocationic species (Figure 9).

**FIGURE 10:**
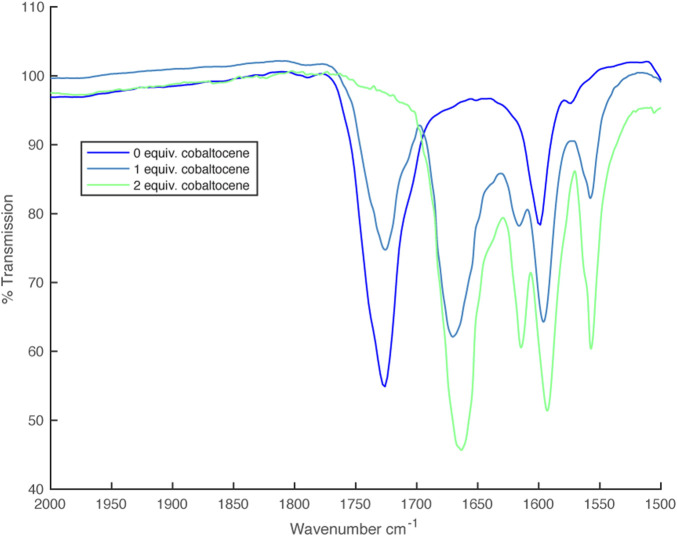
IR spectra from partial reduction of [Ni(dipyvd)_2_]^2+^ with cobaltocene.

These same partly oxidized/reduced solutions gave IR spectra with 2 C=O stretches at 1,668 cm^–1^ and 1724 cm^–1^ corresponding to the anionic and radical ligands respectively (Figure 10).

Though we attempted to grow single crystals of the monocation, we could not find conditions where either Ni(dipyd)_2_ or [Ni(dipyvd)_2_](PF_6_)_2_ did not precipitate preferentially, driving the equilibrium toward a mixture of dication and neutral species.

### 3.3 Computational studies

**FIGURE 11:**
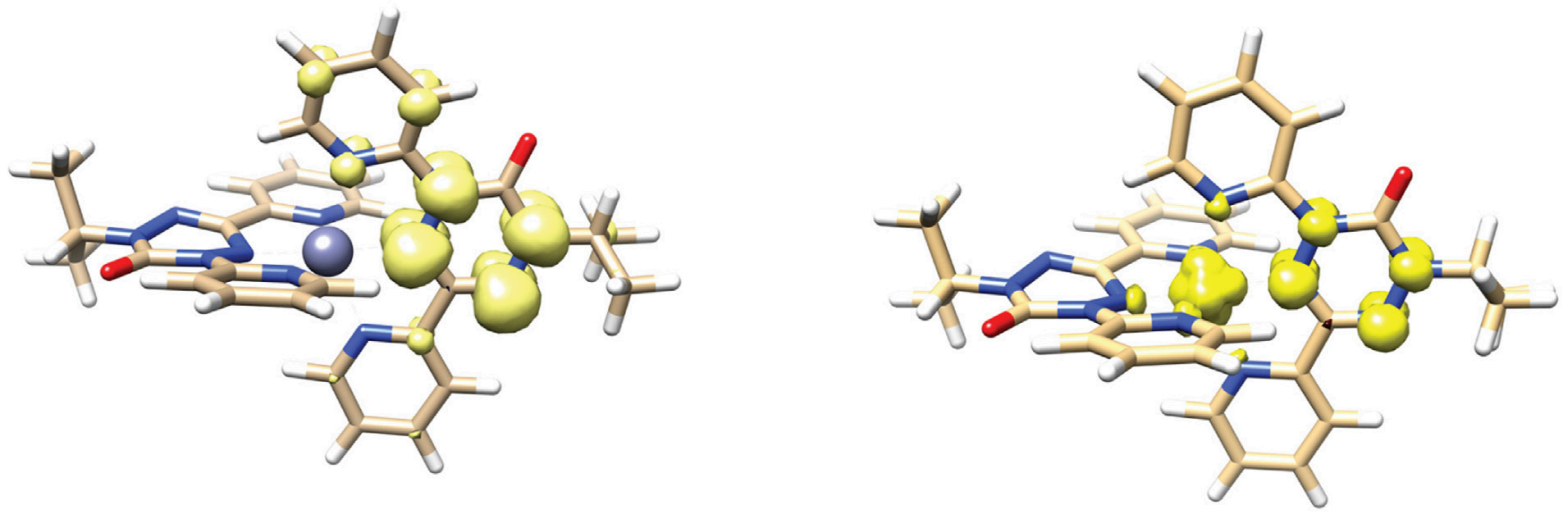
DFT calculated geometry and spin density distribution of [Zn (dipyvd)_2_]^+^ (left) and [Ni(dipyvd)_2_]^+^ (right).

DFT geometry optimizations using the PBEh-3c functional (Grimme et al., 2015; Janetzki et al., 2021) reproduce the key structural features of both dications and neutral species. In particular the verdazyl ligands are calculated to be planar, while the anionic ligands have the boat shaped geometry previously observed in the species [Co(dipyvd)_2_]^+^PF_6_. For the mixed valence cations [Zn (dipyvd)_2_]^+^ and [Ni(dipyvd)_2_]^+^, the PBEh-3c functional predicts localized structures consistent with spectroscopic evidence; one of the ligands is planar while the other has a distinct boat shape and the spin density is localized on one of the ligands (Figure 11 and Supporting Material). It is noteworthy that the more commonly used B3LYP functional predicts symmetric, delocalized structures for the intermediate cations which is inconsistent with infrared spectral data. Using the PBEh-3c derived geometries we computed magnetic exchange using the broken symmetry approach and Yamaguchi formula, and UV-vis spectra using time dependent DFT calculations with the CAMB3LYP functional suggested by Janetski.

The broken symmetry calculations gave values consistent with experimental data for the two dications. In particular, for [Zn (dipyvd)_2_]^2+^ *J*_*vv*_ was essentially zero (+0.37 cm^–1^) which is consistent with our data and earlier computational results. (Roseiro et al., 2023). For [Ni(dipyvd)_2_]^2+^ two broken symmetry calculations (Bsym (2,2) and Bsym (3,1)) were used (Ruiz et al., 2003) to give *J*_*Ni-verd*_ = 356 cm^–1^ and *J*_*vv*_ = 124 cm^–1^ (best fit experimental values were *J*_*Ni-verd*_ = 300 cm^–1^ and *J*_*vv*_ = 160 cm^–1^ (Richardson et al., 2010)). Calculations on the cation [Ni(dipyvd^–^)(dipyvd^•^)]^+^ also gave *J*_*Ni-verd*_ = 356 cm^–1^, however we have not been able to experimentally confirm this value because we have not been able to isolate this species.

The TD-DFT calculations reproduced the main spectral features of the Zn and Ni species in the visible-NIR ranges. In particular transitions for the Ni species at 12,682 cm^–1^, 13,145 cm^–1^ and 13,305 cm^–1^ for Ni(dipyvd)_2_, [Ni(dipyvd^–^)(dipyvd^•^)]^+^, [Ni(dipyvd^•^)_2_]^2+^ respectively were identified as predominantly *d-d* in nature, supporting our experimental assignments (though the transition energies are slightly higher than the experimental values). In addition, the lowest energy transition for [Ni(dipyvd^–^)(dipyvd^•^)]^+^ was determined to be an intervalence charge transfer band between the ligands at 11,271 cm^–1^. Corresponding calculations on [Zn (dipyvd^–^)(dipyvd^•^)]^+^ predicted an IVCT band at 12,384 cm^–1^ though we have found no experimental evidence for it. Listings of frequencies and transition probabilities are provided in the Supporting Material.

## 4 Discussion

### 4.1 Metal-ligand interaction from the metal perspective

For Ni^2+^ the ligand influence will be reflected in the *d* orbitals; the standard description of *d*-orbital splitting in an octahedral field is an obvious starting point. With 8 *d* electrons, octahedral Ni^2+^ has a filled *t*_*2g*_ orbital set and half-filled *e*_*g,*_ orbital set resulting in only one possible (*S* = 1) ground state independent of the ligand field. The weak tetragonal distortion resulting from the chelating nature of the ligand would be expected to lift the degeneracy of both the *t*_*2g*_ and, *e*_*g,*_ orbital set but we would not expect a large difference in the analysis as a result. Nickel (II) has limited redox activity, consequently the analysis is not complicated by phenomena such as spin crossover and valence tautomerism. The octahedral ligand-field splitting 10*Dq* and the Racah Parameters *B* and *C*, provide a useful way to compare the influence of ligand on the metal ion, however their determination from UV-vis spectra requires the correct assignment of the *d-d* transitions. For an octahedral *d* ^8^ system three spin allowed (but Laporte forbidden) *d-d* transitions are expected from the ground state (^*3*^ *A*_*2*_) to the ^*3*^ *T*_*2*_, ^3^ *T*_*1*_(F) and ^*3*^ *T*_*1*_(P) states. There is also a spin forbidden transition to the ^*1*^ *E* state that can gain intensity through spin orbit coupling (Hart et al., 1983). For Ni(dipyvd)_2_ we initially assigned the two low intensity bands to the ^*3*^ *T*_*2*_ and ^*3*^ *T*_*1*_(F) transitions respectively; while this gives an appropriate value for 10*Dq*, the resulting value of *B* is very low. Hart and co-workers have observed that for Ni^2+^ coordinated solely by N, O or F ligands, the energies of the ^*3*^ *T*_*1*_(F) and ^*1*^ *E* transitions are roughly correlated with the ^3^ *T*_*2*_ transition and *B* is empirically related to 10 *Dq* (in cm^–1^) by the expression:

$$B=1120-0.022\left(10Dq\right)$$

The value of *B* derived from our initial assignment deviates significantly from this relationship. Rather than assume something very special about the **dipvd** ^**–**^ ligand, we reexamined our assignment. Based on the wealth of data presented by Hart, with the ^3^ *T*_2_ transition at 10,600 cm^–1^, we would expect to find the ^3^ *T*_1_(F) transition near 17,000 cm^–1^ (where it would be obscured by the ligand pi-pi* transition). The absorption at 14,800 cm^–1^ is more likely the spin forbidden ^*1*^ *E* transition which is consistent with its lower intensity based on the fitting procedure. Under these circumstances, the values of B and C cannot be determined independently based on spectroscopic information; use of Hart’s empirical relationships do provide values for B and C but these do not provide any further information than the value of 10 *Dq* since they are derived from it.

**FIGURE 12:**
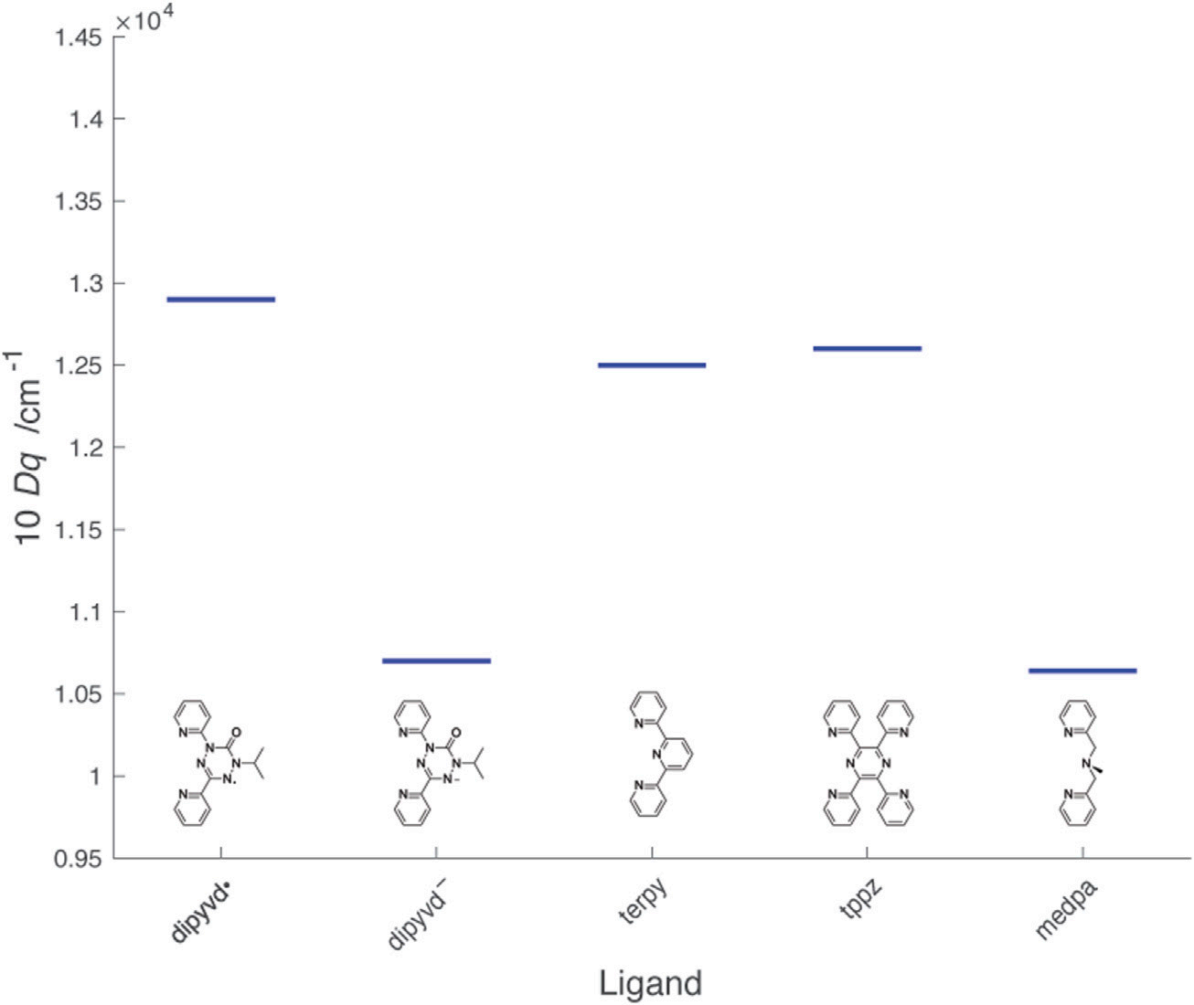
Values of 10*Dq* for Ni^2+^L_2_ complexes of dipyvd and structurally related ligands. Values for the other ligands were determined from λ_max_ from literature reports: terpy (Prasad and Scaife, 1977), tppz (Haghighi et al., 2013), mdepa (Hazell et al., 2000).

Determining the value of 10*Dq* for the radical ligand is more challenging because the relevant *d-d* transitions are not clearly resolved; furthermore, the normal spin selection rules are complicated by the presence of paramagnetic ligands. X-ray spectroscopy provides an alternate way to estimate 10*Dq* that provides values consistent with optical spectroscopy; the two techniques together demonstrate that upon reduction from the radical to anionic ligand, 10*Dq* decreases significantly. In the standard ligand field approach, smaller values of 10*Dq* result from less sigma donation from the ligand to the metal ion, and/or less pi donation (back bonding) from the metal ion to the ligand. These concepts are illustrated by 10*Dq* for the Ni^2+^ complexes of structurally related ligands shown in Figure 12.

The heterocyclic ligands terpyridine (terpy) and 2,3,5,6-tetra(2′-pyridyl)pyrazine (tppz) are structurally similar to dipyvd• though with empty (rather than half full) pi* orbitals and show similar values of 10*Dq* The slight increase in 10*Dq* in the order terpy < tppz < dipyvd• is consistent with an increase in electron deficiency of the central ring associated with more nitrogen atoms. Upon reduction, the tetrazine ring of dipyvd can no longer act as a pi acceptor and though the now anionic ligand would be expected to be significantly more basic, the reduced 10*Dq* indicates that this is not sufficient to compensate for the loss of pi acceptor character. Comparison of dipyvd^–^ with the tridentate ligand bis(pyridylmethyl)methylamine (medpa) further supports this analysis. Like dipyvd^–^, medpa has two coordinating pyridine rings and the central amine nitrogen has no available empty pi orbitals; the values of 10*Dq* for [Ni(medpa)_2_]^2+^ and Ni(dipyvd)_2_ are quite similar. The large change in ligand field between dipyvd• and dipyvd^–^as a result of the transfer of a single electron undoubtedly plays a role in the unusual properties of other metal compounds with this ligand system.

### 4.2 Metal-ligand interaction from the ligand perspective

Structural studies on other verdazyl radicals indicate that the tetrazine ring is planar and essentially symmetrical about the C1-C3 axis; a geometry that is typically maintained in coordination. The verdazyl nitrogen lone pair is weakly basic, as is typical for tetrazines; there are no examples of coordinated verdazyls without an additional chelating group to hold the verdazyl in place. Even then (with the notable exception of the current ligand) the metal verdazyl distance is often the longest of any metal ligand distances within the compound. DFT calculations, supported by ESR spectra (Neugebauer et al., 1988; Paré et al., 2005), give the verdazyl SOMO as an antibonding pi orbital that is mostly located on the four tetrazine nitrogen atoms, though for the dipyvd ligand there is some spin density on the 1-pyridyl ring (Richardson et al., 2010). Because it is half filled, this orbital has the potential to act as both a pi donor and pi acceptor (Brook and Abeyta, 2002). The acceptor character of the SOMO is illustrated by the electronic spectra. The UV-vis spectrum of 3-aryl substituted 6-oxoverdazyls typically consists of two bands in the near UV/visible range. Experiments with different aryl groups established that charge transfer between the aryl substituent and the verdazyl SOMO makes a significant contribution to the low energy band (Chemistruck et al., 2009). Furthermore, the dihedral angle between the aromatic ring and verdazyl (and thus the level of conjugation) plays an important role; in the o-hydroxyverdazyl the lowest energy band shows strong vibronic structure and a slight red shift resulting from a geometry restricted by an internal hydrogen bond. Conversely in the o-methoxy phenyl verdazyl, steric hindrance prevents conjugation between the two rings and the lowest energy band is strongly blue shifted (Chemistruck et al., 2009).

Addition of one electron gives a cyclic 8π*e* system that, if it maintained planar geometry, would be formally antiaromatic. In fact, the ring distorts from planarity and adopts a bond length alternation to avoid full antiaromatic conjugation. This is seen in the crystal structure of the leucoverdazyl dipyvdH as well as in the structures of Ni(dipyvd)_2_ and Zn (dipyvd)_2_ and other coordinated anionic verdazyls (Fleming et al., 2020). Calculations also support this structure for the ligand. The lack of a low lying empty pi orbital is consistent with the relatively low value of 10 Dq reported above.

With a dipositive charge but filled *d* orbitals, Zn^2+^ provides a solid foundation for understanding the influence of divalent metal coordination upon the ligand without interference from *d* orbital overlap. The most obvious changes are observed in the UV-vis spectrum, where the ligand itself is an orange-yellow color, but the Zn^2+^ and Ni^2+^ coordination compounds are magenta and red respectively. These striking colors result from a band with a maximum at 585 nm that shows almost identical vibronic structure in both the Ni^2+^ and Zn^2+^ species.

Previous examples of coordination of zinc to verdazyls resulted in red shifts of the lowest energy band of about 0.5 eV accompanied by better resolved vibronic structure and a slight increase in extinction coefficient (Brook et al., 2000; Barclay et al., 2003; Anderson et al., 2011). The Zn complex of dipyvd shows these same effects but magnified. The low energy band decreases in energy by ∼0.75 eV accompanied by a more pronounced increase in intensity and vibronic structure. These changes are likely due to the influence of the positively charged metal ion lowering the energy of radical SOMO while the geometry restrictions due to chelation increase the degree of conjugation and reduce the vibrational degrees of freedom. The difference between dipyvd and other verdazyls results from the significantly shorter metal-verdazyl distance (2.0 Å vs. 2.3 Å).

Further supporting this analysis, the redox potentials of the [Zn (dipyvd)_2_]^n+^ system are much higher (by ∼ 1 V) than reported for uncoordinated 6-oxo verdazyls, and significantly higher (by ∼0.6 V) than that for the other reported Zn verdazyl systems (Anderson et al., 2011). Similar shifts were observed in the case of ligand redox chemistry in [Zn (terpy)_2_]^n+^ (Elgrishi et al., 2014), though for the verdazyl the potentials are significantly higher as a result of the electron deficient nature of the verdazyl ring. Upon reduction the low energy band in the UV-vis spectrum is completely lost, consistent with occupation of the SOMO. Stabilization of this orbital by the metal ion is also consistent with the reduction in pKa of the ligand upon coordination, though it is likely that the anionic ligand retains significant antiaromatic structure.

The similarity of the spectral (aside from *d-d* transitions discussed above) and electrochemical properties of [Ni(dipyvd)_2_]^n+^ and [Zn (dipyvd)_2_]^n+^ suggest that the influence of the metal on the ligand electronic structure is largely the same with the partly occupied *d* orbitals of Ni^2+^ playing a minimal role.

### 4.3 Ligand-ligand interaction

In a system such as [M(dipyvd)_2_]^n+^, a third consideration is the strength of the ligand-ligand interaction, and in particular the extent to which the metal ion influences this interaction. We can assess this interaction in several different ways. In the context of the dication, the radical-radical interaction is most obviously expressed in terms of magnetic exchange. Recent computational studies indicate that [Zn (dipyvd)_2_]^2+^ has a triplet ground state, but the first excited singlet is only slightly higher in energy (Roseiro et al., 2023). The current data are consistent with these results with |*J*| ≤ 3 cm^–1^. This exchange interaction is very weak compared with many other dipyvd and verdazyl coordination compounds, however it is consistent with other zinc-verdazyl compounds and also other zinc-radical systems. The stronger radical-radical exchange observed with verdazyl complexes with other diamagnetic metals, illustrates the role metal orbitals play in mediating radical-radical interaction. Though its value is hard to pin down precisely, the larger radical-radical exchange observed in [Ni(dipyvd)_2_]^2+^ speaks to the slightly higher energy *d*-orbitals in Ni^2+^ and their role in mediating magnetic exchange. This also illustrates that in cases like that of the nickel complex, where assessing the role of radical-radical vs radical metal exchange is challenging, the practice of using the zinc analog to estimate the radical-radical interactions is probably only valid when considering through space interaction.

Interligand interaction should also be apparent in the mixed-valence monocations which can be classified using the Robin-Day scheme. All evidence suggests that [Zn (dipyvd)_2_]^+^ is a class I system (i.e., completely localized with minimal interaction). The UV-vis spectrum is essentially an average of the spectra of the dication and neutral species; the C=O region of the IR spectrum shows stretches for anionic and radical ligands unshifted from their positions in the neutral and dicationic species respectively, and the ESR spectrum shows hyperfine coupling with only one ligand. The corresponding nickel species [Ni(dipyvd)_2_]^+^ is a little more ambiguous. While the major features of the UV-vis and IR spectra parallel those of the zinc analog, a weak absorption tail into the NIR that is not apparent in either the dication or neutral species suggests some level of ligand-ligand interaction in the form of intervalence charge transfer. This is also hinted at by the slightly larger separation of successive ligand redox events. [Ni(dipyvd)_2_]^+^ is probably best classified as a class II system (localized but with some interaction) though it is clearly right on the border between class I and class II. DFT calculations support a localized structure for both systems, though not with the commonly used B3LYP functional, which illustrates the computational challenges associated with mixed valence systems.

Both the nickel and zinc systems contrast drastically with the corresponding cobalt and iron analogs. In the latter species, the UV-vis spectra are dominated by (Ligand-Metal or Metal-Ligand) charge transfer bands rather than the simple ligand or metal localized transitions observed in the Ni and Zn species. Similarly for the iron and cobalt species, the redox events are more widely spaced (∼0.4–∼0.8 V vs. 0.15 V) and cannot be simply attributed to ligand or metal based processes. These observations point to a greater degree of covalent interaction in the iron and cobalt species (at least in the pi manifold). Since the geometry of the coordination environment is very similar, the difference in behavior is likely due to the decrease in *d*-orbital energy relative to the ligand SOMO across the period. For iron and cobalt, the similar *d*-orbital energies provide for increased delocalization (iron) or facile electron transfer between metal and ligand (cobalt). With nickel, the lower energy *d*-orbitals provide less covalency but still can mediate some level of interaction between ligands as indicated by the weak intervalence charge transfer band in the [Ni(dipyvd)_2_]^+^, and interligand magnetic exchange in [Ni(dipyvd)_2_]^2+^. By the time we get to zinc, however even that weak interaction has disappeared and the two ligands are essentially independent. In this context, study of the manganese and copper analogs of these species, should be informative. The manganese *d*-orbitals should be higher energy than those of iron, but it is not immediately clear if this will lead to greater or less covalency. The copper coordination may be complicated by the valence tautomeric formation of copper(I) species. These will be subjects of further study in our laboratory.

## 5 Conclusion

Though the zinc and nickel coordination compounds of the dipyridylverdazyl ligand do not show spin crossover or valence tautomerism, their straightforward electronic structure helps discern how the metal-ligand interaction modifies the electronic structure of both metal and ligand. In particular, coordination of a metal ion to the dipyvd ligand shifts the ligand redox events into an easily accessible range, and at the same time redox events on the ligand change the nature of the interaction with metal *d*-orbitals, switching between a sigma/pi donor and pi acceptor. Comparison of the dipyvd compounds with other verdazyl coordination compounds also illustrates how changes in ligand arrangement (re: the location of the verdazyl in the middle rather than at the end of the tridentate unit) can have a significant impact on the nature and strength of these interactions. Furthermore the weak interligand interactions in these species emphasize the critical role played by metal *d* orbital energies. We expect these results to contribute to our understanding of the other transition metal compounds with this ligand, as well as to the design of other valence tautomeric systems.

## Data Availability

The datasets presented in this study can be found in online repositories. The names of the repository/repositories and accession number(s) can be found below: https://www.ccdc.cam.ac.uk/solutions/csd-system/components/csd/, 2267079 https://www.ccdc.cam.ac.uk/solutions/csd-system/components/csd/, 2267621 https://www.ccdc.cam.ac.uk/solutions/csd-system/components/csd/, 753408 https://www.ccdc.cam.ac.uk/solutions/csd-system/components/csd/, 2267243 https://www.ccdc.cam.ac.uk/solutions/csd-system/components/csd/, 2267027.
