# Supplementary material for: Metal-ligand interactions in a redox active ligand system. Electrochemistry and spectroscopy of [M(dipyvd)2]n+ (M=Zn, Ni, n=0, 1, 2)
Source: Front Chem. 2023 Nov 15;11:1295289. doi: 10.3389/fchem.2023.1295289 (PMC10684738; doi:10.3389/fchem.2023.1295289)
Supplement: Supplementary file 3 [file DataSheet5.docx]

Supplementary Material

# Supplementary Figures and Tables

**Supplementary** **Figure 1**. ^1^H NMR of Zn(dipyvd)_2_

**Supplementary Figure 2.** ^13^C NMR of Zn(dipyvd)_2_

**Supplementary Figure 3.** ^1^H COSY NMR of Zn(dipyvd)_2_.

**Supplementary Figure 4.** ^1^H-^13^C HMQC NMR of Zn(dipyvd)_2_

_<IR> spectra_

**Supplementary Figure 5.** Magnetic data (*χT vs T*) for Ni(dipyvd)_2_ (light blue diamonds) and Zn(dipyvd)_2_(PF_6_)_2_ (dark blue triangles).

**Supplementary Figure 6.** Cyclic voltammogram for Zn(dipyvd)_2_(PF_6_)_2_. Electrolyte: 0.1M Bu4NPF6 in CH3CN. Scan rate 100 mV/s

**Supplementary Figure 7.** Cyclic voltammogram for Ni(dipyvd)_2_(PF_6_)_2_. Electrolyte 0.1M Bu4NPF6 in CH3CN. Scan rate 100 mV/s

**Supplementary Table 1. DFT Optimized xyz coordinates for Zn(dipyvd)_2_**

**Supplementary Table 2. DFT Optimized xyz coordinates for [Zn(dipyvd)_2_]^+^**

**Supplementary Table 3. DFT Optimized coordinates for [Zn(dipyvd)_2_]^2+^**

**Supplementary Table 4. DFT Optimized coordinates for Ni(dipyvd)_2_**

**Supplementary Table 5. DFT Optimized coordinates for [Ni(dipyvd)_2_]^+^**

**Supplementary Table 6. DFT Optimized coordinates for [Ni(dipyvd)_2_]^2+^**

**Supplementary Data: XAS/XMCD Simulation details**

**Supplementary Data: XAS/XMCD Application of sum rules to XMCD data (separate file)**

**Supplementary Figure 1.** ^1^H NMR of Zn(dipyvd)_2_ with peak assignments.

**Supplementary Figure 2.** ^13^C NMR of Zn(dipyvd)_2_

**Supplementary Figure 3.** ^1^H COSY NMR of Zn(dipyvd)_2_.

**Supplementary Figure 4.** ^1^H-^13^C HMQC NMR of Zn(dipyvd)_2_

**Supplementary Figure 5**: IR spectrum and peak list for Zn(dipyvd)_2_

| Wavenumber/cm^–1^ | %Transmittance | Wavenumber/cm^–1^ | %Transmittance |
| --- | --- | --- | --- |
| 660 | 75 | 1223 | 43 |
| 721 | 62 | 1258 | 62 |
| 751 | 73 | 1290 | 35 |
| 772 | 63 | 1312 | 59 |
| 794 | 75 | 1342 | 56 |
| 818 | 83 | 1385 | 44 |
| 908 | 85 | 1437 | 47 |
| 999 | 66 | 1463 | 31 |
| 1027 | 79 | 1474 | 48 |
| 1042 | 80 | 1558 | 47 |
| 1100 | 73 | 1590 | 41 |
| 1129 | 74 | 1614 | 63 |
| 1148 | 50 | 1661 | 36 |
| 1178 | 57 | 2928 | 83 |

**Supplementary Figure 6**: IR spectrum and peak list for Ni(dipyvd)_2_

| Wavenumber/cm^–1^ | %Transmittance | Wavenumber/cm^–1^ | %Transmittance |
| --- | --- | --- | --- |
| 654 | 57 | 1256 | 58 |
| 680 | 71 | 1286 | 47 |
| 718 | 53 | 1310 | 61 |
| 749 | 53 | 1346 | 52 |
| 768 | 52 | 1385 | 57 |
| 818 | 70 | 1439 | 52 |
| 1001 | 58 | 1459 | 45 |
| 1025 | 65 | 1474 | 57 |
| 1042 | 67 | 1556 | 54 |
| 1098 | 59 | 1590 | 50 |
| 1129 | 57 | 1614 | 62 |
| 1148 | 50 | 1659 | 48 |
| 1176 | 54 | 2117 | 90 |
| 1223 | 51 | 2963 | 76 |

**Supplementary Figure 7**: IR spectrum and peak list for [Zn(dipyvd)_2_]^2+^(PF_6_)_2_

| Wavenumber/cm^–1^ | %Transmittance | Wavenumber/cm^–1^ | %Transmittance |
| --- | --- | --- | --- |
| 779.1 | 82.8292 |  |  |
| 840.81 | 65.6627 |  |  |
| 1014.38 | 83.2776 |  |  |
| 1159.01 | 81.5241 |  |  |
| 1224.58 | 81.1448 |  |  |
| 1243.86 | 80.7501 |  |  |
| 1278.58 | 79.5958 |  |  |
| 1446.35 | 77.9903 |  |  |
| 1469.5 | 78.6023 |  |  |
| 1600.63 | 78.6794 |  |  |
| 1725.98 | 76.1588 |  |  |

**Supplementary Figure 8**: IR spectrum and peak list for [Ni(dipyvd)_2_]^2+^(PF_6_)_2_

| Wavenumber/cm^–1^ | %Transmittance | Wavenumber/cm^–1^ | %Transmittance |
| --- | --- | --- | --- |
| 557.33 | 41.6806 | 1245.79 | 75.74 |
| 630.61 | 71.6373 | 1276.65 | 72.3097 |
| 655.68 | 75.3542 | 1340.29 | 79.6508 |
| 709.68 | 79.6396 | 1373.07 | 77.5036 |
| 750.17 | 80.9387 | 1411.64 | 75.2205 |
| 777.17 | 73.0901 | 1438.64 | 68.8918 |
| 840.81 | 40.5943 | 1465.64 | 71.1604 |
| 1014.38 | 80.7391 | 1598.7 | 71.351 |
| 1049.09 | 81.9111 | 1725.98 | 61.9545 |
| 1159.01 | 77.5156 | 3646.75 | 69.216 |
| 1222.65 | 74.9545 |  |  |

**Supplementary Figure 9.** Magnetic data (*χT vs T*) for Ni(dipyvd)_2_ (light blue diamonds) and Zn(dipyvd)_2_(PF_6_)_2_ (dark blue triangles).

**Supplementary Figure 10**: X-band EPR spectrum of [Zn(dipyvd)_2_](PF_6_)_2_ in acetonitrile at 298K

**Supplementary Figure 6.** Cyclic voltammogram for Zn(dipyvd)_2_(PF_6_)_2_. Electrolyte: 0.1M Bu4NPF6 in CH3CN. Scan rate 100 mV/s

**Supplementary Figure 7.** Cyclic voltammogram for Ni(dipyvd)_2_(PF_6_)_2_. Electrolyte 0.1M Bu4NPF6 in CH3CN. Scan rate 100 mV/s

**Supplementary Table 1. DFT Optimized xyz coordinates for Zn(dipyvd)_2_**

Zn 8.18623199704247 8.18631983651944 3.19684998858864

O 11.68242082371924 4.62437445768878 1.67187351058059

N 10.01497443457611 7.30517880950875 3.19135233042237

N 10.14809974308166 6.07929058017474 2.54066238467375

N 12.28754847684985 5.96078841140896 3.38679986852100

N 11.96020522373839 6.84257866389305 4.39542057476182

N 9.36636182363194 9.18670495949128 4.90035685426989

N 7.98158403146834 6.46028897257879 1.93314120123556

C 11.41247423322656 5.48562764412125 2.47230977302557

C 10.87498289332333 7.49147420226498 4.18188481429764

C 10.53987292864226 8.60470480459683 5.09644917458698

C 11.41007971128847 9.02310041227305 6.08901375388951

H 12.35942566255627 8.52543954344565 6.20689535163414

C 11.02650445722990 10.06902189900517 6.89725337588497

H 11.67987618872051 10.41584831240220 7.68507398936590

C 9.80137790866757 10.67537371112384 6.68659110791685

H 9.46811444646487 11.50099047174175 7.29640325561826

C 9.00440842809197 10.19815972333875 5.66927258967144

H 8.03641059399793 10.63800371174395 5.46381019815646

C 6.83763273182552 6.13429472947912 1.34086855295594

H 6.03336267876780 6.85005323069200 1.45488195420236

C 6.66829552523708 4.97698315076086 0.63514123660063

H 5.72547846237104 4.74861797792634 0.16462462008330

C 7.75805941718691 4.11759087722779 0.55985727429129

H 7.67862412460130 3.18295320063446 0.02265938301345

C 8.94447362574312 4.43266462310078 1.16286062376915

H 9.78224006951235 3.76534317221693 1.10007880372385

C 9.03348210157052 5.64744365548097 1.86316432250871

C 13.64238530520969 5.43662635601865 3.46739645851488

H 13.77966132959833 4.82004118030047 2.58281993580822

C 14.65995521298326 6.56452452462451 3.42999829276305

H 14.53300385232502 7.18025181537791 2.54175939053274

H 15.66625520651825 6.15067039882090 3.40823346761905

H 14.57892452030953 7.20730318337158 4.30355833791128

C 13.81369246115529 4.55929459179838 4.69751391095295

H 13.65344761769944 5.12583948546891 5.61230415221456

H 14.82170092361511 4.14974080976234 4.72649530082719

H 13.11646072667495 3.72379249507412 4.68468542849357

O 4.68980144665012 11.74803700246924 1.67196456129849

N 6.35750840062399 9.06747354225000 3.19149564124390

N 6.22433244131561 10.29338827409264 2.54085362474839

N 4.08482471228295 10.41161396439843 3.38692392546118

N 4.41218326243531 9.52980162645515 4.39551172912364

N 7.00625316668126 7.18592731896065 4.90040669789061

N 8.39081324882523 9.91238726578378 1.93318080602109

C 4.95996537942565 10.88710248531483 2.47267056779238

C 5.49750972428934 8.88107652446995 4.18201575181971

C 5.83271995401440 7.76786009056862 5.09655351010953

C 4.96259760863446 7.34944056627586 6.08918057334346

H 4.01323776297914 7.84706259125971 6.20711580647077

C 5.34628012216404 6.30356337680043 6.89742597896876

H 4.69297831063715 5.95672525817317 7.68529935189755

C 6.57142775256370 5.69727645643279 6.68670513808051

H 6.90477407246944 4.87169136167187 7.29651457256872

C 7.36830956405790 6.17451276539563 5.66932779431935

H 8.33632263865106 5.73472282331797 5.46382274789800

C 9.53472936782616 10.23838517000639 1.34083999209767

H 10.33900803326753 9.52262970613152 1.45481076435498

C 9.70401715017985 11.39568502300512 0.63508259068647

H 10.64680594187484 11.62404945148979 0.16450885034498

C 8.61424129823530 12.25506246253758 0.55984023975996

H 8.69363409639866 13.18968730816341 0.02261377057684

C 7.42786286041845 11.93999168308586 1.16291873748763

H 6.59008703844568 12.60730200116501 1.10016116645403

C 7.33890202753735 10.72522673593249 1.86325632289229

C 2.72983008910011 10.93540297970982 3.46729137660090

H 2.59257550310726 11.55205885946993 2.58275986196430

C 1.71256381762349 9.80724423732244 3.42953215822594

H 1.83987318151151 9.19167402833786 2.54123534553657

H 0.70616086272386 10.22083984440644 3.40760379748171

H 1.79356963440967 9.16436647903497 4.30302080300053

C 2.55802974335221 11.81253361849314 4.69748527453268

H 2.71823647740776 11.24591224348945 5.61223502168564

H 1.54990427247422 12.22180960432533 4.72631028228651

H 3.25503913618223 12.64822668434354 4.68490932108639

**Supplementary Table 2. TD-DFT calculated electronic spectra for Zn(dipyvd)_2_**

-----------------------------------------------------------------------------

ABSORPTION SPECTRUM VIA TRANSITION ELECTRIC DIPOLE MOMENTS

-----------------------------------------------------------------------------

State Energy Wavelength fosc T2 TX TY TZ

(cm-1) (nm) (au**2) (au) (au) (au)

-----------------------------------------------------------------------------

1 25412.1 393.5 0.101552481 1.31560 -0.68525 0.91980 -0.00000

2 25516.7 391.9 0.011226358 0.14484 0.00010 -0.00034 -0.38058

3 30774.5 324.9 0.050785037 0.54327 -0.68791 -0.26468 -0.00015

4 30797.1 324.7 0.031279439 0.33437 0.00018 0.00014 -0.57825

5 32493.2 307.8 0.001418973 0.01438 -0.00020 -0.00101 -0.11990

6 32511.6 307.6 0.010328726 0.10459 -0.07408 -0.31480 0.00020

7 32714.2 305.7 0.061327924 0.61716 0.00055 -0.00005 0.78559

8 32959.6 303.4 0.217852565 2.17599 1.47070 0.11420 -0.00014

9 34994.8 285.8 0.041240979 0.38797 0.01030 0.00452 0.62277

10 35003.2 285.7 0.072717192 0.68392 0.75552 0.33619 -0.00933

11 35381.2 282.6 0.092782201 0.86331 -0.00308 -0.00163 0.92914

12 35495.6 281.7 0.287107912 2.66285 1.45368 0.74138 0.00229

13 37390.6 267.4 0.001596132 0.01405 -0.03524 -0.11319 -0.00022

14 37394.3 267.4 0.000141478 0.00125 -0.00026 -0.00075 0.03528

15 39362.4 254.0 0.000266778 0.00223 -0.00006 0.00006 -0.04724

16 39444.0 253.5 0.002582787 0.02156 0.08830 0.11731 0.00003

17 40609.5 246.2 0.001755394 0.01423 0.04377 0.11094 0.00256

18 40658.5 246.0 0.014669464 0.11878 0.00104 0.00262 -0.34463

19 41556.9 240.6 0.000025308 0.00020 -0.00966 -0.01030 -0.00104

20 41565.2 240.6 0.001191584 0.00944 -0.00002 0.00007 0.09715

21 41737.8 239.6 0.217487607 1.71546 -0.00116 0.00261 -1.30975

22 41873.9 238.8 0.415926439 3.27001 -0.61239 1.70146 0.00229

23 43848.1 228.1 0.019882677 0.14928 0.17177 0.34609 -0.00027

24 44112.6 226.7 0.013834932 0.10325 0.00495 -0.00024 -0.32129

25 44180.7 226.3 0.103182106 0.76886 -0.87622 0.03297 -0.00205

26 44621.7 224.1 0.002855398 0.02107 0.00002 -0.00002 0.14514

27 44811.6 223.2 0.000039502 0.00029 -0.00016 0.00012 0.01703

28 45010.3 222.2 0.042348801 0.30975 0.55281 0.06437 -0.00030

29 45595.4 219.3 0.105990454 0.76528 0.00023 -0.00261 0.87480

30 45677.7 218.9 0.065387192 0.47126 -0.03922 0.68536 0.00334

31 46154.2 216.7 0.023540804 0.16791 -0.00004 0.00027 0.40977

32 46570.1 214.7 0.019644616 0.13887 -0.03088 0.37137 0.00010

33 46720.4 214.0 0.092418190 0.65122 -0.35098 0.72666 0.00060

34 46773.0 213.8 0.037585232 0.26454 0.00031 -0.00098 0.51434

35 47656.7 209.8 0.009872245 0.06820 -0.09361 0.24379 -0.00009

36 47690.9 209.7 0.000188618 0.00130 -0.00013 0.00061 -0.03608

37 48315.1 207.0 0.023170050 0.15788 -0.03893 -0.39539 0.00526

38 48318.0 207.0 0.005312293 0.03619 0.00107 0.01090 0.18993

39 48977.3 204.2 0.002636125 0.01772 -0.00052 0.00013 0.13311

40 49170.0 203.4 0.030359777 0.20327 -0.44567 -0.06815 -0.00015

41 49308.8 202.8 0.003299749 0.02203 0.00121 0.00051 0.14842

42 49470.6 202.1 0.055926880 0.37218 -0.35943 -0.49294 -0.00007

43 50133.8 199.5 0.034802401 0.22854 -0.01031 0.00411 -0.47793

44 50144.6 199.4 0.018905305 0.12412 0.33320 -0.11341 -0.01535

45 50295.5 198.8 0.028048433 0.18359 -0.18011 -0.38878 -0.00126

46 50330.3 198.7 0.015128040 0.09895 -0.00097 -0.00197 0.31456

47 50430.5 198.3 0.032990593 0.21536 -0.21119 -0.41323 -0.00076

48 50497.6 198.0 0.005562007 0.03626 -0.00001 -0.00035 0.19042

49 50818.1 196.8 0.036124648 0.23402 -0.38408 -0.29412 -0.00019

50 50947.1 196.3 0.000721031 0.00466 -0.00014 -0.00001 0.06826

**Supplementary Table 3. DFT Optimized xyz coordinates for [Zn(dipyvd)_2_]^+^**

Zn 11.35110296293298 6.68797473060744 15.71600677916606

N 12.20226138769262 5.24390978238725 16.76525208917750

N 9.65639378870957 7.48070361191793 16.83895483181538

N 10.48049439035716 4.90227187546174 14.94184391521889

N 12.93269368764101 7.75644891313227 16.83679061707979

N 12.54076514092870 6.95962284377133 13.77933435294017

N 10.52822719013534 8.29120942204179 14.52259135652409

C 9.55752421202894 9.00462083206895 15.02943482960736

C 11.02898688964609 3.76945348565344 15.39423294813683

N 9.60727684416464 10.48392840767058 13.35485026092524

N 9.05315302060371 10.08408688335030 14.48648281489879

C 9.53431657593909 4.85544641135868 14.00394824588426

H 9.13415584272752 5.81024516448171 13.68662218581757

C 9.01983937768197 8.52094236898263 16.31465714258108

C 12.10837826879814 7.82812368899603 12.88715418695125

N 11.07037535392351 8.67662626404995 13.36611451674836

C 9.24356129526310 6.98048399936690 17.98877220652879

H 9.80039000736649 6.13597430419062 18.37277641147955

C 13.49144975347923 6.10312446418098 13.44747144237395

H 13.80836355322940 5.41298394876062 14.21822395576833

C 14.05493571444159 6.07484909284412 12.19271513633958

H 14.83120265546024 5.36418892785096 11.95760363248225

C 9.07338026512044 3.69475469988186 13.45912490959694

H 8.30093057214170 3.70343558071964 12.70733634965477

C 9.64294342548004 2.51129756127529 13.91931286798651

H 9.32037349472796 1.56039579666136 13.52061948350255

O 11.24363244573708 10.32080793989718 11.79602322377308

C 10.61432277856544 2.52870154198765 14.87956763308450

H 11.04586282285393 1.61253600345037 15.23361538845659

C 7.93362064260608 9.11009390169787 16.92593486207188

H 7.44063699823847 9.95453064736411 16.47105758812424

C 7.50108329100579 8.58492789910040 18.12780355862704

H 6.65172126509007 9.01876227832068 18.63460772086713

C 7.55641075038941 11.60714087416895 12.60755099143439

H 7.29100574274121 10.74225547040969 12.00350126430729

H 7.19268630468626 12.49092695966201 12.08964870662042

H 7.03241847482268 11.54132026119059 13.55759931295341

C 8.16271787447541 7.50387241094648 18.67089229583691

H 7.85330245821865 7.06756923658625 19.60772208364384

C 10.69143121935592 9.87067559268763 12.75209857799854

C 13.58303912697203 6.97059684736154 11.25584673447546

H 13.98033618633834 6.97423649715610 10.25154608708837

C 12.59274637392493 7.87066436479867 11.59027419769281

H 12.22289805459094 8.56394920917659 10.85860295194672

C 9.05720600888566 11.73021708297059 12.78565282343070

N 11.99741418040328 3.93143570048714 16.35254970153088

C 14.85194089899665 7.32191774362162 18.16259685417295

H 15.47653590706388 6.59121682952015 18.65129001608401

O 12.33877167773200 1.74326989795150 16.91440321257760

C 13.18843659102540 9.05351352633823 16.91157695398521

H 12.49907494722298 9.71655932074310 16.40461890671975

C 14.27099121018424 9.55989143040091 17.59132931498040

H 14.44390400495434 10.62433590697895 17.62348021295936

N 14.16980293531717 4.59780700276948 17.83574135068797

C 13.74214239972475 6.89893520157456 17.45171226399773

C 13.37794965681363 5.47111294513027 17.34625685612829

C 15.12060520807757 8.66993266106550 18.22445380882836

H 15.98263493661438 9.02906538853233 18.76736391913315

C 12.65558677471873 2.89649392167915 17.03711628840711

N 13.67051788279128 3.31839924253682 17.82959907416124

C 14.47667935658036 2.35504458827179 18.57686734543682

H 13.91562756305999 1.42441707267366 18.56595833591613

C 14.63711487796209 2.80045835015560 20.01971996152909

H 15.22605774850487 3.71100147582781 20.09896235756999

H 15.14773975798550 2.02369957593356 20.58410999066900

H 13.67223678800983 2.97321115577372 20.49183020532273

C 15.81442781039144 2.12618345004656 17.89447990732048

H 15.68346012076247 1.76148393865109 16.87776944626597

H 16.38835292810504 1.38066598725626 18.44029658587784

H 16.40179932508129 3.04161628541278 17.86222606067037

H 9.51800815586179 11.83614861573097 11.80783379246406

C 9.45456870847382 12.91359767190975 13.64846027374467

H 9.03371910850781 12.83653794122188 14.64877130855262

H 9.07841953539534 13.82891873926034 13.19874237878753

H 10.53531251355784 13.00791235194642 13.72730284389855

**Supplementary Table 4. TD-DFT calculated electronic spectra for Zn(dipyvd)_2_]^+^**

-----------------------------------------------------------------------------

ABSORPTION SPECTRUM VIA TRANSITION ELECTRIC DIPOLE MOMENTS

-----------------------------------------------------------------------------

State Energy Wavelength fosc T2 TX TY TZ

(cm-1) (nm) (au**2) (au) (au) (au)

-----------------------------------------------------------------------------

1 12384.2 807.5 0.000192879 0.00513 0.03957 -0.03376 0.04921

2 18524.5 539.8 0.000000042 0.00000 -0.00047 -0.00066 -0.00031

3 22900.0 436.7 0.066206004 0.95178 0.72061 -0.58977 -0.29098

4 23239.3 430.3 0.000441882 0.00626 -0.00930 0.07355 0.02763

5 24086.8 415.2 0.002556872 0.03495 -0.01216 0.11942 0.14331

6 25068.2 398.9 0.058834653 0.77265 0.05083 -0.87655 0.04151

7 25353.1 394.4 0.014869791 0.19309 0.06140 -0.42975 0.06806

8 25877.8 386.4 0.001960802 0.02494 0.04801 -0.13943 0.05655

9 27434.0 364.5 0.042171542 0.50607 0.39011 -0.57891 0.13692

10 27869.2 358.8 0.000094116 0.00111 0.00199 0.02053 -0.02620

11 28703.2 348.4 0.000025373 0.00029 0.01226 -0.00775 0.00898

12 29533.3 338.6 0.013470876 0.15016 0.25323 -0.11940 0.26791

13 29893.0 334.5 0.004181597 0.04605 0.13864 -0.08561 0.13965

14 29941.9 334.0 0.000251506 0.00277 -0.02923 0.00292 -0.04362

15 30128.3 331.9 0.004582233 0.05007 -0.12672 0.07359 -0.16911

16 30801.9 324.7 0.011324772 0.12104 -0.22412 -0.13011 -0.23212

17 30900.9 323.6 0.000868043 0.00925 0.07506 -0.00042 -0.06011

18 31040.1 322.2 0.013707425 0.14538 -0.26945 -0.08612 -0.25566

19 31408.6 318.4 0.025698754 0.26936 -0.39449 -0.18764 -0.28023

20 32373.8 308.9 0.105726980 1.07515 -0.69880 0.24114 -0.72711

21 32455.2 308.1 0.000207228 0.00210 -0.03290 0.00704 -0.03115

22 32900.6 303.9 0.011212613 0.11220 0.08677 0.25714 -0.19633

23 33142.7 301.7 0.024101129 0.23940 0.38538 0.08485 0.28928

24 33788.8 296.0 0.008619732 0.08398 0.24760 -0.03761 0.14581

25 34494.5 289.9 0.019467409 0.18580 -0.33022 -0.07465 -0.26679

26 34646.9 288.6 0.000837206 0.00796 0.00947 0.08693 0.01758

27 35516.9 281.6 0.000954702 0.00885 0.03559 -0.05538 -0.06720

28 35614.4 280.8 0.213443418 1.97302 1.01078 0.34638 0.91180

29 35848.7 279.0 0.010803598 0.09921 0.17450 -0.22693 0.13139

30 35918.5 278.4 0.000007907 0.00007 0.00297 0.00797 0.00042

31 36558.6 273.5 0.118884244 1.07056 -0.73480 0.38474 0.61854

32 36829.7 271.5 0.000173172 0.00155 0.02291 -0.01677 -0.02724

33 37487.9 266.8 0.000035624 0.00031 0.01266 -0.00698 -0.01019

34 37866.6 264.1 0.001751258 0.01523 0.05402 0.07377 0.08286

35 38312.0 261.0 0.090233032 0.77537 0.66467 -0.41134 -0.40544

36 38726.5 258.2 0.005578189 0.04742 -0.12372 -0.00996 0.17893

37 38870.1 257.3 0.000088609 0.00075 -0.00422 -0.01128 -0.02460

38 38992.0 256.5 0.004375722 0.03694 0.13187 -0.13836 -0.02026

39 39106.2 255.7 0.000533328 0.00449 0.03791 -0.02479 0.04937

40 39643.5 252.2 0.002181888 0.01812 -0.05927 0.10071 0.06682

41 39674.1 252.1 0.000332481 0.00276 -0.03051 0.02583 0.03407

42 39875.2 250.8 0.000742949 0.00613 0.05436 -0.05617 0.00483

43 39913.8 250.5 0.003050432 0.02516 -0.03204 0.10060 -0.11838

44 40008.7 249.9 0.053971865 0.44411 -0.11319 -0.28106 0.59355

45 40019.0 249.9 0.072025974 0.59251 -0.15290 -0.31472 0.68563

46 40254.8 248.4 0.006329958 0.05177 -0.05667 0.00083 0.22035

47 40304.9 248.1 0.002402016 0.01962 -0.04334 -0.03339 0.12894

48 40693.5 245.7 0.064122968 0.51876 0.19239 0.69340 0.03074

49 40866.6 244.7 0.055339688 0.44580 -0.40312 -0.53135 -0.03113

50 41110.8 243.2 0.027406969 0.21947 -0.02668 -0.45827 0.09355

**Supplementary Table 5. DFT Optimized coordinates for [Zn(dipyvd)_2_]^2+^**

Zn -8.04574470331251 -7.89208152716981 -11.75539902993405

N -8.88981243023800 -3.23226245108619 -11.48884498125688

C -7.74213451920366 -5.06189631935313 -10.92073553101758

N -8.48126382159719 -5.84292378493947 -11.66256043282266

C -9.75628281174288 -3.93991606847267 -12.31161696807802

N -6.59949569367432 -7.05717365253510 -10.32989917072769

C -6.66848078843240 -5.73750261748234 -10.16878269307487

C -5.80332287246304 -5.04263835095651 -9.35026162055209

H -5.89182465972075 -3.97252543454072 -9.24850219248841

C -4.83209806908443 -5.75238853989204 -8.67140274450203

H -4.13845594771385 -5.23936651654516 -8.02155225380569

C -4.76310882686777 -7.12059883571511 -8.83208988239611

H -4.02267594974719 -7.71126696169787 -8.31539929101940

C -5.66966588915493 -7.73044439963606 -9.67425544252959

H -5.64284269374945 -8.80158087684543 -9.82278764750220

N -9.73026260334280 -7.48829202508003 -13.08831633099165

C -10.19662665376628 -6.24899700393075 -13.16627166814211

C -11.28674090154593 -5.92194124368043 -13.95860594393003

H -11.66820993275565 -4.92114356478660 -14.02323753126369

C -11.88490872897630 -6.93624238662013 -14.67662549351248

H -12.73637247518936 -6.70562449607089 -15.30001074121182

C -11.40100609069251 -8.22597247508941 -14.59954852482945

H -11.84966201848342 -9.03599491031037 -15.15244994970908

C -10.31705414414302 -8.45044962379160 -13.78754182476906

H -9.89610339183435 -9.44226876320683 -13.68999955034540

C -6.02731866160528 -13.75350621383210 -11.53538426322173

H -6.82033415737754 -14.41452391627270 -11.19342007332382

C -5.54307895155495 -14.23048620187180 -12.88826933847339

H -5.23696020952645 -15.26840649787810 -12.78721895153449

H -4.68505583777803 -13.66819813388483 -13.24492212783814

H -6.32579152306973 -14.19405757532195 -13.64245307448243

C -4.92097725080233 -13.72076314327213 -10.49542141959291

H -5.28840418929958 -13.43241545494438 -9.51365695901811

H -4.12176771525620 -13.04296383021136 -10.79143750170249

H -4.49048278099319 -14.71412079823447 -10.39979778199087

N -6.65090996629093 -12.41019029560891 -11.64232260226281

C -7.67891310286796 -12.08544320610465 -10.76627192587333

C -6.60177651770516 -10.35954119677327 -12.52707719688259

N -8.16922926949610 -10.78575499724313 -10.93096620072373

N -7.60373598405435 -9.94180201179503 -11.79953735982612

N -6.09658569888087 -11.56673315114944 -12.48749104601043

O -8.09692369549716 -12.84593328127380 -9.95369817200716

N -9.32422873461789 -8.90340220808590 -10.27743931641661

C -9.22621081186134 -10.22107465055002 -10.17130580437140

C -10.10738981291878 -10.97117144198632 -9.40896846503598

H -10.03830026336508 -12.04041745840068 -9.33843762255419

C -11.10296650591541 -10.29722744289247 -8.73367590942627

H -11.80658783766427 -10.85861429205157 -8.13658361403855

C -11.19814826246497 -8.92321481351186 -8.82479899060383

H -11.96364137949372 -8.37125673445343 -8.30275438097841

C -10.28515293139963 -8.27161703643775 -9.61667244195661

H -10.32188517785421 -7.19642598891834 -9.72875050086720

N -6.53791994883965 -8.12894011340829 -13.33413786968639

C -6.02924612234556 -9.35484497974522 -13.44198119094312

C -5.03915199552607 -9.67213053634532 -14.34732567957789

H -4.65503497141146 -10.67852107634360 -14.40369239098532

C -4.56358975827114 -8.67239047002697 -15.17340714379060

H -3.78880682179436 -8.88600403156657 -15.89503444803478

C -5.09210435671500 -7.40291758004732 -15.06623355783332

H -4.74925504659519 -6.59619527608426 -15.69527728680474

C -6.07992118859905 -7.17750402473125 -14.12965627339561

H -6.51533566948016 -6.19349955036334 -14.01771530595559

C -9.03264143862846 -1.76163754170851 -11.35309219779186

H -9.92805660631968 -1.50228162203194 -11.90953066845340

C -9.23153387032806 -1.39033884868234 -9.89659166620616

H -9.41998378095873 -0.32192340867730 -9.83292415121232

H -8.35221853885346 -1.60534828192768 -9.29462964502638

H -10.08981560798723 -1.89780580759354 -9.46193549133920

C -7.83730293313603 -1.07255584788653 -11.98394076064596

H -7.72541017462515 -1.33530665295373 -13.03360712322337

H -6.91334710569993 -1.30789059295726 -11.46026536109198

H -7.98126894152729 0.00334825982271 -11.93187251778455

N -7.89757741230120 -3.76758962135793 -10.80944509723429

N -9.48221538622233 -5.31238840351183 -12.37146448133855

O -10.64621847679093 -3.42401918947604 -12.90743921021773

**Supplementary Table 6. TD-DFT calculated electronic spectra for Zn(dipyvd)_2_]^2+^**

-----------------------------------------------------------------------------

ABSORPTION SPECTRUM VIA TRANSITION ELECTRIC DIPOLE MOMENTS

-----------------------------------------------------------------------------

State Energy Wavelength fosc T2 TX TY TZ

(cm-1) (nm) (au**2) (au) (au) (au)

-----------------------------------------------------------------------------

1 22396.8 446.5 0.085082586 1.25063 -0.28002 -0.79239 -0.73779

2 22542.4 443.6 0.068305797 0.99754 -0.92437 0.15310 0.34589

3 24025.5 416.2 0.001521075 0.02084 -0.09215 -0.01956 0.10940

4 24226.7 412.8 0.001689314 0.02296 -0.12472 -0.05416 -0.06684

5 26933.0 371.3 0.065660889 0.80260 -0.35993 0.81878 0.05152

6 27153.6 368.3 0.031832549 0.38594 0.04530 0.60976 0.10991

7 30810.4 324.6 0.000788613 0.00843 -0.06663 -0.03618 -0.05175

8 30836.0 324.3 0.000413246 0.00441 -0.03469 0.03301 0.04603

9 32762.9 305.2 0.043025207 0.43233 0.18406 -0.62993 0.04047

10 32887.8 304.1 0.011333485 0.11345 -0.11528 0.31380 -0.04111

11 34130.5 293.0 0.003530851 0.03406 -0.16163 -0.05101 0.07302

12 34152.5 292.8 0.004779222 0.04607 -0.09595 0.02842 -0.18988

13 34356.9 291.1 0.000002739 0.00003 0.00275 -0.00200 0.00383

14 34689.4 288.3 0.000155052 0.00147 -0.03347 -0.00719 0.01731

15 35370.4 282.7 0.001170678 0.01090 0.02922 0.02405 -0.09728

16 35399.0 282.5 0.010629187 0.09885 -0.12678 0.28087 -0.06236

17 35444.3 282.1 0.005651861 0.05250 0.02475 -0.19905 0.11074

18 35581.8 281.0 0.010080881 0.09327 -0.19622 0.12115 0.20022

19 36516.6 273.8 0.151876431 1.36923 -0.67776 -0.42255 -0.85517

20 36593.0 273.3 0.146912560 1.32171 -0.96039 0.00872 0.63188

21 38000.2 263.2 0.048991760 0.42444 0.01148 -0.24314 -0.60431

22 38077.7 262.6 0.049636510 0.42915 -0.62686 -0.19025 -0.00062

23 38263.5 261.3 0.004765547 0.04100 -0.13465 -0.14927 -0.02430

24 38300.3 261.1 0.006836478 0.05876 -0.13072 0.20115 0.03482

25 38401.4 260.4 0.013765241 0.11801 -0.09336 -0.29170 -0.15558

26 38438.9 260.2 0.008836315 0.07568 -0.00596 -0.26644 -0.06820

27 38457.2 260.0 0.025442013 0.21780 0.23249 -0.25839 -0.31142

28 38676.5 258.6 0.000396975 0.00338 -0.05759 0.00651 -0.00448

29 38911.5 257.0 0.016339498 0.13824 -0.21640 -0.14093 -0.26749

30 39203.3 255.1 0.002525403 0.02121 -0.03441 0.14138 0.00595

31 39287.9 254.5 0.005811249 0.04870 -0.12872 0.00772 0.17908

32 39505.4 253.1 0.069448433 0.57874 0.11025 0.66834 -0.34626

33 39831.5 251.1 0.180689854 1.49342 0.75582 -0.88847 0.36440

34 40246.0 248.5 0.097370770 0.79649 0.30774 0.69256 -0.47132

35 40314.0 248.1 0.004817729 0.03934 -0.11347 -0.07107 0.14634

36 40439.0 247.3 0.005641971 0.04593 0.01771 0.03511 -0.21068

37 41359.5 241.8 0.003880649 0.03089 -0.03760 0.14047 0.09871

38 41492.9 241.0 0.010716471 0.08503 0.20915 -0.10071 -0.17647

39 41674.3 240.0 0.053291773 0.42099 0.48139 0.27656 0.33580

40 41830.4 239.1 0.021886578 0.17225 -0.19201 0.25611 0.26417

41 42118.3 237.4 0.003561649 0.02784 0.09710 -0.13156 -0.03320

42 42241.2 236.7 0.004053173 0.03159 -0.01220 0.17274 0.04000

43 42606.2 234.7 0.034553189 0.26699 -0.49180 -0.05413 0.14896

44 42628.6 234.6 0.035707621 0.27576 -0.46246 -0.21515 0.12493

45 42823.1 233.5 0.075215335 0.57823 -0.02969 0.61477 -0.44655

46 42836.9 233.4 0.127028959 0.97625 -0.26108 0.78983 -0.53315

47 42896.0 233.1 0.054073521 0.41500 0.43733 -0.38453 0.27545

48 43007.9 232.5 0.078023860 0.59725 -0.10505 0.63542 -0.42715

49 43638.5 229.2 0.074838192 0.56458 -0.10635 0.70198 -0.24596

50 43796.5 228.3 0.037929585 0.28511 -0.24588 0.46325 -0.10026

**Supplementary Table 7. DFT Optimized coordinates for Ni(dipyvd)_2_**

Ni 15.78851966356619 -0.06614420381083 3.81771860523075

N 11.30774876206464 1.05582780818249 3.82398094758647

C 13.02193698748383 -0.11043090831966 3.00518121014526

N 13.85890927977915 0.34225661458953 3.91823018069579

C 12.06889616435681 1.74388529039064 4.70629320263374

N 14.92067847314019 -1.34686376679632 2.28358981152219

C 13.62458434371143 -1.11550190823992 2.10284260081241

C 12.89176303562196 -1.77293190102964 1.13168047128677

H 11.84096922855516 -1.55590900611967 1.02348855773639

C 13.53922990318701 -2.68455618150733 0.32753172531247

H 12.99627879168238 -3.20907690086902 -0.44558114102599

C 14.88734146561616 -2.92125702734836 0.52025735526092

H 15.42810534798209 -3.62787483628493 -0.09015900326753

C 15.53834092418679 -2.22771832957587 1.51743669971432

H 16.59298719154602 -2.37388382080734 1.71153856708468

N 15.64028351163756 1.52122445345358 5.23849295385062

C 14.43055429820994 2.07579648511688 5.35482746417675

C 14.24782410249393 3.22000070701331 6.14769103666418

H 13.28340085157389 3.68106834889308 6.23872408509788

C 15.33007645262230 3.73526535849993 6.80644952796407

H 15.19768299723950 4.61639390071809 7.41815645676997

C 16.58105440160784 3.14277095730189 6.69207723230297

H 17.44803254104706 3.52931549931390 7.20310368492063

C 16.67564131042402 2.04206487660443 5.88714932194574

H 17.62417194974028 1.54050809331482 5.74292270857800

C 21.63859656311076 -1.62955631064672 3.88390717727145

H 22.15270770782221 -1.19895051272974 3.02881213879579

C 22.35842298043860 -1.19155849657865 5.14955667887826

H 23.38320692327820 -1.55782285985196 5.13860430521762

H 21.86963529700533 -1.58193688604501 6.03924803174817

H 22.39412857416005 -0.10688465989872 5.22655275006120

C 21.63249222464995 -3.14207809431476 3.72955141396391

H 21.12867320471099 -3.44211580887170 2.81293777893514

H 21.13536336028876 -3.62716292989108 4.56670081974718

H 22.65450976508511 -3.51324875671206 3.68422563832321

N 20.28238113797921 -1.10367153232430 3.84802698445630

C 19.91719144931603 -0.18549388171351 2.92399725641364

C 18.22934529806983 -1.31851090703834 4.68749164233067

N 18.57471085221525 0.20140130547448 3.00920728780830

N 17.68830960052398 -0.60963338217530 3.71266871631548

N 19.47514496163577 -1.56105976123634 4.87280544878635

O 20.66918379271905 0.26974381661663 2.09729973105380

N 16.64881608375943 1.26447863205785 2.39054615672139

C 17.97965994898766 1.21124344082530 2.29416281722838

C 18.67611409096007 2.16110368898948 1.53065848859065

H 19.74634689251381 2.13378636122460 1.45648224681759

C 17.95810899362144 3.12791886073350 0.88226925480018

H 18.48661224453631 3.86684972320097 0.29682778431867

C 16.57237189148361 3.16617186594528 0.97450219252938

H 15.98410252817871 3.91508425928910 0.46914961931561

C 15.97406965307641 2.21108610993465 1.74797966515414

H 14.89873809564698 2.19033504924103 1.87214228303020

N 15.97420545773782 -1.55333191144115 5.40274164626178

C 17.23813789218050 -1.90294594353279 5.61651616196318

C 17.59825857997544 -2.76120645152793 6.63919801552292

H 18.63659516214176 -3.02072317976577 6.77169124754562

C 16.60856319475227 -3.25314953653334 7.46118530625096

H 16.85797189834567 -3.91969064866881 8.27428345155508

C 15.29499529667407 -2.88732227059994 7.23345116720843

H 14.49232374673343 -3.25263366639723 7.85516991849907

C 15.02514710859828 -2.03362860302080 6.18577976611400

H 14.01501391573694 -1.71388949045605 5.96460073557752

C 9.86713823734376 1.25289539688367 3.77218252144399

H 9.62834468990969 1.95393018859653 4.56695770334394

C 9.45158349123662 1.86775532123889 2.44498251006070

H 8.38000420223308 2.05827770356897 2.44595112696623

H 9.67666234478077 1.20660991963404 1.61165687361379

H 9.95701376166194 2.81627822232888 2.27308732994566

C 9.13768349041443 -0.05271295000559 4.04409347695952

H 9.41319044167755 -0.46256215280751 5.01388529830995

H 9.35808984717921 -0.79836007883033 3.28331408630117

H 8.06241171375833 0.11539974753648 4.04763925209668

N 11.78858048426517 0.20321808354498 2.84713416008627

N 13.43707389028971 1.45725839074136 4.63491180700545

O 11.62191505752125 2.54218597332509 5.49346786575530

**Supplementary Table 8. TD-DFT calculated electronic spectra for Ni(dipyvd)_2_**

-----------------------------------------------------------------------------

ABSORPTION SPECTRUM VIA TRANSITION ELECTRIC DIPOLE MOMENTS

-----------------------------------------------------------------------------

State Energy Wavelength fosc T2 TX TY TZ

(cm-1) (nm) (au**2) (au) (au) (au)

-----------------------------------------------------------------------------

1 12682.0 788.5 0.000019893 0.00052 0.01985 -0.00231 0.01082

2 13090.7 763.9 0.001040420 0.02617 0.06918 -0.04779 0.13818

3 13258.6 754.2 0.000704327 0.01749 -0.05094 -0.11752 -0.03291

4 17915.5 558.2 0.000033708 0.00062 0.01826 -0.00042 0.01691

5 18093.8 552.7 0.000058433 0.00106 -0.02843 -0.00121 -0.01591

6 18506.8 540.3 0.000053205 0.00095 -0.00370 0.03037 0.00322

7 18745.2 533.5 0.000073215 0.00129 -0.03445 -0.00092 0.00992

8 24321.0 411.2 0.067719601 0.91666 0.83517 -0.38825 -0.26156

9 24530.1 407.7 0.003625409 0.04866 -0.21295 -0.02462 0.05198

10 24687.6 405.1 0.024604641 0.32811 -0.51375 -0.22181 0.12235

11 25283.5 395.5 0.000059500 0.00077 -0.02549 -0.00717 -0.00858

12 25657.9 389.7 0.000028621 0.00037 -0.01452 0.01012 -0.00734

13 28063.7 356.3 0.000033963 0.00040 0.00364 0.01555 0.01197

14 28303.8 353.3 0.000053786 0.00063 -0.00474 0.01999 -0.01427

15 29154.3 343.0 0.012741538 0.14388 -0.29974 -0.10705 -0.20633

16 29580.3 338.1 0.005807273 0.06463 0.20159 0.10969 0.10937

17 29703.9 336.7 0.008107810 0.08986 0.16384 -0.18281 0.17204

18 29947.9 333.9 0.017290532 0.19007 0.32538 0.01298 0.28988

19 30000.2 333.3 0.007947435 0.08721 -0.07431 0.27562 -0.07565

20 30490.8 328.0 0.017171202 0.18540 -0.18071 0.27552 -0.27719

21 30754.0 325.2 0.031718130 0.33953 0.31095 0.29653 0.39359

22 31139.8 321.1 0.019192016 0.20290 -0.11551 0.28314 -0.33074

23 31434.8 318.1 0.115026893 1.20466 0.97938 0.27048 0.41511

24 31708.6 315.4 0.108154790 1.12291 0.66971 -0.70412 0.42262

25 32174.6 310.8 0.000019553 0.00020 0.01007 -0.00783 0.00611

26 32264.8 309.9 0.000028159 0.00029 -0.00888 -0.00989 -0.01052

27 32495.6 307.7 0.001399377 0.01418 0.00321 -0.11777 0.01720

28 32962.3 303.4 0.001957063 0.01955 0.11767 -0.01570 0.07384

29 33111.4 302.0 0.007363118 0.07321 -0.19656 -0.06799 -0.17306

30 33390.3 299.5 0.011923637 0.11756 -0.22847 -0.20372 -0.15448

31 33535.1 298.2 0.003624197 0.03558 -0.13076 -0.12958 -0.04110

32 33690.6 296.8 0.057017321 0.55715 -0.47867 -0.14113 -0.55507

33 33774.7 296.1 0.016086613 0.15680 0.00592 -0.39568 -0.01420

34 34065.4 293.6 0.081295622 0.78565 -0.44516 0.30764 -0.70203

35 34194.0 292.4 0.104554435 1.00663 -0.35031 -0.92396 -0.17380

36 34540.8 289.5 0.059131860 0.56359 -0.07435 0.64805 -0.37161

37 34799.1 287.4 0.009725733 0.09201 0.20537 0.16364 0.15184

38 35105.7 284.9 0.018091265 0.16966 0.24367 0.09864 0.31710

39 35223.2 283.9 0.002926062 0.02735 0.07625 -0.10728 0.10013

40 35555.2 281.3 0.018108093 0.16767 0.16589 -0.22208 0.30138

41 35718.8 280.0 0.036981384 0.34085 0.42800 -0.02567 0.39625

42 35816.9 279.2 0.006473912 0.05951 -0.11801 0.15601 -0.14573

43 35964.8 278.1 0.017797004 0.16291 -0.28966 0.01186 -0.28083

44 36245.0 275.9 0.022286671 0.20243 -0.21640 0.28548 -0.27221

45 36852.1 271.4 0.000081864 0.00073 -0.00348 -0.02552 -0.00826

46 36954.4 270.6 0.001920203 0.01711 -0.06017 0.06895 -0.09344

47 38693.2 258.4 0.000169681 0.00144 -0.01861 0.00921 -0.03182

48 38914.7 257.0 0.000614397 0.00520 0.03990 0.04452 0.04030

49 38996.3 256.4 0.000755054 0.00637 -0.04802 0.00426 -0.06364

50 39209.3 255.0 0.000406122 0.00341 0.03898 0.01200 0.04179

**Supplementary Table 9 DFT Optimized coordinates for [Ni(dipyvd)_2_]^+^**

Ni 11.443401 6.834948 15.777936

N 12.125519 5.346354 16.845749

N 9.853685 7.600642 17.006545

N 10.367873 5.202794 15.041713

N 13.039288 7.784929 16.856658

N 12.645149 6.949895 13.958470

N 10.734152 8.402580 14.703300

C 9.698529 9.066365 15.154794

C 10.813161 4.011528 15.463300

N 9.622895 10.389693 13.357150

N 9.109844 10.051296 14.527317

C 9.384534 5.259434 14.144590

H 9.066115 6.250510 13.847681

C 9.196564 8.623655 16.468524

C 12.362720 7.933257 13.118664

N 11.251752 8.730475 13.514538

C 9.473230 7.133935 18.180061

H 10.041926 6.300648 18.570583

C 13.661539 6.143198 13.700801

H 13.846116 5.356852 14.420204

C 14.454135 6.282934 12.585305

H 15.273275 5.604025 12.409682

C 8.786603 4.157220 13.609707

H 7.991099 4.254196 12.888832

C 9.248704 2.917137 14.037512

H 8.813990 2.008720 13.646443

O 11.070490 10.042682 11.655256

C 10.252565 2.826174 14.959639

H 10.599849 1.868063 15.295408

C 8.123661 9.226485 17.089657

H 7.614687 10.055059 16.623051

C 7.726097 8.735850 18.318105

H 6.888491 9.181002 18.834494

C 7.685279 10.833161 11.928707

H 7.969059 10.053937 11.224956

H 7.160353 11.602711 11.368399

H 6.987381 10.415889 12.652591

C 8.407655 7.674468 18.874450

H 8.124411 7.264584 19.831353

C 10.683594 9.746406 12.743614

C 14.163719 7.314065 11.718267

H 14.759936 7.470036 10.831348

C 13.106344 8.162335 11.972060

H 12.884937 8.962935 11.293426

C 8.894861 11.441147 12.616009

N 11.825096 4.064725 16.384799

C 14.959979 7.227256 18.137837

H 15.551479 6.455307 18.604120

O 12.067548 1.838247 16.836379

C 13.369521 9.064718 16.941274

H 12.709410 9.772874 16.457757

C 14.492643 9.500061 17.605156

H 14.723602 10.553055 17.647776

N 14.124099 4.539672 17.740284

C 13.813004 6.875994 17.448729

C 13.357118 5.477729 17.338778

C 15.305330 8.557323 18.209070

H 16.196755 8.861401 18.738020

C 12.456000 2.966060 16.986703

N 13.538641 3.296012 17.735332

C 14.345604 2.246236 18.354828

H 13.703578 1.371342 18.413296

C 14.744445 2.640868 19.764681

H 15.424501 3.489059 19.773681

H 15.248241 1.804604 20.243794

H 13.874836 2.893435 20.367816

C 15.548064 1.906537 17.489914

H 15.240558 1.559562 16.505537

H 16.127659 1.110223 17.951747

H 16.203328 2.766985 17.366692

H 9.587346 11.802750 11.861128

C 8.533256 12.591359 13.533086

H 7.786884 12.316029 14.272941

H 8.118155 13.393543 12.928111

H 9.404538 12.988500 14.049079

**Supplementary Table 10. TD-DFT calculated electronic spectra for Ni(dipyvd)_2_]^+^**

-----------------------------------------------------------------------------

ABSORPTION SPECTRUM VIA TRANSITION ELECTRIC DIPOLE MOMENTS

-----------------------------------------------------------------------------

State Energy Wavelength fosc T2 TX TY TZ

(cm-1) (nm) (au**2) (au) (au) (au)

-----------------------------------------------------------------------------

1 11274.2 887.0 0.000364219 0.01064 -0.08085 0.05813 0.02682

2 11576.3 863.8 0.000702545 0.01998 -0.10290 0.06731 0.06971

3 13145.5 760.7 0.000021377 0.00054 0.02041 -0.01069 -0.00212

4 17422.5 574.0 0.000348439 0.00658 -0.06104 -0.03995 -0.03551

5 17885.7 559.1 0.000380611 0.00701 0.02365 -0.07150 0.03652

6 18834.1 531.0 0.000716558 0.01253 -0.01675 0.09713 -0.05302

7 20244.8 494.0 0.000106573 0.00173 -0.03112 0.01901 -0.02008

8 23024.3 434.3 0.054934353 0.78548 -0.67636 0.55193 0.15294

9 23130.0 432.3 0.012489769 0.17777 -0.33682 0.24790 0.05353

10 24051.4 415.8 0.002409093 0.03298 -0.08291 0.16154 0.00266

11 24506.8 408.0 0.063193927 0.84891 -0.03837 0.91669 -0.08437

12 24985.7 400.2 0.002965969 0.03908 0.05951 -0.18383 0.04178

13 25664.6 389.6 0.000455440 0.00584 0.04264 -0.05352 0.03406

14 26793.8 373.2 0.001395133 0.01714 -0.04964 -0.08914 -0.08205

15 27407.9 364.9 0.020855031 0.25050 -0.29129 0.36908 -0.17156

16 27538.0 363.1 0.019313506 0.23089 -0.30885 0.34557 -0.12682

17 27614.9 362.1 0.005459354 0.06508 0.13319 -0.19250 0.10143

18 28474.2 351.2 0.001033253 0.01195 -0.07243 0.05733 -0.05843

19 28717.1 348.2 0.004181060 0.04793 0.15646 -0.08203 0.12931

20 29297.1 341.3 0.007939698 0.08922 -0.17587 0.16281 -0.17827

21 29760.5 336.0 0.001563343 0.01729 0.10620 -0.04189 0.06527

22 29973.1 333.6 0.003819006 0.04195 0.09926 0.13395 -0.11896

23 30528.5 327.6 0.016814369 0.18132 0.02499 0.23311 -0.35547

24 30583.0 327.0 0.016576244 0.17844 0.28717 0.10074 0.29295

25 30831.9 324.3 0.000873585 0.00933 -0.04066 0.04286 -0.07641

26 31061.1 321.9 0.023815350 0.25242 -0.37323 -0.05465 -0.33186

27 31185.4 320.7 0.010295888 0.10869 -0.27429 -0.02999 -0.18043

28 31415.9 318.3 0.045690781 0.47880 0.28709 -0.32885 0.53688

29 32189.9 310.7 0.016032062 0.16396 0.32091 -0.08506 0.23183

30 32227.5 310.3 0.046574896 0.47577 -0.54538 0.16559 -0.38847

31 32680.3 306.0 0.006457828 0.06505 -0.01660 -0.03600 0.25196

32 32702.9 305.8 0.011435399 0.11512 -0.33037 -0.00696 -0.07696

33 33526.6 298.3 0.007648037 0.07510 -0.21842 0.16245 0.03165

34 33675.5 297.0 0.002137224 0.02089 0.09357 -0.10707 0.02595

35 33947.6 294.6 0.008171404 0.07924 -0.21977 -0.04620 -0.16974

36 34508.4 289.8 0.001665538 0.01589 -0.06043 0.10973 -0.01406

37 34826.6 287.1 0.000529283 0.00500 -0.06997 -0.00889 -0.00529

38 35204.3 284.1 0.042939355 0.40155 0.48790 0.23255 0.33079

39 35465.6 282.0 0.156936317 1.45677 -0.89298 -0.21195 -0.78385

40 35752.9 279.7 0.001166311 0.01074 -0.08189 -0.01978 -0.06035

41 36105.8 277.0 0.009027770 0.08232 0.14176 -0.23116 0.09372

42 36290.1 275.6 0.012561984 0.11396 0.24268 0.05910 0.22710

43 36646.3 272.9 0.006276650 0.05639 0.23290 -0.04287 -0.01745

44 37226.3 268.6 0.104200158 0.92150 0.80869 -0.36300 -0.36845

45 37277.5 268.3 0.032920088 0.29073 -0.41162 0.25221 0.24018

46 38356.3 260.7 0.077833444 0.66804 0.64963 -0.42128 -0.26183

47 38684.9 258.5 0.019420750 0.16527 0.30100 -0.14512 -0.23154

48 38815.9 257.6 0.006122227 0.05192 -0.14448 0.06110 0.16527

49 39216.6 255.0 0.001647643 0.01383 0.02527 -0.08292 -0.07948

50 39502.3 253.1 0.001973500 0.01645 0.02806 -0.12237 -0.02616

**Supplementary Table 11 DFT Optimized coordinates for [Ni(dipyvd)_2_]^2+^**

Ni 15.81955213754098 0.07809905750805 3.90831403552169

N 11.33132403001086 1.20121914024608 3.60081623232398

C 13.06723977790606 -0.10245027967745 3.07938111118211

N 13.88031669784473 0.54628955745486 3.87503879712569

C 12.07067036894461 1.96033202785853 4.49795196685035

N 15.00907075252624 -1.33249925982334 2.49619530925664

C 13.69334595497430 -1.19853681989572 2.31958324508929

C 12.96631982762407 -2.02139314335902 1.48643480640878

H 11.90348337774388 -1.87693488211865 1.37173261581925

C 13.63433720388599 -3.02546113133689 0.81287464474515

H 13.09526525694853 -3.68994488744982 0.15384948573347

C 14.99443411091563 -3.16493818911125 0.99230006943349

H 15.55259423656700 -3.93460156311572 0.48231572714970

C 15.64089328907277 -2.29316982253240 1.84463735446130

H 16.70712917634723 -2.37685035569867 2.00525331603183

N 15.59706495500189 1.66119265347709 5.32193064529422

C 14.37146941476925 2.15721946883448 5.46250791072377

C 14.08029565272082 3.15855078429216 6.37523549052673

H 13.08963813863746 3.55432358262548 6.49241053538219

C 15.11424887723266 3.64469044788678 7.14714992031863

H 14.91153319754291 4.42471733117766 7.86624429962956

C 16.38867183549962 3.13710580652995 7.00212937229001

H 17.21527957661680 3.49833266400133 7.59302786974011

C 16.57736927994838 2.14221184314232 6.07465271031978

H 17.55680805356282 1.70842588696040 5.92763239109468

C 21.72497116826721 -1.52187952984537 3.87836593232960

H 22.30627803368173 -0.72362536727707 3.42462831823575

C 22.21869008277911 -1.74040970534946 5.29330179639002

H 23.28242760356357 -1.95844333472851 5.24986723351064

H 21.73107539294308 -2.57944567610213 5.78120879045578

H 22.09437201637760 -0.85445791231353 5.91208001757144

C 21.82982748122311 -2.76674411720439 3.01524391488993

H 21.51615228304297 -2.58122544975001 1.99050196153060

H 21.23979608869958 -3.58473826957934 3.42505804810703

H 22.86604596707768 -3.09260090125733 2.98207944369742

N 20.32346193972057 -1.03485912578654 3.88565213341924

C 19.94519901884879 -0.07151479707154 2.96010839718480

C 18.24720135624760 -1.26894300391089 4.67488980438597

N 18.60198853953371 0.31013083682720 3.06201080669670

N 17.77411040923880 -0.32589325195964 3.89870327667387

N 19.49670072582988 -1.65109839184873 4.70583601734482

O 20.69595877095714 0.38660314088185 2.16051870488060

N 16.65825138840446 1.36673409453797 2.41850385324790

C 17.97898108656540 1.31493042020928 2.28023121976183

C 18.67258025991208 2.19191309002794 1.46206925833913

H 19.74114837395513 2.15889570993943 1.36478001727441

C 17.94324532186939 3.12793201176704 0.75998870248417

H 18.46128000619306 3.82450741937055 0.11732497737202

C 16.56939518224446 3.17065933463370 0.88004427736559

H 15.97308804526531 3.88688297813560 0.33740554945189

C 15.97462657867988 2.26955710192355 1.72831530989393

H 14.90187283595836 2.27048159309629 1.86246627950714

N 16.00129041898457 -1.44505448014745 5.41740435525718

C 17.24673442635373 -1.90612744311997 5.54916671993837

C 17.57982987193484 -2.90713821490553 6.43598751633547

H 18.60022171898397 -3.24901778560967 6.51138849549286

C 16.57700943889352 -3.45237203185337 7.21385115629240

H 16.80214103100853 -4.23974919721943 7.91799299532879

C 15.28926922718552 -2.97799032212651 7.07937198628095

H 14.47890669396940 -3.37731848433489 7.66913351803570

C 15.04810777830972 -1.97080840890687 6.16694519311204

H 14.04915398671183 -1.57666120307408 6.03795406734097

C 9.88528346494209 1.49185789770110 3.43363106463796

H 9.75406372130286 2.51580988897829 3.77261035092232

C 9.48946773875566 1.40571437950064 1.97419204968501

H 8.46687582403147 1.76101600155208 1.87857011152863

H 9.52159621118742 0.38865715923238 1.59449125458420

H 10.11179888492609 2.03615606764339 1.34311550508789

C 9.07657142577768 0.56469942632655 4.32254581633002

H 9.34076660693185 0.67343325952575 5.37199015013590

H 9.20410459864826 -0.47628094561568 4.03040872246440

H 8.02161212835122 0.80766811011339 4.22733999080603

N 11.79831234695605 0.17122319717475 2.92580797883125

N 13.40702177525942 1.55823232256345 4.61238555575041

O 11.60763954312782 2.87017199135948 5.10675154136629

**Supplementary Table 12. TD-DFT calculated electronic spectra for Zn(dipyvd)_2_]^2+^**

-----------------------------------------------------------------------------

ABSORPTION SPECTRUM VIA TRANSITION ELECTRIC DIPOLE MOMENTS

-----------------------------------------------------------------------------

State Energy Wavelength fosc T2 TX TY TZ

(cm-1) (nm) (au**2) (au) (au) (au)

-----------------------------------------------------------------------------

1 13305.0 751.6 0.000000847 0.00002 -0.00328 -0.00124 -0.00294

2 15749.4 634.9 0.000420403 0.00879 0.00917 0.02567 -0.08969

3 15791.7 633.2 0.000462730 0.00965 -0.00772 -0.09458 -0.02531

4 21096.2 474.0 0.000055631 0.00087 0.00846 0.01741 -0.02222

5 21210.3 471.5 0.000492767 0.00765 0.08400 -0.00371 0.02407

6 22559.0 443.3 0.096168211 1.40342 0.84865 -0.26139 0.78415

7 22655.2 441.4 0.048562084 0.70567 -0.23138 -0.80367 -0.07911

8 23057.7 433.7 0.001446427 0.02065 0.09711 -0.03530 0.09987

9 26262.6 380.8 0.001946900 0.02441 0.11691 0.06310 -0.08219

10 26278.5 380.5 0.001474415 0.01847 -0.07517 -0.05461 -0.09919

11 26960.6 370.9 0.097547046 1.19113 -1.03586 0.32518 -0.11126

12 27094.0 369.1 0.008441142 0.10257 0.29718 0.07671 0.09147

13 30784.8 324.8 0.001472470 0.01575 -0.07377 0.00556 0.10136

14 30816.0 324.5 0.000638840 0.00682 -0.00085 0.07653 0.03111

15 32585.6 306.9 0.071469988 0.72206 0.80986 -0.21660 -0.13881

16 32787.2 305.0 0.000923440 0.00927 -0.06585 -0.06353 0.03001

17 33921.3 294.8 0.002602566 0.02526 -0.00244 -0.07362 -0.14083

18 33990.5 294.2 0.000593002 0.00574 -0.02165 -0.04368 0.05802

19 34034.4 293.8 0.007468981 0.07225 -0.03864 -0.21207 0.16056

20 34070.5 293.5 0.006916758 0.06683 -0.04948 -0.14861 -0.20567

21 35536.8 281.4 0.040411506 0.37437 -0.13441 0.10064 0.58837

22 35617.3 280.8 0.001769560 0.01636 0.07996 0.05870 -0.08073

23 35698.4 280.1 0.063392104 0.58460 0.33818 -0.15735 0.66744

24 35745.3 279.8 0.094844548 0.87351 -0.22270 -0.90739 -0.02378

25 36758.0 272.0 0.000547538 0.00490 0.02483 0.04673 -0.04586

26 36931.6 270.8 0.000655110 0.00584 0.03103 0.06982 -0.00163

27 37154.2 269.1 0.000858445 0.00761 -0.00468 0.08547 0.01671

28 37226.3 268.6 0.012619368 0.11160 -0.06139 -0.26354 0.19590

29 37289.4 268.2 0.011116739 0.09814 -0.08511 -0.18537 -0.23778

30 37422.5 267.2 0.003282208 0.02887 -0.04850 0.04716 -0.15588

31 38062.7 262.7 0.042577178 0.36826 0.12279 0.59422 -0.00899

32 38122.1 262.3 0.073110140 0.63136 0.48353 -0.08596 0.62464

33 38488.8 259.8 0.011040376 0.09443 0.24530 -0.15285 0.10438

34 38514.7 259.6 0.005544680 0.04739 0.19110 0.07448 0.07299

35 38821.3 257.6 0.003479653 0.02951 -0.14174 0.03879 -0.08896

36 38956.7 256.7 0.000287131 0.00243 -0.02720 -0.04081 -0.00456

37 39381.2 253.9 0.080099599 0.66960 -0.29269 0.48283 0.59229

38 39491.7 253.2 0.103416771 0.86211 0.77442 0.21184 -0.46637

39 39675.9 252.0 0.108489503 0.90020 -0.53274 0.48877 -0.61440

40 39883.5 250.7 0.078599885 0.64879 0.60870 0.51488 0.11474

41 40042.2 249.7 0.055956597 0.46005 0.36304 -0.53861 -0.19532

42 40163.8 249.0 0.049276818 0.40391 0.58647 -0.24486 0.00129

43 40431.6 247.3 0.037943802 0.30896 0.48511 -0.14177 -0.23135

44 40506.9 246.9 0.007561547 0.06145 0.16629 0.17474 -0.05716

45 41375.8 241.7 0.003464258 0.02756 -0.01995 -0.05068 0.15684

46 41590.5 240.4 0.002648195 0.02096 0.05260 -0.10703 0.08209

47 41784.9 239.3 0.014502821 0.11426 0.20814 -0.00990 0.26616

48 41857.7 238.9 0.014006858 0.11016 0.08294 0.31649 0.05583

49 41962.4 238.3 0.004140066 0.03248 -0.05374 -0.08653 0.14868

50 41996.1 238.1 0.000014194 0.00011 0.00973 -0.00142 -0.00382

**Supplementary Data: XAS/XMCD Simulation details**

Simulations used the ‘CRISPY’ X-ray spectroscopy front end (Retegan, 2019) to the quantum chemistry package ‘Quanty’.(Haverkort et al., 2012)

Atomic parameters for the initial atomic state [F2(d,d), F4(d,d) , ζ(3d)] and final state [F2(d,d) F4(d,d) F2(p,d) G1(p,d) G3(p,d) ζ(2p), ζ(3d)] were taken from tables reported by van der Laan.(van der Laan and Thole, 1991) Values of Fn and Gn were reduced by a factor of 80% to account for correlation effects (these parameters were provided by default in the simulation program). Previous authors have used values for the Hubbard parameter U(3d,3d) varying from 5.0 to 6.5 eV. We fixed the value of U(3d,3d) at 5 eV; we found that the simulation is only marginally dependent on this parameter.

Variation of the octahedral splitting of the d orbitals (10Dq) gave an initial best fit at 1.9 eV. In particular the position of small satellite feature on the high energy side of the L3 edge is quite sensitive to the value of 10Dq. However, just modifying the value of 10Dq left some small discrepancies in the spacing of the peaks in the L3 edge and a better fit was obtained by including metal-ligand hybridization. We chose a value of 5 eV for Δ (the difference in electronegativity between 3d and ligand orbitals) based on other literature reports. We then varied the value of both 10Dq and the ligand-metal transfer integrals V(eg) and V(t2g), holding the ratio of V(eg)/V(t2g) equal to 2. In order to match the linewidth of the experimental spectra a Lorentzian broadening of 0.48 (0.9) eV was applied to the L3 (L2) edge. An additional Gaussian broadening of 0.4 eV was applied to the entire spectrum. Additionally an energy shift of 0.55 eV was applied in order to align the calculated spectra with experiment. The resulting best fit parameters are listed below.

_____________________________________________________________________________

Name: Ni2+_Oh_2p_XAS

Started: 2023-06-21 20:41:55.521607

Finished: 2023-06-21 20:41:55.747870

Element: Ni

Charge: 2+

Symmetry: Oh

Experiment: XAS

Edge: L2,3 (2p)

Temperature: 2.0 K

Magnetic Field: 6.0 T

Scale Factors:

Fk: 0.8

Gk: 0.8

ζ: 1.0

Hamiltonian Terms:

Atomic:

Initial Hamiltonian:

U(3d,3d): 5.0

F2(3d,3d): 9.7874

F4(3d,3d): 6.0782

ζ(3d): 0.0826

Final Hamiltonian:

U(3d,3d): 5.0

F2(3d,3d): 10.4054

F4(3d,3d): 6.4676

U(2p,3d): 0.0

F2(2p,3d): 6.177

G1(2p,3d): 4.6268

G3(2p,3d): 2.6322

ζ(3d): 0.1022

ζ(2p): 11.5084

Crystal Field:

Initial Hamiltonian:

10Dq(3d): 1.6

Final Hamiltonian:

10Dq(3d): 1.6

3d-Ligands Hybridization (LMCT):

Initial Hamiltonian:

Δ(3d,L1): 5.0

Veg(3d,L1): 2.4

Vt2g(3d,L1): 1.2

10Dq(L1): 0.0

Final Hamiltonian:

Δ(3d,L1): 5.0

Veg(3d,L1): 2.4

Vt2g(3d,L1): 1.2

10Dq(L1): 0.0

Magnetic Field:

Initial Hamiltonian:

Bx: 0.0

By: 0.0

Bz: 0.00034728

Final Hamiltonian:

Bx: 0.0

By: 0.0

Bz: 0.00034728

References

(1) *Crispy: v0.7.4*; 2019. https://doi.org/10.5281/zenodo.1008184 (accessed.

(2) Haverkort, M. W.; Zwierzycki, M.; Andersen, O. K. Multiplet ligand-field theory using Wannier orbitals. *Phys. Rev. B.* **2012**, *85* (16), 165113. DOI: 10.1103/PhysRevB.85.165113.

(3) van der Laan, G.; Thole, B. T. Strong magnetic x-ray dichroism in 2p absorption spectra of 3d transition-metal ions. *Phys. Rev. B.* **1991**, *43* (16), 13401-13411. DOI: 10.1103/PhysRevB.43.13401.

**
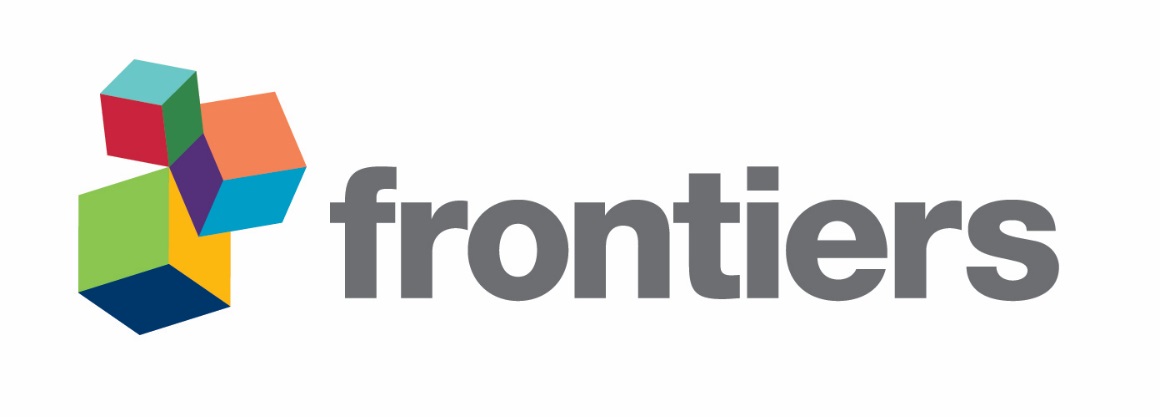
**

**Supplementary Figure 1.** The figure legends are required to have the same font as the main text, 12 point normal Times New Roman, single spaced. Please use a single paragraph for each legend and prepare the figures keeping in mind the PDF layout.

**
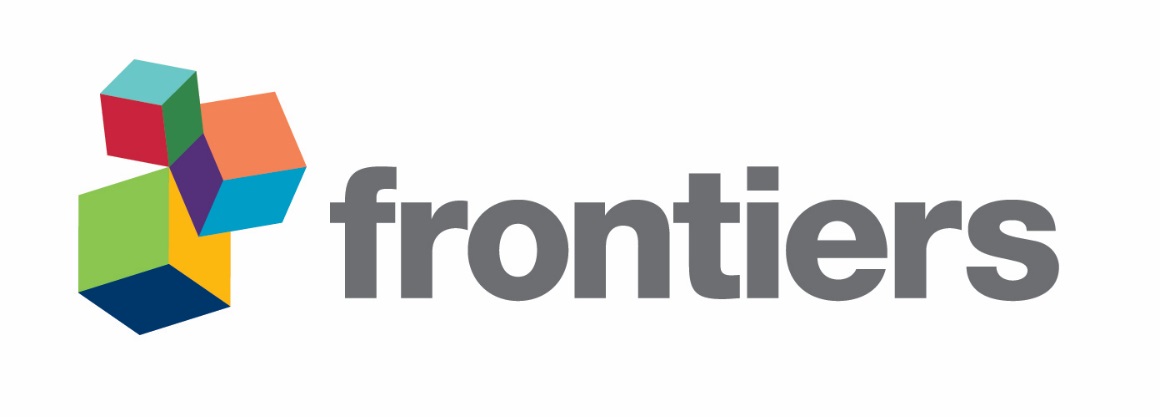
**

**Supplementary Figure 1.** The figure legends are required to have the same font as the main text, 12 point normal Times New Roman, single spaced. Please use a single paragraph for each legend and prepare the figures keeping in mind the PDF layout.

**
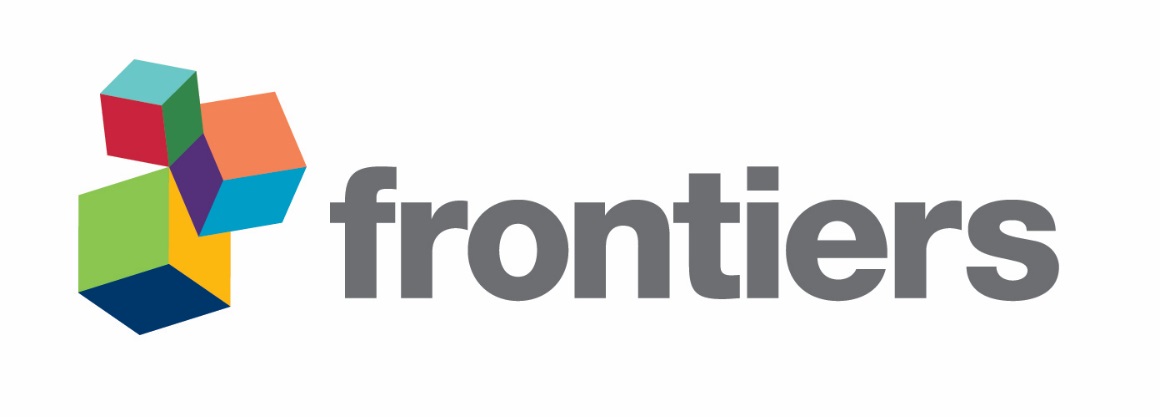
**

**Supplementary Figure 1.** The figure legends are required to have the same font as the main text, 12 point normal Times New Roman, single spaced. Please use a single paragraph for each legend and prepare the figures keeping in mind the PDF layout.

**
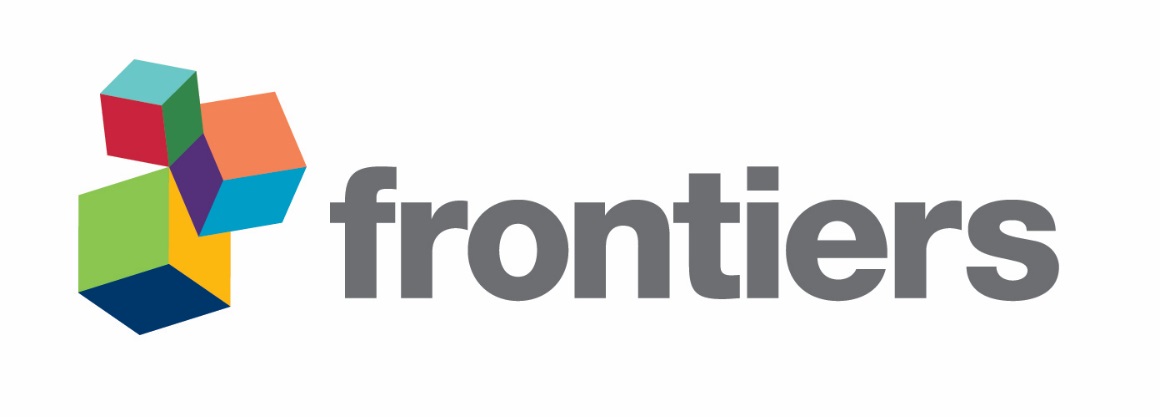
**

**Supplementary Figure 1.** The figure legends are required to have the same font as the main text, 12 point normal Times New Roman, single spaced. Please use a single paragraph for each legend and prepare the figures keeping in mind the PDF layout.

**
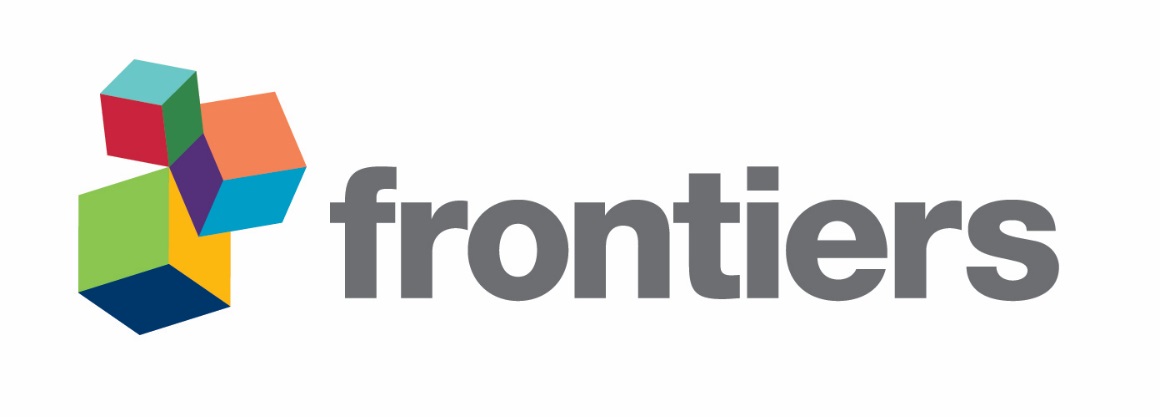
**

**Supplementary Figure 1.** The figure legends are required to have the same font as the main text, 12 point normal Times New Roman, single spaced. Please use a single paragraph for each legend and prepare the figures keeping in mind the PDF layout.

**
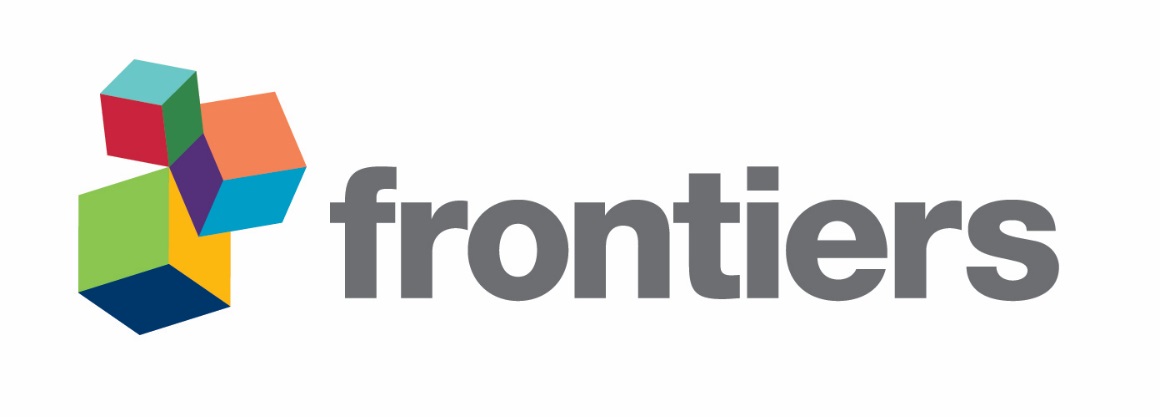
**

**Supplementary Figure 1.** The figure legends are required to have the same font as the main text, 12 point normal Times New Roman, single spaced. Please use a single paragraph for each legend and prepare the figures keeping in mind the PDF layout.

**
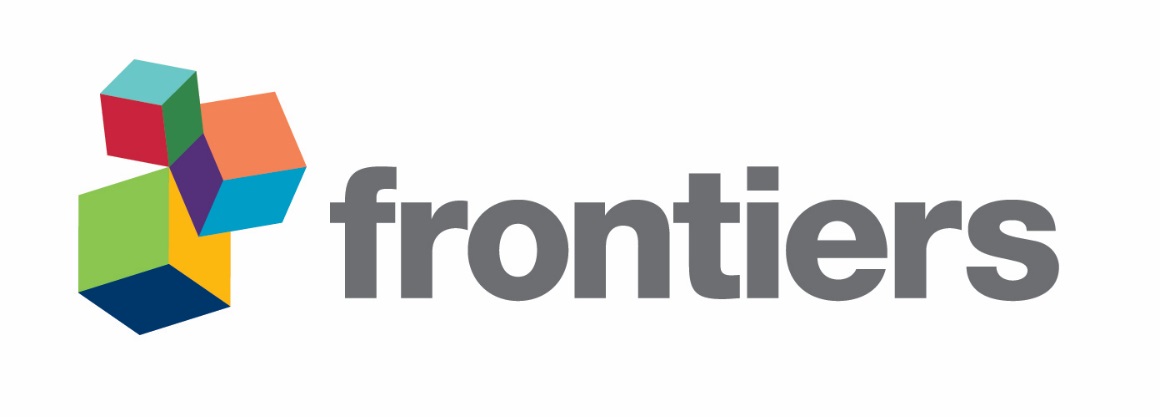
**

**Supplementary Figure 1.** The figure legends are required to have the same font as the main text, 12 point normal Times New Roman, single spaced. Please use a single paragraph for each legend and prepare the figures keeping in mind the PDF layout.

**
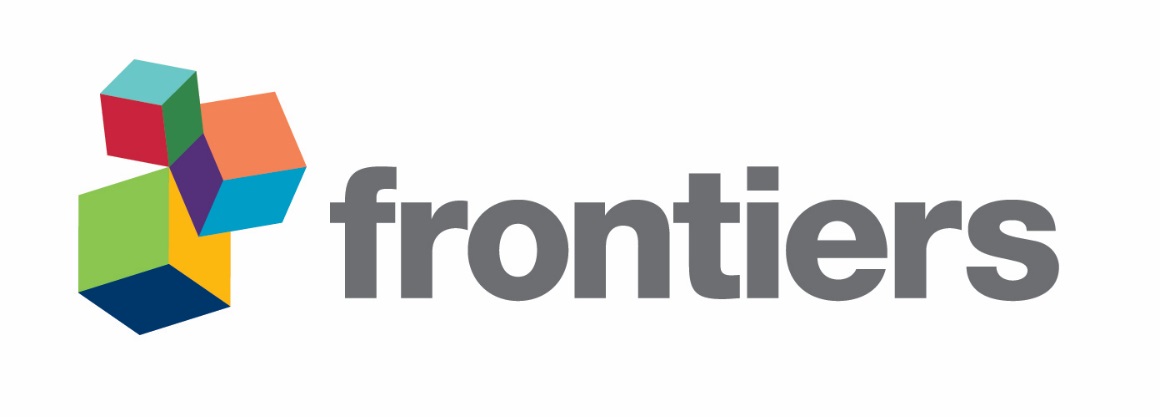
**

**Supplementary Figure 1.** The figure legends are required to have the same font as the main text, 12 point normal Times New Roman, single spaced. Please use a single paragraph for each legend and prepare the figures keeping in mind the PDF layout.

**
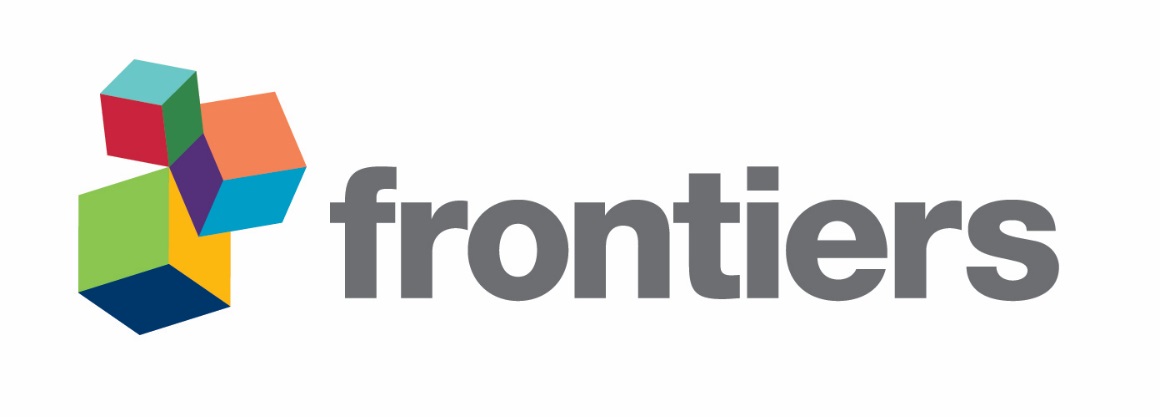
**

**Supplementary Figure 1.** The figure legends are required to have the same font as the main text, 12 point normal Times New Roman, single spaced. Please use a single paragraph for each legend and prepare the figures keeping in mind the PDF layout.

**
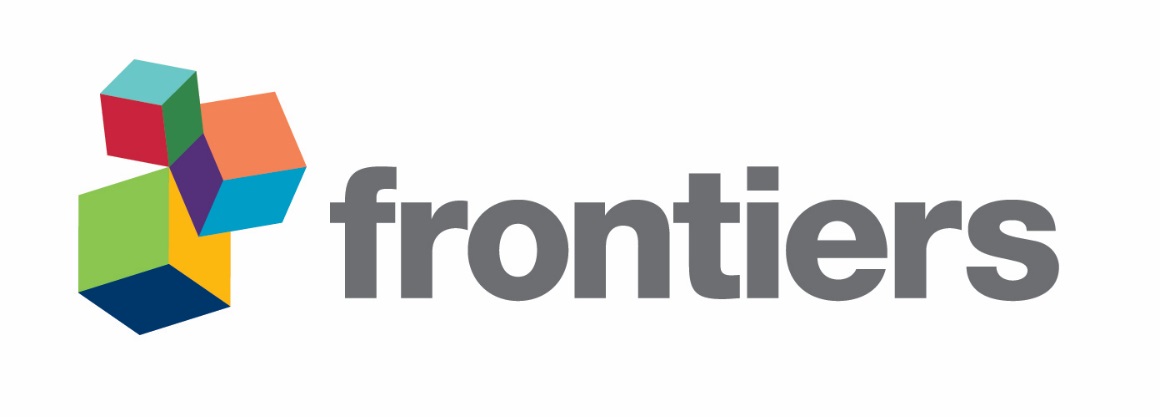
**

**Supplementary Figure 1.** The figure legends are required to have the same font as the main text, 12 point normal Times New Roman, single spaced. Please use a single paragraph for each legend and prepare the figures keeping in mind the PDF layout.

**
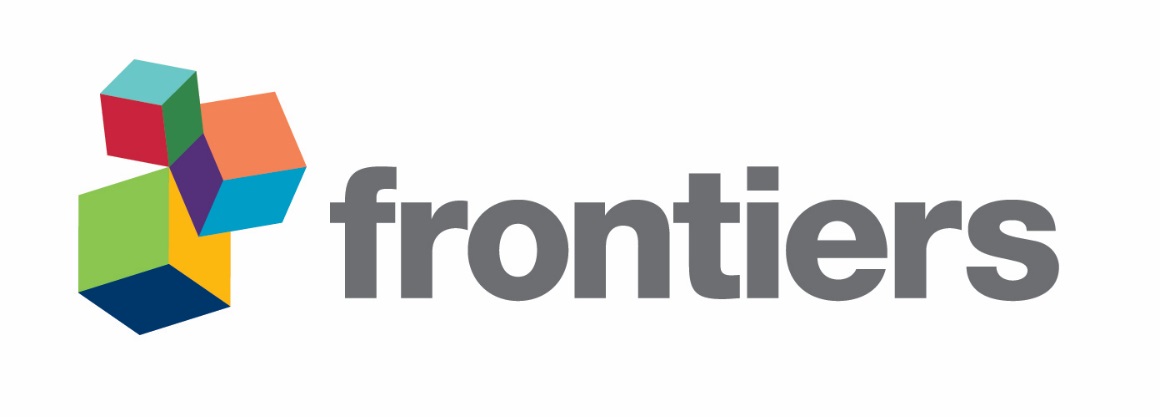
**

**Supplementary Figure 1.** The figure legends are required to have the same font as the main text, 12 point normal Times New Roman, single spaced. Please use a single paragraph for each legend and prepare the figures keeping in mind the PDF layout.

HAVERKORT, M. W., ZWIERZYCKI, M. & ANDERSEN, O. K. 2012. Multiplet ligand-field theory using Wannier orbitals. *Physical Review B,* 85**,** 165113.

RETEGAN, M. 2019. Crispy: v0.7.4.

VAN DER LAAN, G. & THOLE, B. T. 1991. Strong magnetic x-ray dichroism in 2p absorption spectra of 3d transition-metal ions. *Physical Review B,* 43**,** 13401-13411.
