# Supplementary material for: Metal-ligand interactions in a redox active ligand system. Electrochemistry and spectroscopy of [M(dipyvd)2]n+ (M=Zn, Ni, n=0, 1, 2)
Source: Front Chem. 2023 Nov 15;11:1295289. doi: 10.3389/fchem.2023.1295289 (PMC10684738; doi:10.3389/fchem.2023.1295289)

checkCIF/PLATON report

Structure factors have been supplied for datablock(s) sjsu10b_hex

THIS REPORT IS FOR GUIDANCE ONLY. IF USED AS PART OF A REVIEW PROCEDURE FOR PUBLICATION, IT SHOULD NOT REPLACE THE EXPERTISE OF AN EXPERIENCED CRYSTALLOGRAPHIC REFEREE.

No syntax errors found. CIF dictionary Interpreting this report

Datablock: sjsu10b_hex

```
Bond precision:   C-C = 0.0162 A          Wavelength=0.71073

Cell:              a=14.0975(8)      b=14.0975(8)      c=26.2314(17)
                   alpha=90          beta=90           gamma=120
Temperature:       100 K

                 Calculated                    Reported
Volume           4514.8(6)                     4514.8(6)
Space group      P 31 2 1                      P 31 2 1
Hall group       P 31 2"                       P 31 2"
Moiety formula   C30 H30 N12 O2 Zn             2(C15 H15 N6 O Zn0.5)
Sum formula      C30 H30 N12 O2 Zn             C30 H30 N12 O2 Zn
Mr               656.05                        656.03
Dx,g cm-3        1.448                         1.448
Z                6                             6
Mu (mm-1)        0.867                         0.867
F000             2040.0                        2040.0
F000'            2042.25
h,k,lmax         17,17,32                      17,17,32
Nref             6253[ 3512]                   6192
Tmin,Tmax
Tmin'

Correction method= Not given

Data completeness= 1.76/0.99        Theta(max)= 26.479

R(reflections)= 0.0636( 5312)                         wR2(reflections)=
                                                      0.1573( 6192)
S = 1.049                 Npar= 441
```

The following ALERTS were generated. Each ALERT has the format
test-name_ALERT_alert-type_alert-level.
Click on the hyperlinks for more details of the test.

[IMAGE] Alert level A

```
EXPT005_ALERT_1_A  _exptl_crystal_description is missing
            Crystal habit description.
            The following tests will not be performed.
            CRYSR_01
PLAT058_ALERT_1_A Maximum Transmission Factor Missing ...........          ?
PLAT059_ALERT_1_A Minimum Transmission Factor Missing ...........          ?
PLAT699_ALERT_1_A Missing _exptl_crystal_description Value .......     Please Do !
```

[IMAGE] Alert level B

```
PLAT234_ALERT_4_B Large Hirshfeld Difference C28A     --C29A     .       0.30 Ang.
PLAT341_ALERT_3_B Low Bond Precision on  C-C Bonds ..............    0.01621 Ang.
```

[IMAGE] Alert level C

```
ABSTY02_ALERT_1_C  An _exptl_absorpt_correction_type has been given without
            a literature citation. This should be contained in the
            _exptl_absorpt_process_details field.
            Absorption correction given as multi-scan
RINTA01_ALERT_3_C  The value of Rint is greater than 0.12
            Rint given   0.145
PLAT053_ALERT_1_C Minimum Crystal Dimension Missing (or Error) ...     Please Check
PLAT054_ALERT_1_C Medium  Crystal Dimension Missing (or Error) ...     Please Check
PLAT055_ALERT_1_C Maximum Crystal Dimension Missing (or Error) ...     Please Check
PLAT090_ALERT_3_C Poor Data / Parameter Ratio (Zmax > 18) ........       7.90 Note
PLAT213_ALERT_2_C Atom C30A            has ADP max/min Ratio .....        3.2 prolat
PLAT220_ALERT_2_C NonSolvent   Resd 1  C   Ueq(max)/Ueq(min) Range        3.9 Ratio
PLAT222_ALERT_3_C NonSolvent Resd 1  H   Uiso(max)/Uiso(min) Range        4.8 Ratio
PLAT230_ALERT_2_C Hirshfeld Test Diff for    C6A      --C7A      .        7.0 s.u.
PLAT234_ALERT_4_C Large Hirshfeld Difference N9A      --C16A     .       0.17 Ang.
PLAT234_ALERT_4_C Large Hirshfeld Difference C28A     --C29B     .       0.22 Ang.
PLAT234_ALERT_4_C Large Hirshfeld Difference C28A     --C30B     .       0.24 Ang.
PLAT234_ALERT_4_C Large Hirshfeld Difference C9A      --C10A     .       0.18 Ang.
PLAT234_ALERT_4_C Large Hirshfeld Difference C11A     --C12A     .       0.18 Ang.
PLAT234_ALERT_4_C Large Hirshfeld Difference C13A     --C14A     .       0.18 Ang.
PLAT241_ALERT_2_C High   'MainMol' Ueq as Compared to Neighbors of        N4A Check
PLAT242_ALERT_2_C Low    'MainMol' Ueq as Compared to Neighbors of       C28A Check
PLAT242_ALERT_2_C Low    'MainMol' Ueq as Compared to Neighbors of       C13A Check
PLAT250_ALERT_2_C Large U3/U1 Ratio for Average U(i,j) Tensor ....        2.2 Note
PLAT309_ALERT_2_C Single Bonded Oxygen (C-O > 1.3 Ang) ..........         O2A Check
PLAT309_ALERT_2_C Single Bonded Oxygen (C-O > 1.3 Ang) ..........         O2B Check
PLAT906_ALERT_3_C Large K Value in the Analysis of Variance ......      2.105 Check
```

[IMAGE] Alert level G

```
ABSMU01_ALERT_1_G  Calculation of _exptl_absorpt_correction_mu
                not performed for this radiation type.
PLAT003_ALERT_2_G Number of Uiso or Uij Restrained non-H Atoms ...          2 Report
PLAT020_ALERT_3_G The Value of Rint is Greater Than 0.12 .........      0.145 Report
PLAT042_ALERT_1_G Calc. and Reported Moiety Formula Strings Differ     Please Check
```

```
PLAT083_ALERT_2_G SHELXL Second Parameter in WGHT  Unusually Large       6.75 Why ?
PLAT168_ALERT_4_G The CIF-Embedded .res File Contains EXYZ Records          6 Report
PLAT171_ALERT_4_G The CIF-Embedded .res File Contains EADP Records          6 Report
PLAT186_ALERT_4_G The CIF-Embedded .res File Contains ISOR Records          1 Report
PLAT230_ALERT_2_G Hirshfeld Test Diff for    O2A      --C16A     .        5.2 s.u.
PLAT301_ALERT_3_G Main Residue  Disorder .............(Resd  1  )        40% Note
PLAT413_ALERT_2_G Short Inter XH3 .. XHn     H10A     ..H30B     .       2.03 Ang.
                                              x-y,1-y,5/3-z  =      4_566 Check
PLAT794_ALERT_5_G Tentative Bond Valency for Zn2       (II)      .       2.06 Info
PLAT860_ALERT_3_G Number of Least-Squares Restraints ............          12 Note
PLAT883_ALERT_1_G No Info/Value for _atom_sites_solution_primary .     Please Do !
PLAT910_ALERT_3_G Missing # of FCF Reflection(s) Below Theta(Min).          1 Note
PLAT912_ALERT_4_G Missing # of FCF Reflections Above STh/L=  0.600         27 Note
PLAT978_ALERT_2_G Number C-C Bonds with Positive Residual Density.          0 Info
```

---

4 **ALERT level A** = Most likely a serious problem - resolve or explain
2 **ALERT level B** = A potentially serious problem, consider carefully
23 **ALERT level C** = Check. Ensure it is not caused by an omission or oversight
17 **ALERT level G** = General information/check it is not something unexpected

11 ALERT type 1 CIF construction/syntax error, inconsistent or missing data
14 ALERT type 2 Indicator that the structure model may be wrong or deficient
9 ALERT type 3 Indicator that the structure quality may be low
11 ALERT type 4 Improvement, methodology, query or suggestion
1 ALERT type 5 Informative message, check

It is advisable to attempt to resolve as many as possible of the alerts in all categories. Often the minor alerts point to easily fixed oversights, errors and omissions in your CIF or refinement strategy, so attention to these fine details can be worthwhile. In order to resolve some of the more serious problems it may be necessary to carry out additional measurements or structure refinements. However, the purpose of your study may justify the reported deviations and the more serious of these should normally be commented upon in the discussion or experimental section of a paper or in the "special_details" fields of the CIF. checkCIF was carefully designed to identify outliers and unusual parameters, but every test has its limitations and alerts that are not important in a particular case may appear. Conversely, the absence of alerts does not guarantee there are no aspects of the results needing attention. It is up to the individual to critically assess their own results and, if necessary, seek expert advice.

Publication of your CIF in IUCr journals

A basic structural check has been run on your CIF. These basic checks will be run on all CIFs submitted for publication in IUCr journals (*Acta Crystallographica*, *Journal of Applied Crystallography*, *Journal of Synchrotron Radiation*); however, if you intend to submit to *Acta Crystallographica Section C* or *E* or *IUCrData*, you should make sure that full publication checks are run on the final version of your CIF prior to submission.

Publication of your CIF in other journals

Please refer to the *Notes for Authors* of the relevant journal for any special instructions relating to CIF submission.

Validation response form

Please find below a validation response form (VRF) that can be filled in and pasted into your CIF.

```
# start Validation Reply Form
_vrf_EXPT005_sjsu10b_hex
;
PROBLEM: _exptl_crystal_description is missing
RESPONSE: ...
;
_vrf_PLAT058_sjsu10b_hex
;
PROBLEM: Maximum Transmission Factor Missing ...........          ?
RESPONSE: ...
;
_vrf_PLAT059_sjsu10b_hex
;
PROBLEM: Minimum Transmission Factor Missing ...........          ?
RESPONSE: ...
;
_vrf_PLAT699_sjsu10b_hex
;
PROBLEM: Missing _exptl_crystal_description Value .......     Please Do !
RESPONSE: ...
;
# end Validation Reply Form
```

PLATON version of 20/01/2022; check.def file version of 19/01/2022

Datablock sjsu10b_hex - ellipsoid plot

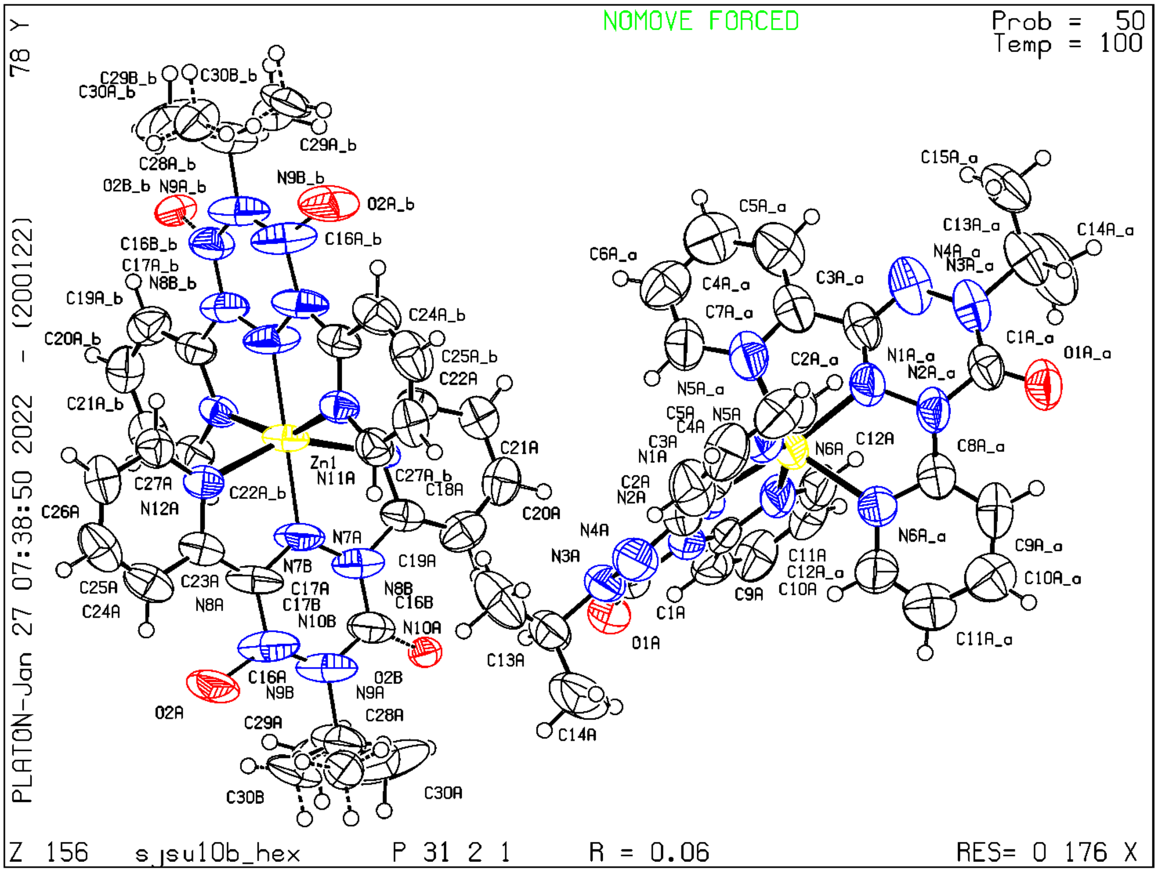

Supplement: Supplementary file 4 [file DataSheet3.PDF]
