# Supplementary material for: Metal-ligand interactions in a redox active ligand system. Electrochemistry and spectroscopy of [M(dipyvd)2]n+ (M=Zn, Ni, n=0, 1, 2)
Source: Front Chem. 2023 Nov 15;11:1295289. doi: 10.3389/fchem.2023.1295289 (PMC10684738; doi:10.3389/fchem.2023.1295289)
Supplement: Supplementary file 5 [file DataSheet1.PDF]

Sum rules

Importing libs

In [23]:
```
import numpy as np
import matplotlib.pylab as plt

import sys  # Needed for inserting the path of boreadan
sys.path.insert(0, '/Users/davidbrook/Documents/Research/Data/ALBA_XMCD_Mar2

from boreadan import load_data, summary_data, step_fun, find_pars_step_fun
```

In [24]:
```
# interactive graph inside the notebook
%matplotlib notebook
```

Defining the file to be opened

In [25]:
```
file_path = r"/Users/davidbrook/Documents/Research/Data/ALBA_XMCD_Mar2019/20
summary_data(file_path)
```

```
S4:  Energy = [835.0, 860.0],  Pol. = Cp,  magnet = -0.000,  T = 300.0 K
S5:  Energy = [835.0, 860.0],  Pol. = Cp,  magnet = -0.000,  T = 300.0 K
S6:  Energy = [835.0, 860.0],  Pol. = Cp,  magnet = -0.000,  T = 300.0 K
S7:  Energy = [840.0, 870.0],  Pol. = Cp,  magnet = -0.000,  T = 300.0 K
S8:  Energy = [845.0, 875.0],  Pol. = Cp,  magnet = -0.000,  T = 300.0 K
S9:  Energy = [845.0, 875.0],  Pol. = Cp,  magnet = -0.000,  T = 300.0 K
S10:  Energy = [845.0, 875.0],  Pol. = Cp,  magnet = -0.000,  T = 300.0 K
S11:  Energy = [845.0, 875.0],  Pol. = Cp,  magnet = -0.000,  T = 300.0 K
S12:  Energy = [845.0, 875.0],  Pol. = Cp,  magnet = -0.000,  T = 300.0 K
S13:  Energy = [845.0, 875.0],  Pol. = Cp,  magnet = -0.000,  T = 300.0 K
S14:  Energy = [390.0, 440.0],  Pol. = Cp,  magnet = -0.000,  T = 300.0 K
S15:  Energy = [390.0, 440.0],  Pol. = Cp,  magnet = -0.000,  T = 300.0 K
S16:  Energy = [390.0, 440.0],  Pol. = Cp,  magnet = -0.000,  T = 300.0 K
S18:  Energy = [845.0, 860.0],  Pol. = Cp,  magnet = -0.000,  T = 300.0 K
S24:  Energy = [390.0, 430.0],  Pol. = Cp,  magnet = -0.000,  T = 300.0 K
S25:  Energy = [390.0, 430.0],  Pol. = Cp,  magnet = -0.000,  T = 300.0 K
S26:  Energy = [390.0, 440.0],  Pol. = Cp,  magnet = -6.000,  T = 300.0 K
S27:  Energy = [390.0, 440.0],  Pol. = Cm,  magnet = -6.000,  T = 300.0 K
S28:  Energy = [390.0, 440.0],  Pol. = Cm,  magnet = -6.000,  T = 300.0 K
S29:  Energy = [390.0, 440.0],  Pol. = Cp,  magnet = -6.000,  T = 300.0 K
S30:  Energy = [835.0, 890.0],  Pol. = Cp,  magnet = -6.000,  T = 300.0 K
S31:  Energy = [840.0, 895.0],  Pol. = Cm,  magnet = -6.000,  T = 300.0 K
S32:  Energy = [270.0, 330.0],  Pol. = Cp,  magnet = -6.000,  T = 300.0 K
S33:  Energy = [270.0, 330.0],  Pol. = Cp,  magnet = -6.000,  T = 300.0 K
S34:  Energy = [270.0, 330.0],  Pol. = Cp,  magnet = -6.000,  T = 300.0 K
S35:  Energy = [270.0, 330.0],  Pol. = Cm,  magnet = -6.000,  T = 300.0 K
S36:  Energy = [270.0, 330.0],  Pol. = Cp,  magnet = -6.000,  T = 300.0 K
S37:  Energy = [270.0, 330.0],  Pol. = Cm,  magnet = -6.000,  T = 300.0 K
S38:  Energy = [270.0, 330.0],  Pol. = Cm,  magnet = -6.000,  T = 300.0 K
```

S39: Energy = [270.0, 330.0], Pol. = Cp, magnet = -6.001, T = 300.0 K
S40: Energy = [270.0, 330.0], Pol. = Cm, magnet = -6.001, T = 300.0 K
S41: Energy = [270.0, 330.0], Pol. = Cp, magnet = -6.000, T = 300.0 K
S42: Energy = [270.0, 330.0], Pol. = Cp, magnet = -6.001, T = 300.0 K
S43: Energy = [270.0, 330.0], Pol. = Cm, magnet = -6.001, T = 300.0 K
S44: Energy = [390.0, 430.0], Pol. = Cp, magnet = -6.001, T = 300.0 K
S45: Energy = [390.0, 430.0], Pol. = Cp, magnet = -6.001, T = 300.0 K
S46: Energy = [390.0, 440.0], Pol. = Cp, magnet = -6.001, T = 300.0 K
S47: Energy = [390.0, 440.0], Pol. = Cm, magnet = -6.001, T = 300.0 K
S48: Energy = [390.0, 440.0], Pol. = Cm, magnet = -6.001, T = 300.0 K
S49: Energy = [390.0, 440.0], Pol. = Cp, magnet = -6.001, T = 300.0 K
S50: Energy = [390.0, 440.0], Pol. = Cm, magnet = -6.001, T = 300.0 K
S51: Energy = [390.0, 440.0], Pol. = Cp, magnet = -6.001, T = 300.0 K
S52: Energy = [390.0, 440.0], Pol. = Cp, magnet = -6.001, T = 300.0 K
S53: Energy = [390.0, 440.0], Pol. = Cm, magnet = -6.001, T = 300.0 K
S54: Energy = [390.0, 440.0], Pol. = Cp, magnet = -6.001, T = 300.0 K
S55: Energy = [390.0, 440.0], Pol. = Cm, magnet = -6.001, T = 300.0 K
S56: Energy = [390.0, 440.0], Pol. = Cm, magnet = -6.001, T = 300.0 K
S57: Energy = [390.0, 440.0], Pol. = Cp, magnet = -6.001, T = 300.0 K
S58: Energy = [390.0, 440.0], Pol. = Cm, magnet = -6.001, T = 300.0 K
S59: Energy = [390.0, 440.0], Pol. = Cp, magnet = -6.001, T = 300.0 K
S60: Energy = [390.0, 440.0], Pol. = Cp, magnet = -6.001, T = 300.0 K
S61: Energy = [390.0, 440.0], Pol. = Cm, magnet = -6.001, T = 300.0 K
S62: Energy = [845.0, 860.0], Pol. = Cm, magnet = 6.000, T = 300.0 K
S63: Energy = [845.0, 860.0], Pol. = Cm, magnet = 6.000, T = 300.0 K
S64: Energy = [845.0, 860.0], Pol. = Cm, magnet = 6.000, T = 300.0 K
S65: Energy = [845.0, 860.0], Pol. = Cm, magnet = 6.000, T = 300.0 K
S66: Energy = [845.0, 860.0], Pol. = Cm, magnet = 6.000, T = 300.0 K
S67: Energy = [845.0, 860.0], Pol. = Cm, magnet = 6.000, T = 300.0 K
S70: Energy = [845.0, 860.0], Pol. = Cm, magnet = 6.001, T = 300.0 K
S71: Energy = [390.0, 430.0], Pol. = Cm, magnet = 6.001, T = 300.0 K
S72: Energy = [395.0, 405.0], Pol. = Cm, magnet = 6.001, T = 300.0 K
S75: Energy = [840.0, 895.0], Pol. = Cp, magnet = 6.001, T = 300.0 K
S76: Energy = [840.0, 895.0], Pol. = Cm, magnet = 6.001, T = 300.0 K
S77: Energy = [840.0, 895.0], Pol. = Cm, magnet = 6.001, T = 300.0 K
S78: Energy = [840.0, 895.0], Pol. = Cp, magnet = 6.001, T = 300.0 K
S79: Energy = [840.0, 895.0], Pol. = Cm, magnet = 6.001, T = 300.0 K
S80: Energy = [840.0, 895.0], Pol. = Cp, magnet = 6.001, T = 300.0 K
S81: Energy = [840.0, 895.0], Pol. = Cp, magnet = 6.001, T = 300.0 K
S82: Energy = [840.0, 895.0], Pol. = Cm, magnet = 6.001, T = 300.0 K
S83: Energy = [390.0, 440.0], Pol. = Cp, magnet = 6.001, T = 300.0 K
S84: Energy = [390.0, 440.0], Pol. = Cm, magnet = 6.001, T = 300.0 K
S86: Energy = [845.0, 860.0], Pol. = Cm, magnet = 6.001, T = 300.0 K
S87: Energy = [845.0, 860.0], Pol. = Cm, magnet = 6.001, T = 300.0 K
S88: Energy = [390.0, 430.0], Pol. = Cm, magnet = 6.001, T = 300.0 K
S89: Energy = [840.0, 895.0], Pol. = Cp, magnet = 6.001, T = 300.0 K
S90: Energy = [840.0, 895.0], Pol. = Cm, magnet = 6.001, T = 300.0 K
S91: Energy = [840.0, 895.0], Pol. = Cm, magnet = 6.001, T = 300.0 K
S92: Energy = [840.0, 895.0], Pol. = Cp, magnet = 6.001, T = 300.0 K
S93: Energy = [840.0, 895.0], Pol. = Cm, magnet = 6.001, T = 300.0 K
S94: Energy = [840.0, 895.0], Pol. = Cp, magnet = 6.001, T = 300.0 K
S95: Energy = [840.0, 895.0], Pol. = Cp, magnet = 6.001, T = 300.0 K
S96: Energy = [840.0, 895.0], Pol. = Cm, magnet = 6.001, T = 300.0 K
S97: Energy = [390.0, 440.0], Pol. = Cp, magnet = 6.001, T = 300.0 K
S98: Energy = [390.0, 440.0], Pol. = Cm, magnet = 6.001, T = 300.0 K
S99: Energy = [390.0, 440.0], Pol. = Cm, magnet = 6.001, T = 300.0 K
S100: Energy = [390.0, 440.0], Pol. = Cp, magnet = 6.001, T = 300.0 K

```
S101:  Energy = [390.0, 440.0],  Pol. = Cm,  magnet = 6.001,  T = 300.0 K
S102:  Energy = [390.0, 440.0],  Pol. = Cp,  magnet = 6.001,  T = 300.0 K
S103:  Energy = [390.0, 440.0],  Pol. = Cp,  magnet = 6.001,  T = 300.0 K
S104:  Energy = [390.0, 440.0],  Pol. = Cm,  magnet = 6.001,  T = 300.0 K
S105:  Energy = [390.0, 440.0],  Pol. = Cp,  magnet = 6.001,  T = 300.0 K
S106:  Energy = [390.0, 440.0],  Pol. = Cm,  magnet = 6.001,  T = 300.0 K
S107:  Energy = [390.0, 440.0],  Pol. = Cm,  magnet = 6.001,  T = 300.0 K
S108:  Energy = [390.0, 440.0],  Pol. = Cp,  magnet = 6.001,  T = 300.0 K
S109:  Energy = [390.0, 440.0],  Pol. = Cm,  magnet = 6.001,  T = 300.0 K
S110:  Energy = [390.0, 440.0],  Pol. = Cp,  magnet = 6.001,  T = 300.0 K
S111:  Energy = [390.0, 440.0],  Pol. = Cp,  magnet = 6.001,  T = 300.0 K
S112:  Energy = [390.0, 440.0],  Pol. = Cm,  magnet = 6.001,  T = 300.0 K
S113:  Energy = [390.0, 440.0],  Pol. = Cp,  magnet = 6.001,  T = 300.0 K
S114:  Energy = [390.0, 440.0],  Pol. = Cm,  magnet = 6.001,  T = 300.0 K
S115:  Energy = [390.0, 440.0],  Pol. = Cm,  magnet = 6.001,  T = 300.0 K
S116:  Energy = [390.0, 440.0],  Pol. = Cp,  magnet = 6.001,  T = 300.0 K
S117:  Energy = [390.0, 440.0],  Pol. = Cm,  magnet = 6.001,  T = 300.0 K
S118:  Energy = [390.0, 440.0],  Pol. = Cp,  magnet = 6.001,  T = 300.0 K
S119:  Energy = [390.0, 440.0],  Pol. = Cp,  magnet = 6.001,  T = 300.0 K
S120:  Energy = [390.0, 440.0],  Pol. = Cm,  magnet = 6.001,  T = 300.0 K
S121:  Energy = [390.0, 440.0],  Pol. = Cp,  magnet = 6.001,  T = 300.0 K
S122:  Energy = [390.0, 440.0],  Pol. = Cm,  magnet = 6.001,  T = 300.0 K
S123:  Energy = [390.0, 440.0],  Pol. = Cm,  magnet = 6.001,  T = 300.0 K
S124:  Energy = [390.0, 440.0],  Pol. = Cp,  magnet = 6.001,  T = 300.0 K
S125:  Energy = [390.0, 440.0],  Pol. = Cm,  magnet = 6.001,  T = 300.0 K
S126:  Energy = [390.0, 440.0],  Pol. = Cp,  magnet = 6.001,  T = 300.0 K
S127:  Energy = [390.0, 440.0],  Pol. = Cp,  magnet = 6.001,  T = 300.0 K
S128:  Energy = [390.0, 440.0],  Pol. = Cm,  magnet = 6.001,  T = 300.0 K
S129:  Energy = [840.0, 895.0],  Pol. = Cp,  magnet = -6.000,  T = 300.0 K
S130:  Energy = [840.0, 895.0],  Pol. = Cm,  magnet = -6.000,  T = 300.0 K
S131:  Energy = [840.0, 895.0],  Pol. = Cm,  magnet = -6.001,  T = 300.0 K
S132:  Energy = [840.0, 895.0],  Pol. = Cp,  magnet = -6.000,  T = 300.0 K
S133:  Energy = [840.0, 895.0],  Pol. = Cm,  magnet = -6.000,  T = 300.0 K
S134:  Energy = [840.0, 895.0],  Pol. = Cp,  magnet = -6.000,  T = 300.0 K
S135:  Energy = [840.0, 895.0],  Pol. = Cp,  magnet = -6.000,  T = 300.0 K
S136:  Energy = [840.0, 895.0],  Pol. = Cm,  magnet = -6.000,  T = 300.0 K
S137:  Energy = [390.0, 440.0],  Pol. = Cp,  magnet = -6.001,  T = 300.0 K
S138:  Energy = [390.0, 440.0],  Pol. = Cm,  magnet = -6.001,  T = 300.0 K
S139:  Energy = [390.0, 440.0],  Pol. = Cm,  magnet = -6.001,  T = 300.0 K
S140:  Energy = [390.0, 440.0],  Pol. = Cp,  magnet = -6.001,  T = 300.0 K
S141:  Energy = [390.0, 440.0],  Pol. = Cm,  magnet = -6.001,  T = 300.0 K
S142:  Energy = [390.0, 440.0],  Pol. = Cp,  magnet = -6.001,  T = 300.0 K
S143:  Energy = [390.0, 440.0],  Pol. = Cp,  magnet = -6.001,  T = 300.0 K
S144:  Energy = [390.0, 440.0],  Pol. = Cm,  magnet = -6.001,  T = 300.0 K
S145:  Energy = [390.0, 440.0],  Pol. = Cp,  magnet = -6.001,  T = 300.0 K
S146:  Energy = [390.0, 440.0],  Pol. = Cm,  magnet = -6.001,  T = 300.0 K
S147:  Energy = [390.0, 440.0],  Pol. = Cm,  magnet = -6.001,  T = 300.0 K
S148:  Energy = [390.0, 440.0],  Pol. = Cp,  magnet = -6.001,  T = 300.0 K
S149:  Energy = [390.0, 440.0],  Pol. = Cm,  magnet = -6.001,  T = 300.0 K
S150:  Energy = [390.0, 440.0],  Pol. = Cp,  magnet = -6.001,  T = 300.0 K
S151:  Energy = [390.0, 440.0],  Pol. = Cp,  magnet = -6.001,  T = 300.0 K
S152:  Energy = [390.0, 440.0],  Pol. = Cm,  magnet = -6.001,  T = 300.0 K
S153:  Energy = [390.0, 440.0],  Pol. = Cp,  magnet = -6.001,  T = 300.0 K
S154:  Energy = [390.0, 440.0],  Pol. = Cm,  magnet = -6.001,  T = 300.0 K
S155:  Energy = [390.0, 440.0],  Pol. = Cm,  magnet = -6.001,  T = 300.0 K
S156:  Energy = [390.0, 440.0],  Pol. = Cp,  magnet = -6.001,  T = 300.0 K
S157:  Energy = [390.0, 440.0],  Pol. = Cm,  magnet = -6.001,  T = 300.0 K
```

```
S158:  Energy = [390.0, 440.0],  Pol. = Cp,  magnet = -6.001,  T = 300.0 K
S159:  Energy = [390.0, 440.0],  Pol. = Cp,  magnet = -6.001,  T = 300.0 K
S160:  Energy = [390.0, 440.0],  Pol. = Cm,  magnet = -6.001,  T = 300.0 K
S161:  Energy = [390.0, 440.0],  Pol. = Cp,  magnet = -6.001,  T = 300.0 K
S162:  Energy = [390.0, 440.0],  Pol. = Cm,  magnet = -6.001,  T = 300.0 K
S163:  Energy = [390.0, 440.0],  Pol. = Cm,  magnet = -6.001,  T = 300.0 K
S164:  Energy = [390.0, 440.0],  Pol. = Cp,  magnet = -6.001,  T = 300.0 K
S165:  Energy = [390.0, 440.0],  Pol. = Cm,  magnet = -6.001,  T = 300.0 K
S166:  Energy = [390.0, 440.0],  Pol. = Cp,  magnet = -6.001,  T = 300.0 K
S167:  Energy = [390.0, 440.0],  Pol. = Cp,  magnet = -6.001,  T = 300.0 K
S168:  Energy = [390.0, 440.0],  Pol. = Cm,  magnet = -6.001,  T = 300.0 K
S169:  Energy = [840.0, 895.0],  Pol. = Cm,  magnet = -6.001,  T = 300.0 K
S170:  Energy = [840.0, 895.0],  Pol. = Cp,  magnet = -6.001,  T = 300.0 K
S173:  Energy = [845.0, 860.0],  Pol. = Cp,  magnet = -6.001,  T = 2.3 K
S174:  Energy = [845.0, 860.0],  Pol. = Cm,  magnet = -6.001,  T = 2.4 K
S177:  Energy = [840.0, 895.0],  Pol. = Cp,  magnet = -6.001,  T = 2.3 K
S178:  Energy = [840.0, 895.0],  Pol. = Cm,  magnet = -6.001,  T = 2.2 K
S179:  Energy = [840.0, 895.0],  Pol. = Cm,  magnet = -6.001,  T = 2.1 K
S180:  Energy = [840.0, 895.0],  Pol. = Cp,  magnet = -6.001,  T = 2.2 K
S181:  Energy = [840.0, 895.0],  Pol. = Cm,  magnet = -6.001,  T = 2.2 K
S182:  Energy = [840.0, 895.0],  Pol. = Cp,  magnet = -6.001,  T = 2.2 K
S183:  Energy = [840.0, 895.0],  Pol. = Cp,  magnet = -6.001,  T = 2.2 K
S184:  Energy = [840.0, 895.0],  Pol. = Cm,  magnet = -6.001,  T = 2.1 K
S185:  Energy = [390.0, 440.0],  Pol. = Cp,  magnet = -6.001,  T = 2.1 K
S186:  Energy = [390.0, 440.0],  Pol. = Cm,  magnet = -6.001,  T = 2.1 K
S187:  Energy = [390.0, 440.0],  Pol. = Cm,  magnet = -6.001,  T = 2.1 K
S188:  Energy = [390.0, 440.0],  Pol. = Cp,  magnet = -6.001,  T = 2.1 K
S189:  Energy = [390.0, 440.0],  Pol. = Cm,  magnet = -6.001,  T = 2.1 K
S190:  Energy = [390.0, 440.0],  Pol. = Cp,  magnet = -6.001,  T = 2.1 K
S191:  Energy = [390.0, 440.0],  Pol. = Cp,  magnet = -6.001,  T = 2.1 K
S192:  Energy = [390.0, 440.0],  Pol. = Cm,  magnet = -6.001,  T = 2.1 K
S194:  Energy = [840.0, 895.0],  Pol. = Cp,  magnet = -6.001,  T = 25.0 K
S195:  Energy = [840.0, 895.0],  Pol. = Cm,  magnet = -6.001,  T = 25.0 K
S196:  Energy = [840.0, 895.0],  Pol. = Cm,  magnet = -6.001,  T = 25.0 K
S197:  Energy = [840.0, 895.0],  Pol. = Cp,  magnet = -6.001,  T = 25.0 K
S198:  Energy = [840.0, 895.0],  Pol. = Cm,  magnet = -6.001,  T = 25.0 K
S199:  Energy = [840.0, 895.0],  Pol. = Cp,  magnet = -6.001,  T = 25.0 K
S200:  Energy = [840.0, 895.0],  Pol. = Cp,  magnet = -6.001,  T = 25.0 K
S201:  Energy = [840.0, 895.0],  Pol. = Cm,  magnet = -6.001,  T = 25.0 K
S202:  Energy = [700.0, 732.0],  Pol. = Cm,  magnet = -6.001,  T = 2.9 K
S203:  Energy = [680.0, 732.0],  Pol. = Cm,  magnet = -6.001,  T = 2.9 K
S211:  Energy = [680.0, 745.0],  Pol. = Cp,  magnet = -6.000,  T = 2.6 K
S212:  Energy = [680.0, 745.0],  Pol. = Cp,  magnet = -6.000,  T = 2.7 K
S213:  Energy = [680.0, 745.0],  Pol. = Cm,  magnet = -6.001,  T = 2.7 K
S214:  Energy = [680.0, 745.0],  Pol. = Cm,  magnet = -6.001,  T = 2.7 K
S215:  Energy = [680.0, 745.0],  Pol. = Cp,  magnet = -6.001,  T = 2.4 K
S216:  Energy = [680.0, 745.0],  Pol. = Cm,  magnet = -6.001,  T = 2.3 K
S217:  Energy = [680.0, 745.0],  Pol. = Cp,  magnet = -6.001,  T = 2.1 K
S218:  Energy = [680.0, 745.0],  Pol. = Cp,  magnet = -6.000,  T = 2.1 K
S219:  Energy = [680.0, 745.0],  Pol. = Cm,  magnet = -6.000,  T = 2.1 K
S220:  Energy = [390.0, 440.0],  Pol. = Cp,  magnet = -6.000,  T = 2.1 K
S221:  Energy = [390.0, 440.0],  Pol. = Cp,  magnet = -6.001,  T = 2.1 K
S222:  Energy = [390.0, 440.0],  Pol. = Cm,  magnet = -6.000,  T = 2.1 K
S223:  Energy = [700.0, 732.0],  Pol. = Cp,  magnet = -0.000,  T = 300.0 K
S228:  Energy = [770.0, 810.0],  Pol. = Cp,  magnet = -6.000,  T = 1.9 K
S233:  Energy = [680.0, 745.0],  Pol. = Cp,  magnet = -6.000,  T = 2.3 K
S234:  Energy = [680.0, 745.0],  Pol. = Cp,  magnet = -6.000,  T = 2.2 K
```

S235: Energy = [680.0, 745.0], Pol. = Cm, magnet = -6.000, T = 2.2 K
S236: Energy = [680.0, 745.0], Pol. = Cm, magnet = -6.000, T = 2.2 K
S237: Energy = [680.0, 745.0], Pol. = Cp, magnet = -6.000, T = 2.2 K
S238: Energy = [680.0, 745.0], Pol. = Cm, magnet = -6.000, T = 2.1 K
S239: Energy = [680.0, 745.0], Pol. = Cp, magnet = -6.000, T = 2.1 K
S240: Energy = [680.0, 745.0], Pol. = Cp, magnet = -6.000, T = 2.1 K
S241: Energy = [680.0, 745.0], Pol. = Cm, magnet = -6.000, T = 2.1 K
S242: Energy = [390.0, 440.0], Pol. = Cp, magnet = -6.000, T = 2.1 K
S243: Energy = [390.0, 440.0], Pol. = Cp, magnet = -6.000, T = 2.1 K
S244: Energy = [390.0, 440.0], Pol. = Cm, magnet = -6.000, T = 2.1 K
S245: Energy = [390.0, 440.0], Pol. = Cm, magnet = -6.000, T = 2.1 K
S246: Energy = [390.0, 440.0], Pol. = Cp, magnet = -6.000, T = 2.0 K
S247: Energy = [390.0, 440.0], Pol. = Cm, magnet = -6.000, T = 2.0 K
S248: Energy = [390.0, 440.0], Pol. = Cp, magnet = -6.000, T = 1.9 K
S249: Energy = [390.0, 440.0], Pol. = Cp, magnet = -6.000, T = 1.9 K
S250: Energy = [390.0, 440.0], Pol. = Cm, magnet = -6.000, T = 1.9 K
S251: Energy = [680.0, 745.0], Pol. = Cp, magnet = -6.000, T = 1.9 K
S252: Energy = [680.0, 745.0], Pol. = Cp, magnet = -6.000, T = 1.8 K
S253: Energy = [680.0, 745.0], Pol. = Cm, magnet = -6.000, T = 1.8 K
S254: Energy = [680.0, 745.0], Pol. = Cm, magnet = -6.000, T = 1.8 K
S255: Energy = [680.0, 745.0], Pol. = Cp, magnet = -6.000, T = 1.8 K
S256: Energy = [680.0, 745.0], Pol. = Cm, magnet = -6.000, T = 1.8 K
S257: Energy = [680.0, 745.0], Pol. = Cp, magnet = -6.000, T = 1.8 K
S258: Energy = [680.0, 745.0], Pol. = Cp, magnet = -6.000, T = 1.8 K
S259: Energy = [680.0, 745.0], Pol. = Cm, magnet = -6.000, T = 1.8 K
S260: Energy = [390.0, 440.0], Pol. = Cp, magnet = -6.000, T = 1.8 K
S261: Energy = [390.0, 440.0], Pol. = Cp, magnet = -6.000, T = 1.8 K
S262: Energy = [390.0, 440.0], Pol. = Cm, magnet = -6.000, T = 1.8 K
S263: Energy = [390.0, 440.0], Pol. = Cm, magnet = -6.000, T = 1.8 K
S264: Energy = [390.0, 440.0], Pol. = Cp, magnet = -6.000, T = 1.8 K
S265: Energy = [390.0, 440.0], Pol. = Cm, magnet = -6.000, T = 1.8 K
S266: Energy = [390.0, 440.0], Pol. = Cp, magnet = -6.000, T = 1.8 K
S267: Energy = [390.0, 440.0], Pol. = Cp, magnet = -6.000, T = 1.8 K
S268: Energy = [390.0, 440.0], Pol. = Cm, magnet = -6.000, T = 1.8 K
S269: Energy = [770.0, 820.0], Pol. = Cp, magnet = -6.000, T = 1.8 K
S270: Energy = [770.0, 820.0], Pol. = Cp, magnet = -6.000, T = 1.8 K
S271: Energy = [770.0, 820.0], Pol. = Cm, magnet = -6.000, T = 1.8 K
S272: Energy = [770.0, 820.0], Pol. = Cm, magnet = -6.000, T = 1.8 K
S273: Energy = [770.0, 820.0], Pol. = Cp, magnet = -6.000, T = 1.8 K
S274: Energy = [770.0, 820.0], Pol. = Cm, magnet = -6.000, T = 1.8 K
S275: Energy = [770.0, 820.0], Pol. = Cp, magnet = -6.000, T = 1.8 K
S276: Energy = [770.0, 820.0], Pol. = Cp, magnet = -6.000, T = 1.8 K
S277: Energy = [770.0, 820.0], Pol. = Cm, magnet = -6.000, T = 1.8 K
S278: Energy = [390.0, 440.0], Pol. = Cp, magnet = -6.000, T = 1.8 K
S279: Energy = [390.0, 440.0], Pol. = Cp, magnet = -6.000, T = 1.8 K
S280: Energy = [390.0, 440.0], Pol. = Cm, magnet = -6.000, T = 1.8 K
S281: Energy = [390.0, 440.0], Pol. = Cm, magnet = -6.000, T = 1.8 K
S282: Energy = [390.0, 440.0], Pol. = Cp, magnet = -6.000, T = 1.8 K
S283: Energy = [390.0, 440.0], Pol. = Cm, magnet = -6.000, T = 1.8 K
S284: Energy = [390.0, 440.0], Pol. = Cp, magnet = -6.000, T = 1.8 K
S285: Energy = [390.0, 440.0], Pol. = Cp, magnet = -6.000, T = 1.8 K
S286: Energy = [390.0, 440.0], Pol. = Cm, magnet = -6.000, T = 1.8 K
S287: Energy = [770.0, 820.0], Pol. = Cp, magnet = -6.000, T = 1.8 K
S288: Energy = [770.0, 820.0], Pol. = Cp, magnet = -6.000, T = 1.8 K
S289: Energy = [770.0, 820.0], Pol. = Cm, magnet = -6.000, T = 1.8 K
S290: Energy = [770.0, 820.0], Pol. = Cm, magnet = -6.000, T = 1.8 K
S291: Energy = [770.0, 820.0], Pol. = Cp, magnet = -6.000, T = 1.8 K

```
S292:  Energy = [770.0, 820.0],  Pol. = Cm,  magnet = -6.000,  T = 1.8 K
S293:  Energy = [770.0, 820.0],  Pol. = Cp,  magnet = -6.000,  T = 1.8 K
S294:  Energy = [770.0, 820.0],  Pol. = Cp,  magnet = -6.000,  T = 1.8 K
S295:  Energy = [770.0, 820.0],  Pol. = Cm,  magnet = -6.000,  T = 1.8 K
S296:  Energy = [390.0, 440.0],  Pol. = Cp,  magnet = -6.000,  T = 1.8 K
S297:  Energy = [390.0, 440.0],  Pol. = Cp,  magnet = -6.000,  T = 1.8 K
S298:  Energy = [390.0, 440.0],  Pol. = Cm,  magnet = -6.000,  T = 1.8 K
S299:  Energy = [390.0, 440.0],  Pol. = Cm,  magnet = -6.000,  T = 1.8 K
S300:  Energy = [390.0, 440.0],  Pol. = Cp,  magnet = -6.000,  T = 1.8 K
S301:  Energy = [390.0, 440.0],  Pol. = Cm,  magnet = -6.000,  T = 1.8 K
S302:  Energy = [390.0, 440.0],  Pol. = Cp,  magnet = -6.001,  T = 1.8 K
S303:  Energy = [390.0, 440.0],  Pol. = Cp,  magnet = -6.000,  T = 1.8 K
S304:  Energy = [390.0, 440.0],  Pol. = Cm,  magnet = -6.000,  T = 1.8 K
S305:  Energy = [680.0, 745.0],  Pol. = Cp,  magnet = 6.000,  T = 1.8 K
S306:  Energy = [680.0, 745.0],  Pol. = Cp,  magnet = 6.000,  T = 1.8 K
S307:  Energy = [680.0, 745.0],  Pol. = Cm,  magnet = 6.000,  T = 1.8 K
S308:  Energy = [680.0, 745.0],  Pol. = Cm,  magnet = 6.000,  T = 1.8 K
S309:  Energy = [680.0, 745.0],  Pol. = Cp,  magnet = 6.000,  T = 1.8 K
S310:  Energy = [680.0, 745.0],  Pol. = Cm,  magnet = 6.000,  T = 1.8 K
S311:  Energy = [680.0, 745.0],  Pol. = Cp,  magnet = 6.000,  T = 1.8 K
S312:  Energy = [680.0, 745.0],  Pol. = Cp,  magnet = 6.000,  T = 1.8 K
S313:  Energy = [680.0, 745.0],  Pol. = Cm,  magnet = 6.000,  T = 1.8 K
S314:  Energy = [390.0, 440.0],  Pol. = Cp,  magnet = 6.000,  T = 1.8 K
S315:  Energy = [390.0, 440.0],  Pol. = Cp,  magnet = 6.000,  T = 1.8 K
S316:  Energy = [390.0, 440.0],  Pol. = Cm,  magnet = 6.000,  T = 1.8 K
S317:  Energy = [390.0, 440.0],  Pol. = Cm,  magnet = 6.000,  T = 1.8 K
S318:  Energy = [390.0, 440.0],  Pol. = Cp,  magnet = 6.000,  T = 1.8 K
S319:  Energy = [390.0, 440.0],  Pol. = Cm,  magnet = 6.000,  T = 1.8 K
S320:  Energy = [390.0, 440.0],  Pol. = Cp,  magnet = 6.000,  T = 1.8 K
S321:  Energy = [390.0, 440.0],  Pol. = Cp,  magnet = 6.000,  T = 1.8 K
S322:  Energy = [390.0, 440.0],  Pol. = Cm,  magnet = 6.000,  T = 1.8 K
S323:  Energy = [680.0, 745.0],  Pol. = Cp,  magnet = 6.000,  T = 1.8 K
S324:  Energy = [680.0, 745.0],  Pol. = Cp,  magnet = 6.000,  T = 1.8 K
S325:  Energy = [680.0, 745.0],  Pol. = Cm,  magnet = 6.000,  T = 1.8 K
S326:  Energy = [680.0, 745.0],  Pol. = Cm,  magnet = 6.001,  T = 1.8 K
S327:  Energy = [680.0, 745.0],  Pol. = Cp,  magnet = 6.000,  T = 1.8 K
S328:  Energy = [680.0, 745.0],  Pol. = Cm,  magnet = 6.001,  T = 1.8 K
S329:  Energy = [680.0, 745.0],  Pol. = Cp,  magnet = 6.001,  T = 1.8 K
S330:  Energy = [680.0, 745.0],  Pol. = Cp,  magnet = 6.001,  T = 1.8 K
S331:  Energy = [680.0, 745.0],  Pol. = Cm,  magnet = 6.001,  T = 1.8 K
S332:  Energy = [390.0, 440.0],  Pol. = Cp,  magnet = 6.001,  T = 1.7 K
S333:  Energy = [390.0, 440.0],  Pol. = Cp,  magnet = 6.001,  T = 1.7 K
S334:  Energy = [390.0, 440.0],  Pol. = Cm,  magnet = 6.001,  T = 1.7 K
S335:  Energy = [390.0, 440.0],  Pol. = Cm,  magnet = 6.001,  T = 1.7 K
S336:  Energy = [390.0, 440.0],  Pol. = Cp,  magnet = 6.001,  T = 1.7 K
S337:  Energy = [390.0, 440.0],  Pol. = Cm,  magnet = 6.001,  T = 1.7 K
S338:  Energy = [390.0, 440.0],  Pol. = Cp,  magnet = 6.001,  T = 1.7 K
S339:  Energy = [390.0, 440.0],  Pol. = Cp,  magnet = 6.001,  T = 1.7 K
S340:  Energy = [390.0, 440.0],  Pol. = Cm,  magnet = 6.001,  T = 1.7 K
S342:  Energy = [770.0, 820.0],  Pol. = Cp,  magnet = 6.001,  T = 2.5 K
S343:  Energy = [770.0, 820.0],  Pol. = Cp,  magnet = 6.001,  T = 2.3 K
S344:  Energy = [770.0, 820.0],  Pol. = Cm,  magnet = 6.001,  T = 2.2 K
S345:  Energy = [770.0, 820.0],  Pol. = Cm,  magnet = 6.001,  T = 2.1 K
S346:  Energy = [770.0, 820.0],  Pol. = Cp,  magnet = 6.001,  T = 2.1 K
S347:  Energy = [770.0, 820.0],  Pol. = Cm,  magnet = 6.001,  T = 2.1 K
S348:  Energy = [770.0, 820.0],  Pol. = Cp,  magnet = 6.001,  T = 2.1 K
S349:  Energy = [770.0, 820.0],  Pol. = Cp,  magnet = 6.001,  T = 2.1 K
```

```
S350:  Energy = [770.0, 820.0],  Pol. = Cm,  magnet = 6.001,  T = 2.1 K
S351:  Energy = [390.0, 440.0],  Pol. = Cp,  magnet = 6.001,  T = 2.1 K
S352:  Energy = [390.0, 440.0],  Pol. = Cp,  magnet = 6.001,  T = 2.0 K
S353:  Energy = [390.0, 440.0],  Pol. = Cm,  magnet = 6.001,  T = 2.0 K
S354:  Energy = [390.0, 440.0],  Pol. = Cm,  magnet = 6.001,  T = 2.0 K
S355:  Energy = [390.0, 440.0],  Pol. = Cp,  magnet = 6.001,  T = 2.0 K
S356:  Energy = [390.0, 440.0],  Pol. = Cm,  magnet = 6.001,  T = 2.0 K
S357:  Energy = [390.0, 440.0],  Pol. = Cp,  magnet = 6.001,  T = 1.9 K
S358:  Energy = [390.0, 440.0],  Pol. = Cp,  magnet = 6.001,  T = 1.9 K
S359:  Energy = [390.0, 440.0],  Pol. = Cm,  magnet = 6.001,  T = 1.9 K
S360:  Energy = [770.0, 820.0],  Pol. = Cp,  magnet = 6.001,  T = 1.8 K
S361:  Energy = [770.0, 820.0],  Pol. = Cp,  magnet = 6.001,  T = 1.8 K
S362:  Energy = [770.0, 820.0],  Pol. = Cm,  magnet = 6.001,  T = 1.8 K
S363:  Energy = [770.0, 820.0],  Pol. = Cm,  magnet = 6.001,  T = 1.8 K
S364:  Energy = [770.0, 820.0],  Pol. = Cp,  magnet = 6.001,  T = 1.8 K
S365:  Energy = [770.0, 820.0],  Pol. = Cm,  magnet = 6.001,  T = 1.8 K
S366:  Energy = [770.0, 820.0],  Pol. = Cp,  magnet = 6.001,  T = 1.8 K
S367:  Energy = [770.0, 820.0],  Pol. = Cp,  magnet = 6.001,  T = 1.8 K
S368:  Energy = [770.0, 820.0],  Pol. = Cm,  magnet = 6.001,  T = 1.8 K
S369:  Energy = [390.0, 440.0],  Pol. = Cp,  magnet = 6.001,  T = 1.8 K
S370:  Energy = [390.0, 440.0],  Pol. = Cp,  magnet = 6.000,  T = 1.8 K
S371:  Energy = [390.0, 440.0],  Pol. = Cm,  magnet = 6.001,  T = 1.8 K
S372:  Energy = [390.0, 440.0],  Pol. = Cm,  magnet = 6.000,  T = 1.8 K
S373:  Energy = [390.0, 440.0],  Pol. = Cp,  magnet = 6.000,  T = 1.8 K
S374:  Energy = [390.0, 440.0],  Pol. = Cm,  magnet = 6.000,  T = 1.8 K
S375:  Energy = [390.0, 440.0],  Pol. = Cp,  magnet = 6.000,  T = 1.8 K
S376:  Energy = [390.0, 440.0],  Pol. = Cp,  magnet = 6.000,  T = 1.8 K
S377:  Energy = [390.0, 440.0],  Pol. = Cm,  magnet = 6.000,  T = 1.8 K
S385:  Energy = [700.0, 730.0],  Pol. = LH,  magnet = -0.000,  T = 300.0 K
S387:  Energy = [760.0, 790.0],  Pol. = LH,  magnet = -0.000,  T = 300.0 K
S391:  Energy = [270.0, 310.0],  Pol. = LH,  magnet = -0.000,  T = 300.0 K
S392:  Energy = [270.0, 330.0],  Pol. = Cp,  magnet = -0.000,  T = 300.0 K
S393:  Energy = [270.0, 330.0],  Pol. = Cp,  magnet = -0.000,  T = 300.0 K
S394:  Energy = [270.0, 330.0],  Pol. = Cm,  magnet = -0.000,  T = 300.0 K
S395:  Energy = [270.0, 330.0],  Pol. = Cm,  magnet = -0.000,  T = 300.0 K
S396:  Energy = [270.0, 330.0],  Pol. = Cp,  magnet = -0.000,  T = 300.0 K
S397:  Energy = [270.0, 330.0],  Pol. = Cm,  magnet = -0.000,  T = 300.0 K
S398:  Energy = [270.0, 330.0],  Pol. = Cp,  magnet = -0.000,  T = 300.0 K
S399:  Energy = [270.0, 330.0],  Pol. = Cp,  magnet = -0.000,  T = 300.0 K
S400:  Energy = [270.0, 330.0],  Pol. = Cm,  magnet = -0.000,  T = 300.0 K
S401:  Energy = [270.0, 330.0],  Pol. = Cp,  magnet = -0.000,  T = 300.0 K
S402:  Energy = [270.0, 330.0],  Pol. = Cp,  magnet = -0.000,  T = 300.0 K
S403:  Energy = [270.0, 330.0],  Pol. = Cm,  magnet = -0.000,  T = 300.0 K
S404:  Energy = [270.0, 330.0],  Pol. = Cm,  magnet = -0.000,  T = 300.0 K
S405:  Energy = [270.0, 330.0],  Pol. = Cp,  magnet = -0.000,  T = 300.0 K
S406:  Energy = [270.0, 330.0],  Pol. = Cm,  magnet = -0.000,  T = 300.0 K
S407:  Energy = [270.0, 330.0],  Pol. = Cp,  magnet = -0.000,  T = 300.0 K
S408:  Energy = [270.0, 330.0],  Pol. = Cp,  magnet = -0.000,  T = 300.0 K
S409:  Energy = [270.0, 330.0],  Pol. = Cm,  magnet = -0.000,  T = 300.0 K
S410:  Energy = [770.0, 810.0],  Pol. = Cp,  magnet = -0.000,  T = 300.0 K
S411:  Energy = [770.0, 810.0],  Pol. = Cp,  magnet = -0.000,  T = 300.0 K
```

Loading data

In [26]:
```
numids = range(178, 185)  # range(n, m) = [n, n+1, ..., m-2, m-1]
mentry = load_data(file_path, numids)
```

```
-----------------------------
|   Loading single-entry    |
-----------------------------
Found S 178:
  Energy = [840.0, 895.0],  Pol. = Cm,  magnet = -6.001,  T = 2.2 K
Found S 179:
  Energy = [840.0, 895.0],  Pol. = Cm,  magnet = -6.001,  T = 2.1 K
Found S 180:
  Energy = [840.0, 895.0],  Pol. = Cp,  magnet = -6.001,  T = 2.2 K
Found S 181:
  Energy = [840.0, 895.0],  Pol. = Cm,  magnet = -6.001,  T = 2.2 K
Found S 182:
  Energy = [840.0, 895.0],  Pol. = Cp,  magnet = -6.001,  T = 2.2 K
Found S 183:
  Energy = [840.0, 895.0],  Pol. = Cp,  magnet = -6.001,  T = 2.2 K
Found S 184:
  Energy = [840.0, 895.0],  Pol. = Cm,  magnet = -6.001,  T = 2.1 K

-----------------------------
| Generating multiple-entry |
-----------------------------
Detected the following polarizations:
   ['Cm', 'Cm', 'Cp', 'Cm', 'Cp', 'Cp', 'Cm']
Grouped as: ['Cm', 'Cp']
Detected Cp and Cm so XMCD data.

  -------------------
  |    XMCD data    |
  -------------------
  Generating data['mu_p'], data['mu_m'], data['diff']  and data['sum']:
  data['diff'] = data['mu_p'] - data['mu_m']

  data['sum'] = data['mu_p'] + data['mu_m']
```

Plot of the data

data as after loading

In [27]:
```
mentry.plt_report()
plt.axhline(0, color='k', linewidth=0.75)
```

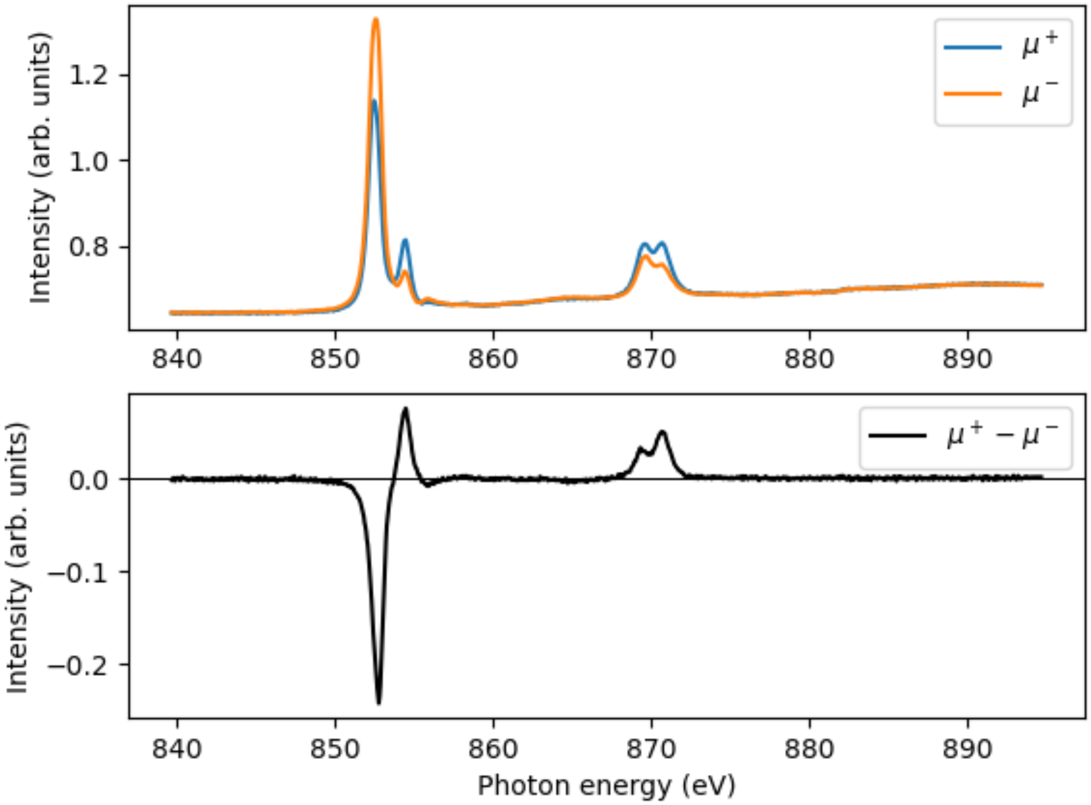

Out[27]:
```
<matplotlib.lines.Line2D at 0x7fd364b24b20>
```

If diff not fine, it is possible to tune it.

The condition is to have the same average values before and after the XAS peak. The region are:

```
[e_min-e_delta : e_min+e_delta]
[e_max-e_delta : e_max+e_delta]
```

In [28]:
```
mentry.xmcd_align(e_min=845, e_max=880, e_delta=2)
mentry.plt_report()
plt.axhline(0, color='k', linewidth=0.75)
```

```
[844.99680324 879.97986228]

  data['mu_p'] aligned up data['mu_m']

  data['diff'] = data['mu_p'] - data['mu_m']

  data['sum'] = data['mu_p'] + data['mu_m']
```

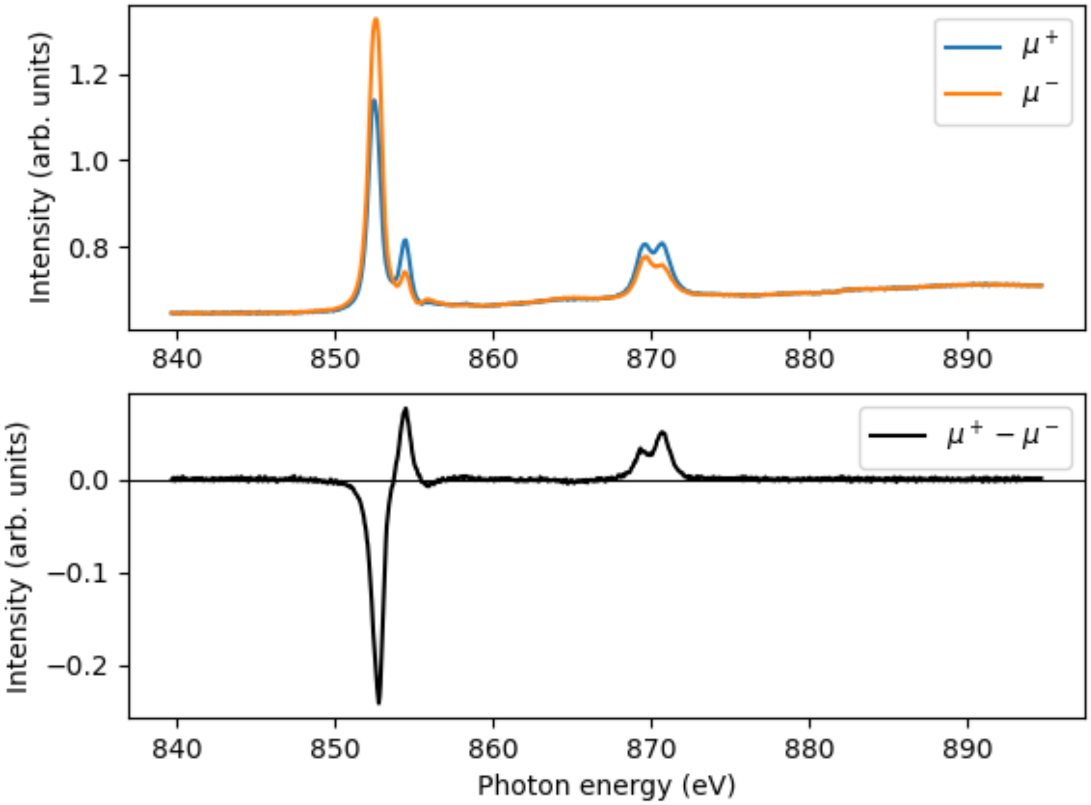

Out[28]: <matplotlib.lines.Line2D at 0x7fd3662a7eb0>

Removing linear background

In [29]:
```
mentry.rmv_bkg(e_min=845)
mentry.plt_report()
```

```
data['sum'] = data['mu_p'] + data['mu_m']
```

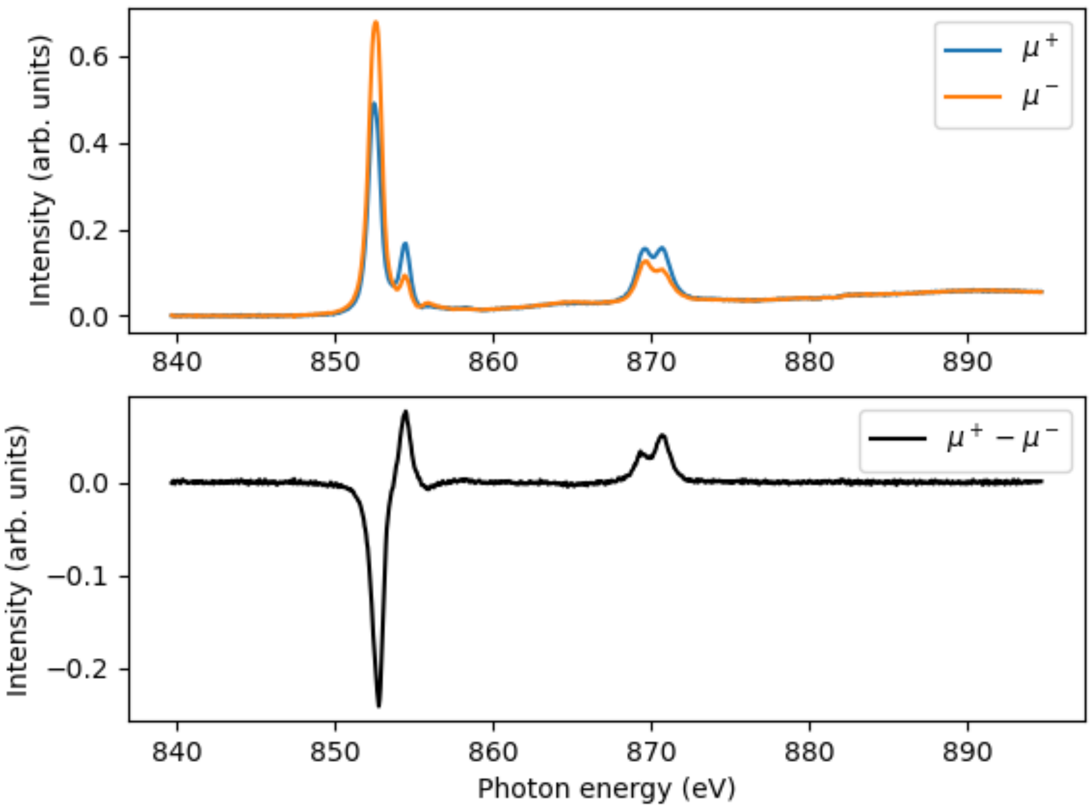

Sum rule

Calculating p and q

In [30]:

```
# input
e_min = 845   # starting energy for integral curve of XMCD
e_max = 890   # final energy for integral curve of XMCD

en_p = 860   # energy where to calculate p, integral of L3 XMCD
en_q = 880   # energy where to calculate q, integral of L3+L2 XMCD

# procedure
## calculate integral of xmcd
ind_range, = np.where((mentry.energy > e_min) &
                      (mentry.energy < e_max))
delta_en = np.diff(mentry.energy[ind_range]).min()
xmcd = np.cumsum(mentry.data['diff'][ind_range])*delta_en

## determination of p and q
ind_en_p = np.argmin(abs(mentry.energy[ind_range]-en_p))
ind_en_q = np.argmin(abs(mentry.energy[ind_range]-en_q))
xmcd_p = xmcd[ind_en_p]
xmcd_q = xmcd[ind_en_q]
en_p = mentry.energy[ind_range][ind_en_p]
en_q = mentry.energy[ind_range][ind_en_q]
print("p, q = {:0.3f}, {:0.3f}".format(xmcd_p, xmcd_q))

## plot
plt.figure()
plt.axhline(0, color='k', linewidth=0.75)
plt.plot(mentry.energy[ind_range], xmcd, label="$\int \mu^+ - \mu^-$")
plt.plot(mentry.energy[ind_range], mentry.data['diff'][ind_range],
         label="$\mu^+ - \mu^-$")
plt.legend(loc='lower right')
plt.xlabel('Photon energy (eV)')
plt.ylabel('Intensity (arb. units)')

for en_pq, xmcd_pq, label in zip([en_p, en_q],
                                 [xmcd_p, xmcd_q],
                                 ['p', 'q']):
    plt.annotate('', xy=(en_pq, xmcd_pq), xytext=(en_pq, 0),arrowprops=dict(
    plt.text(en_pq+1, xmcd_pq*0.5, s=label, size=12)
```

```
p, q = -0.179, -0.087
```

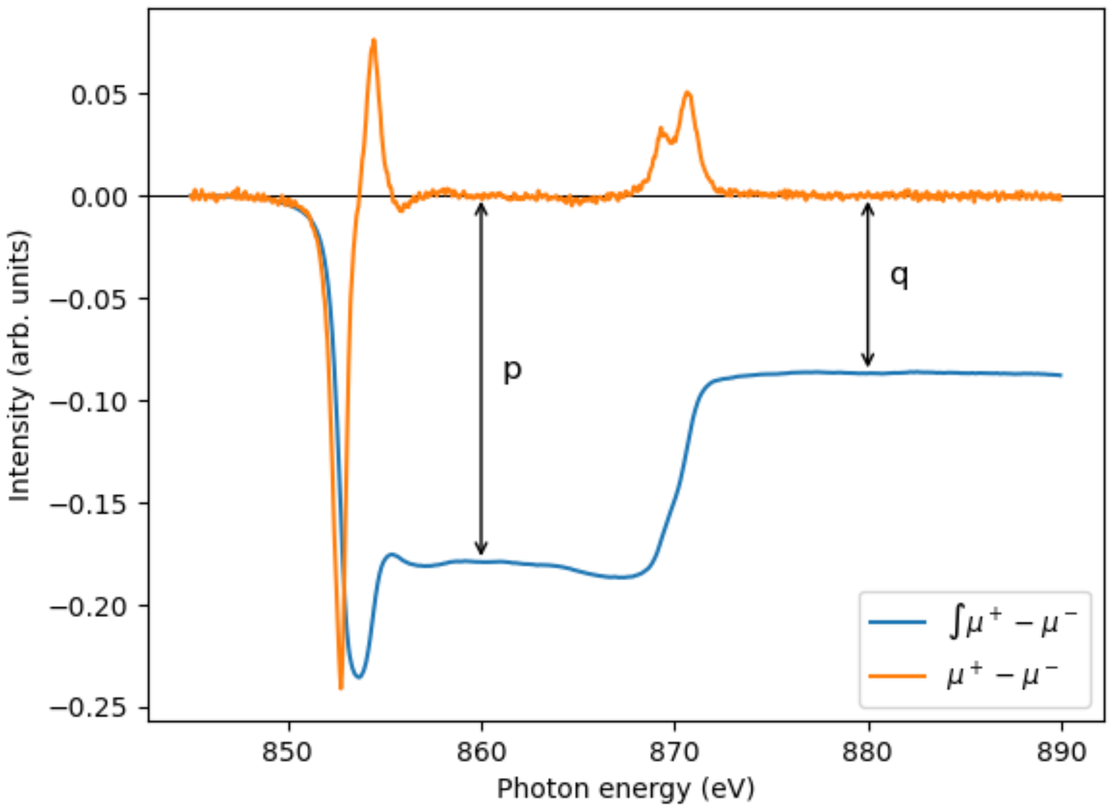

Calculating r

In [31]:

```python
# input
en_range = np.array([845, 875]) #energy range of XAS integral

ep1 = 852.4 # L3 white line energy position
ep2 = 869.7 # L2 white line energy position
amp = 0.072 # total height of the two edge jumps
const = 0 # vertical rigid shift of edge jump
sigma = 0.5 # broadening of the step of the edge jump
ratio = 2 # L3/L2 height ratio of the two edge jumps
eshift = 0 # energy shift of edge jumps with respect to the white lines

pars = ep1, ep2, amp, const, sigma, ratio, eshift

# procedure
## calculate step function
step_bkg = step_fun(mentry.energy, pars, model='arctan')
## calculate integral xas (r)
ind_range, = np.where((mentry.energy > en_range.min()) &
                      (mentry.energy < en_range.max()))
delta_en = np.diff(mentry.energy).min()
xas_r = np.sum(mentry.data['sum'][ind_range]-step_bkg[ind_range])*delta_en
print("Int_XAS = r = {:0.3f}".format(xas_r))

## plot
plt.figure()
plt.plot(mentry.energy, mentry.data['sum'], label='XAS')
plt.plot(mentry.energy, step_bkg, label='background')
plt.legend()
plt.xlabel('Photon energy (eV)')
plt.ylabel('Intensity (arb. units)')
```

```
Int_XAS = r = 1.957
```

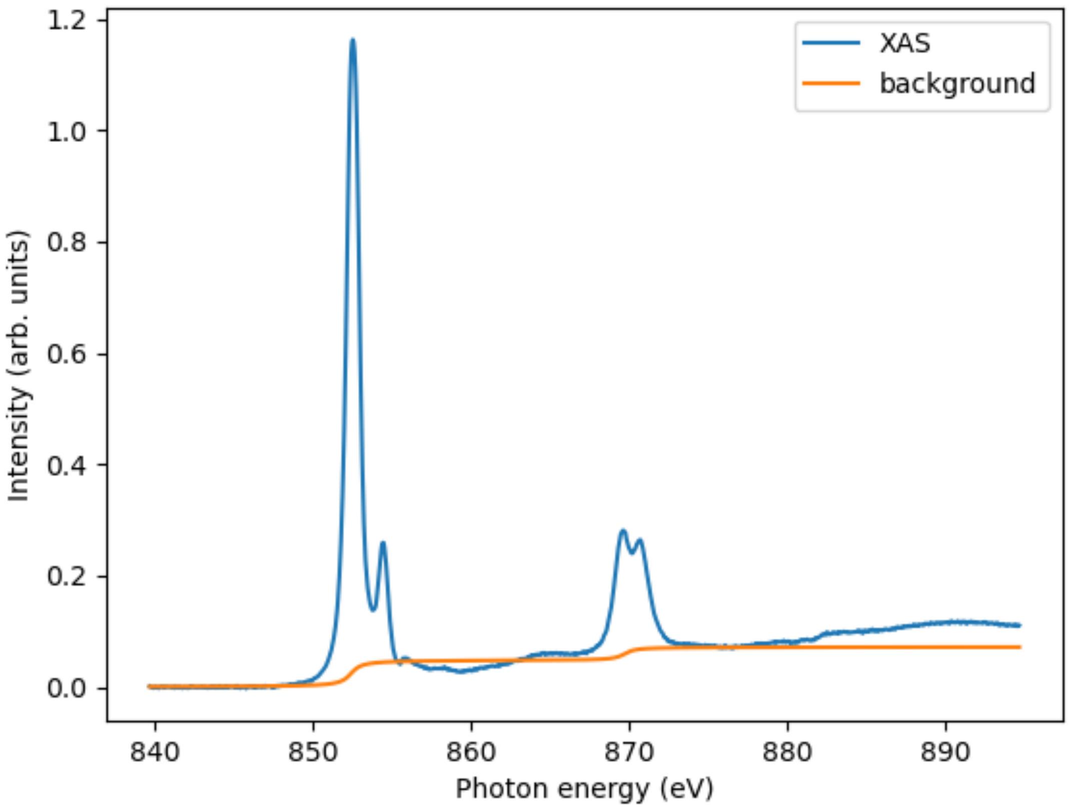

Out[31]:
```
Text(0, 0.5, 'Intensity (arb. units)')
```

Result sum rule

$$< L_z >= \frac{4q(10-n)}{3r}$$

$$< S_{eff} >=< S_z > + \frac{7}{2} < T_z >= \frac{(3p-2q)(10-n)}{r}$$

where n is the number of electron.

In [32]:
```
n_occ = 8  # number of electrons in the d band
L_z = -4*xmcd_q * (10-n_occ) / (3*xas_r)
S_eff = -(3*xmcd_p - 2*xmcd_q)*(10-n_occ) / xas_r

print("L_z = {:0.3f}".format(L_z))
print("S_eff = {:0.3f}".format(S_eff))
```

```
L_z = 0.118
S_eff = 0.371
```

Saving the data as a txt file (default name = "MultiEntryXAS.dat")

In [33]:
```
mentry.save_data("Verdazyl_NiL23_25K.dat")
```

In [ ]:
```
```

In [ ]:
```
```
