# Supplementary material for: Metal-ligand interactions in a redox active ligand system. Electrochemistry and spectroscopy of [M(dipyvd)2]n+ (M=Zn, Ni, n=0, 1, 2)
Source: Front Chem. 2023 Nov 15;11:1295289. doi: 10.3389/fchem.2023.1295289 (PMC10684738; doi:10.3389/fchem.2023.1295289)

checkCIF/PLATON report

Structure factors have been supplied for datablock(s) zn_dipyvd_2p_remeasure_0m

THIS REPORT IS FOR GUIDANCE ONLY. IF USED AS PART OF A REVIEW PROCEDURE FOR PUBLICATION, IT SHOULD NOT REPLACE THE EXPERTISE OF AN EXPERIENCED CRYSTALLOGRAPHIC REFEREE.

No syntax errors found. CIF dictionary Interpreting this report

Datablock: zn_dipyvd_2p_remeasure_0m

```
Bond precision:   C-C = 0.0131 A          Wavelength=0.71073

Cell:              a=15.8591(6)      b=15.8591(6)      c=15.6583(8)
                   alpha=90          beta=90           gamma=90
Temperature:       299 K

                 Calculated                    Reported
Volume           3938.2(4)                     3938.2(4)
Space group      P -4 21 c                     P -4 21 c
Hall group       P -4 2n                       P -4 2n
Moiety formula   C30 H30 N12 O2 Zn, 2(F6 P)  C30 H30 N12 O2 Zn, 2(F6 P)
Sum formula      C30 H30 F12 N12 O2 P2 Zn    C30 H30 F12 N12 O2 P2 Zn
Mr               945.99                        945.97
Dx,g cm-3        1.595                         1.595
Z                4                             4
Mu (mm-1)        0.809                         0.809
F000             1912.0                        1912.0
F000'            1915.10
h,k,lmax         18,18,18                      18,18,18
Nref             3373[ 1889]                   3373
Tmin,Tmax        0.907,0.922                   0.683,0.745
Tmin'            0.851

Correction method= # Reported T Limits: Tmin=0.683 Tmax=0.745
AbsCorr = MULTI-SCAN

Data completeness= 1.79/1.00       Theta(max)= 24.726

R(reflections)= 0.0565( 2585)                       wR2(reflections)=
                                                    0.1225( 3373)

S = 1.155                    Npar= 299
```

The following ALERTS were generated. Each ALERT has the format
test-name_ALERT_alert-type_alert-level.
Click on the hyperlinks for more details of the test.

Alert level A

PLAT213_ALERT_2_A Atom C13B has ADP max/min Ratio ..... 7.0 prolat

Author Response: Atom C13B (along with C13A, C14A/B and C15A/B) are part of a disordere Attempts to model the disorder result in elongated ellipsoids for these atoms.

Alert level B

PLAT213_ALERT_2_B Atom C15A has ADP max/min Ratio ..... 4.8 prolat

Author Response: Atom C13B (along with C13A, C14A/B and C15A/B) are part of a disordere Attempts to model the disorder result in elongated ellipsoids for these atoms.

Alert level C

STRVA01_ALERT_4_C Flack test results are ambiguous.
From the CIF: _refine_ls_abs_structure_Flack 0.480
From the CIF: _refine_ls_abs_structure_Flack_su 0.040
THETM01_ALERT_3_C The value of sine(theta_max)/wavelength is less than 0.590
Calculated sin(theta_max)/wavelength = 0.5885
PLAT090_ALERT_3_C Poor Data / Parameter Ratio (Zmax > 18) .... 6.31 Note
PLAT213_ALERT_2_C Atom C13A has ADP max/min Ratio ..... 4.0 prolat

Author Response: Atom C13B (along with C13A, C14A/B and C15A/B) are part of a disordere Attempts to model the disorder result in elongated ellipsoids for these atoms.

PLAT213_ALERT_2_C Atom C14A has ADP max/min Ratio ..... 3.2 oblate

Author Response: Atom C13B (along with C13A, C14A/B and C15A/B) are part of a disordere Attempts to model the disorder result in elongated ellipsoids for these atoms.

PLAT213_ALERT_2_C Atom C15B has ADP max/min Ratio ..... 3.9 prolat

Author Response: Atom C13B (along with C13A, C14A/B and C15A/B) are part of a disordere Attempts to model the disorder result in elongated ellipsoids for these atoms.

```
PLAT220_ALERT_2_C NonSolvent   Resd 1  C   Ueq(max)/Ueq(min) Range        5.7 Ratio
PLAT222_ALERT_3_C NonSolvent Resd 1  H   Uiso(max)/Uiso(min) Range        5.8 Ratio
PLAT250_ALERT_2_C Large U3/U1 Ratio for Average U(i,j) Tensor ....        2.9 Note
PLAT260_ALERT_2_C Large Average Ueq of Residue Including       P1      0.126 Check
PLAT341_ALERT_3_C Low Bond Precision on  C-C Bonds ..............    0.01313 Ang.
PLAT906_ALERT_3_C Large K Value in the Analysis of Variance ......      5.370 Check
```

Alert level G

```
PLAT003_ALERT_2_G Number of Uiso or Uij Restrained non-H Atoms ...           6 Report
PLAT168_ALERT_4_G The CIF-Embedded .res File Contains EXYZ Records           4 Report
PLAT171_ALERT_4_G The CIF-Embedded .res File Contains EADP Records           4 Report
PLAT186_ALERT_4_G The CIF-Embedded .res File Contains ISOR Records           1 Report
PLAT244_ALERT_4_G Low    'Solvent' Ueq as Compared to Neighbors of          P1 Check
PLAT301_ALERT_3_G Main Residue  Disorder ..............(Resd  1  )         36% Note
PLAT432_ALERT_2_G Short Inter X...Y Contact  F1       ..C2A      .        2.96 Ang.
                                            1-x,1-y,z  =      3_665 Check
PLAT722_ALERT_1_G Angle   Calc      108.00, Rep      109.10 Dev...        1.10 Degree
              H14D    -C14B    -H14F       1_555  1_555  1_555     #   29 Check
PLAT722_ALERT_1_G Angle   Calc      111.00, Rep      109.60 Dev...        1.40 Degree
              H15D    -C15B    -H15E       1_555  1_555  1_555     #   34 Check
PLAT802_ALERT_4_G CIF Input Record(s) with more than 80 Characters           1 Info
PLAT811_ALERT_5_G No ADDSYM Analysis: Too Many Excluded Atoms ....           ! Info
PLAT860_ALERT_3_G Number of Least-Squares Restraints ............          36 Note
PLAT909_ALERT_3_G Percentage of I>2sig(I) Data at Theta(Max) Still         53% Note
PLAT910_ALERT_3_G Missing # of FCF Reflection(s) Below Theta(Min).           2 Note
PLAT978_ALERT_2_G Number C-C Bonds with Positive Residual Density.           0 Info
```

```
   1 ALERT level A = Most likely a serious problem - resolve or explain
   1 ALERT level B = A potentially serious problem, consider carefully
  12 ALERT level C = Check. Ensure it is not caused by an omission or oversight
  15 ALERT level G = General information/check it is not something unexpected

   2 ALERT type 1 CIF construction/syntax error, inconsistent or missing data
  11 ALERT type 2 Indicator that the structure model may be wrong or deficient
   9 ALERT type 3 Indicator that the structure quality may be low
   6 ALERT type 4 Improvement, methodology, query or suggestion
   1 ALERT type 5 Informative message, check
```

It is advisable to attempt to resolve as many as possible of the alerts in all categories. Often the minor alerts point to easily fixed oversights, errors and omissions in your CIF or refinement strategy, so attention to these fine details can be worthwhile. In order to resolve some of the more serious problems it may be necessary to carry out additional measurements or structure refinements. However, the purpose of your study may justify the reported deviations and the more serious of these should normally be commented upon in the discussion or experimental section of a paper or in the "special_details" fields of the CIF. checkCIF was carefully designed to identify outliers and unusual parameters, but every test has its limitations and alerts that are not important in a particular case may appear. Conversely, the absence of alerts does not guarantee there are no aspects of the results needing attention. It is up to the individual to critically assess their own results and, if necessary, seek expert advice.

Publication of your CIF in IUCr journals

A basic structural check has been run on your CIF. These basic checks will be run on all CIFs submitted for publication in IUCr journals (*Acta Crystallographica*, *Journal of Applied Crystallography*, *Journal of Synchrotron Radiation*); however, if you intend to submit to *Acta Crystallographica Section C* or *E* or *IUCrData*, you should make sure that full publication checks are run on the final version of your CIF prior to submission.

Publication of your CIF in other journals

Please refer to the *Notes for Authors* of the relevant journal for any special instructions relating to CIF submission.

PLATON version of 06/07/2023; check.def file version of 30/06/2023

Datablock zn_dipyvd_2p_remeasure_0m - ellipsoid plot

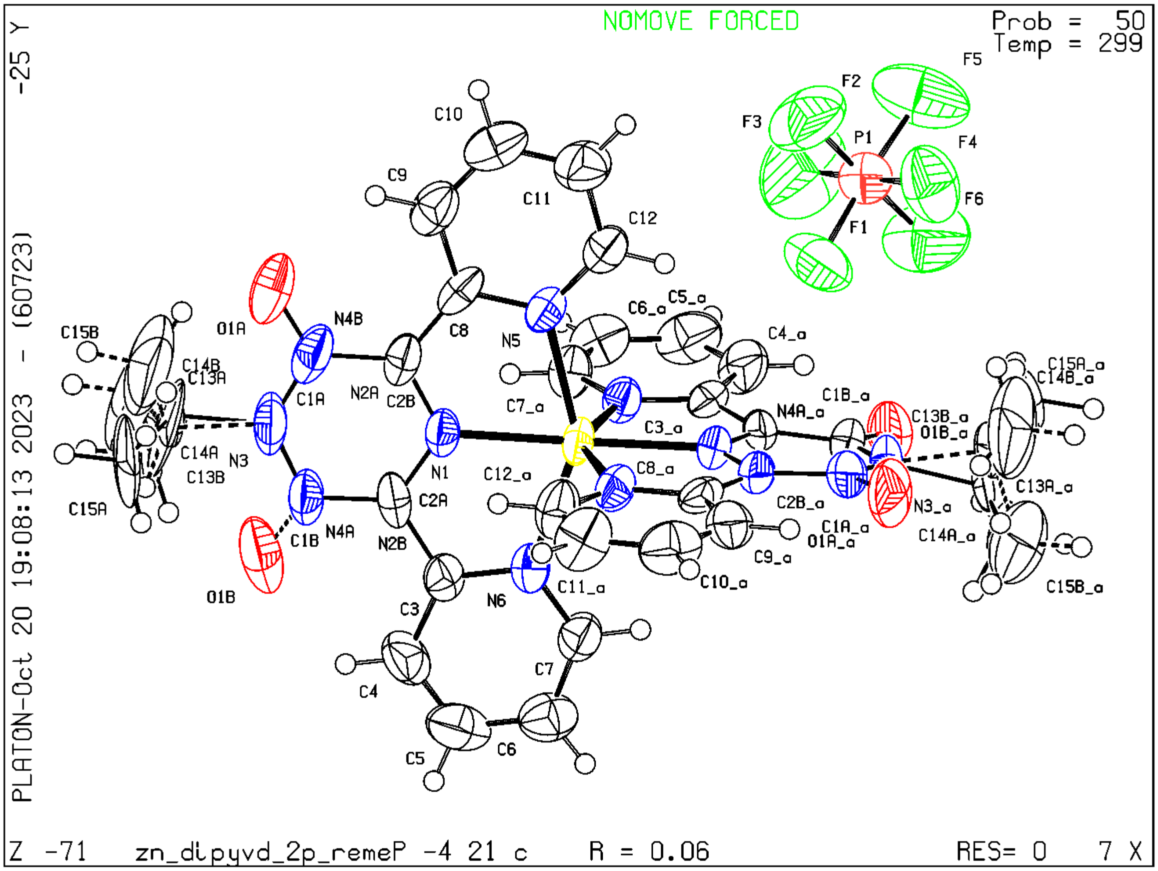

Supplement: Supplementary file 2 [file DataSheet4.PDF]
